# Laccase-Catalyzed 1,4-Dioxane-Mediated Synthesis of Belladine *N*-Oxides with Anti-Influenza A Virus Activity

**DOI:** 10.3390/ijms22031337

**Published:** 2021-01-29

**Authors:** Claudio Zippilli, Lorenzo Botta, Bruno Mattia Bizzarri, Lucia Nencioni, Marta De Angelis, Virginia Protto, Gianluca Giorgi, Maria Camilla Baratto, Rebecca Pogni, Raffaele Saladino

**Affiliations:** 1 Department of Ecology and Biology, University of Tuscia, 01100 Viterbo, Italy; zippillic@unitus.it (C.Z.); lorenzo.botta@unitus.it (L.B.); bm.bizzarri@unitus.it (B.M.B.); 2 Department of Public Health and Infectious Diseases, Laboratory affiliated to Istituto Pasteur Italia, Fondazione Cenci Bolognetti, Sapienza University of Rome, 00185 Rome, Italy; marta.deangelis@uniroma1.it (M.D.A.); virginia.protto@uniroma1.it (V.P.); 3 Department of Biotechnology, Chemistry and Pharmacy, University of Siena, Via Aldo Moro 2, 53100 Siena, Italy; gianluca.giorgi@unisi.it (G.G.); mariacamilla.baratto@unisi.it (M.C.B.); rebecca.pogni@unisi.it (R.P.)

**Keywords:** Oxizymes, laccase, belladine derivatives, *N*-oxides, influenza A virus, EPR, 1,4-dioxane

## Abstract

Belladine *N*-oxides active against influenza A virus have been synthetized by a novel laccase-catalyzed 1,4-dioxane-mediated oxidation of aromatic and side-chain modified belladine derivatives. Electron paramagnetic resonance (EPR) analysis confirmed the role of 1,4-dioxane as a co-oxidant. The reaction was chemo-selective, showing a high functional-group compatibility. The novel belladine *N*-oxides were active against influenza A virus, involving the early stage of the virus replication life cycle.

## 1. Introduction

**Figure 1:**
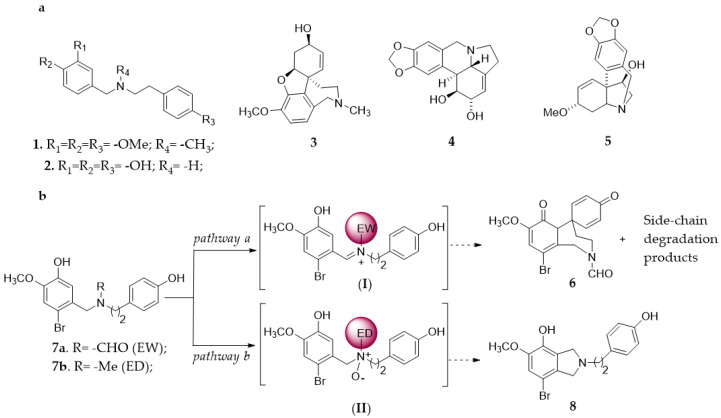
Panel (**a**): chemical structure of belladine **1**, norbelladine **2**, galantamine **3**, lycorine **4**, and haemanthamine **5**. Panel (**b**): side-reactions observed during the oxidation of *N*-substituted 2-bromo-*O*-methylnorbelladines **7a**–**b** with laccase from *Trametes versicolor*. *Pathway a*: formation of the iminium ion intermediate (**I**) in the presence of electron-withdrawing (EW) substituent and successive transformation to spyrocyclohexadienone **6**. *Pathway b*: formation of the *N*-oxide intermediate (**II**) in the presence of electron-donating (ED) substituent followed by phenolic intramolecular cycloaddition to isoindoline **8**.

Belladine **1** and norbelladine **2** (firstly extracted from the Amaryllidaceae family [1]) are bioactive precursors in the synthesis of drugs acting on the central nervous system, such as galantamine **3**, lycorine **4**, and haemanthamine **5** (Figure 1, Panel a) [2,3]. They are natural substances emerging in therapy, showing a cholinesterase inhibitory activity comparable to that of 3 in the treatment of Alzheimer’s disease [4,5]. In addition, a computational study suggested that quaternary belladine derivatives can interact with the neuroaminidase (NA) protein of influenza A virus, inhibiting viral release from host cell [6].

Recently, the use of *N*-formyl-2-bromo-*O*-methynorbelladine **7a** in the total synthesis of **3** by a laccase (benzenediol: oxygen oxidoreductases, EC 1.10.3.2) [7]-mediator system has been reported, focusing on the formation of the spyrocyclohexadienone **6** as a tri-cyclic intermediate (Figure 1, Panel b) [8]. In this latter case, undesired side-products were produced depending on the nature of the *N*-substituent. Electron withdrawing group EW (R = CHO, **7a**) favored the formation of phenoxy radicals and successive oxidative coupling, and the hydrolysis of the iminium ion (**I**) to side-chain degradation products was the only observed side-process [8,9] (Figure 1; Panel b, pathway a). Conversely, the oxidative coupling was not operative with electron-donating group ED (R = CH_3_, **7b**), in which case the isoindoline **8** was produced by an iminium-ion Polonovski transformation of the *N*-oxide intermediate (**II**) (not isolated) [10,11] (Figure 1; Panel b, pathway b).

Amine *N*-oxides are widely diffused in nature [12,13], and they play an important role as chiral ligands, organo-catalysts, and synthons [14,15]. These compounds are synthetized using hazardous stoichiometric oxidants, or in the alternative, heavy metal catalysts and hydrogen peroxide (H_2_O_2_), which leads to the formation of toxic wastes and undesired by-products [16]. As an alternative, dioxygen (O_2_) is an effective green oxidant [17,18,19] when associated with laccases, favoring the formation of reactive singlet-state species in energy barrierless and green conditions. Laccases are low-cost enzymes with high catalytic activity, broad substrate specificity, and beneficial chemical and physical properties [20,21,22,23,24,25,26,27,28,29]. To the best of our knowledge, only one example of oxidation of tertiary amines by laccase has been reported; however, the yield and selectivity were low [30].

Here we describe the efficient synthesis of belladine *N*-oxides **9** and **12a**–**h** (Table 2) by use of laccase from *Trametes versicolor*. The control of the pH of the reaction and the use of 1,4-dioxane as co-oxidant favored the stabilization of intermediate (**II**) by inhibition of the Polonovski transformation (Figure 1; Panel b, pathway b), affording the desired *N*-oxides in high yield and regio-selectivity. A modest stereo-selectivity was also observed by the use of chiral shift reagent europium tris-[3-(heptafluoropropylhydroxymethylene)-(+)-camphorate] (Eu(hfc)_3_) salt. Belladine *N*-oxides were active against Influenza A virus, and compound **12h** showed the highest activity.

## 2. Results and Discussion

### 2.1. Optimization of the Reaction Conditions

**Scheme 1:**
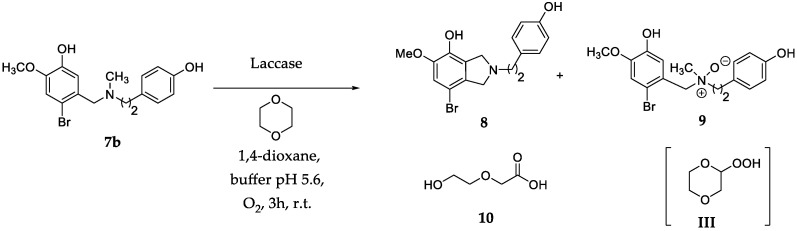
Synthesis of belladine *N*-oxide derivative **9** from **7b.** Isoindoline **8** and 2-(2-hydroxyethoxy)acetic acid **10** were isolated as by-products.

The Polonovski transformation [11] of amine *N*-oxides occurs by two successive steps: (a) the protonation of the quaternary *N*-oxide moiety [31,32]; and (b) the cleavage of the iminium ion to corresponding aminium radical cation, followed by α-hydrogen elimination and skeletal rearrangement [33,34]. In order to avoid the occurrence of the Polonovski reaction in the laccase catalyzed synthesis of belladine *N*-oxides, the critical reaction step was expected to be the protonation of the *N*-oxide moiety. We started our investigation using **7b** (Scheme 1) as a model substrate (general procedures are in Supplementary Materials (SM) #1, and the synthesis of **7b** in SM #2).

The treatment of **7b** (0.1 mmol) with laccase (1000 U·mmol^−1^) and TEMPO (2,2,6,6-tetra methyl-1-piperidinyloxy free radical, 0.06 mmol.) [35,36] at 25 °C for 3.0 h under O_2_ atmosphere in 1,4-dioxane (0.5 mL) and sodium acetate buffer (2.0 mL; 0.5 M; pH 4.5) afforded **8** as the only recovered product, besides the unreacted substrate (Scheme 1; Table 1, entry 1) [8]. Examples of the retained activity of laccase in organic solvents are reported, and their advantages for the selectivity of the transformation are adequately discussed [37,38]. The oxidation with a lower amount of laccase (100 U·mmol^−1^) in the absence of TEMPO afforded a tiny amount of *N*-oxide **9**, alongside **8** (Scheme 1; Table 1, entry 2). Better results were obtained at pH 5.6; in this case, **9** was isolated in 28% yield (Table 1, entry 3) (NMR data of **9** are in SM #3). The reaction showed a similar behavior at higher pH. The yield of **9** was further increased by increasing the amount of 1,4-dioxane (1,4-dioxane: buffer 10:1). In this latter case, 2-(2-hydroxyethoxy)acetic acid **10** was isolated as a by-product (Scheme 1, Table 1, entry 4; NMR data of 10 are in SM #3). In addition, **9** was obtained in 56% yield using 1,4-dioxane deprived of the commercial radical scavenger butyl hydroxytoluene (BHT) (Table 1, entry 5). The use of tetrahydrofuran (THF) and acetonitrile (CH_3_CN) as alternative solvents was not effective (Table 1, entry 6 and 7, respectively).

### 2.2. EPR Studies

**Figure 2:**
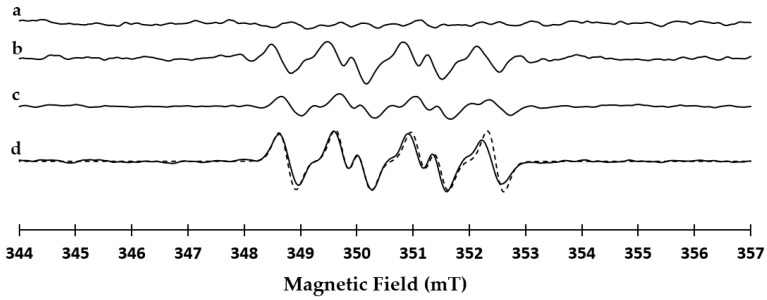
Line (**a**): CW (continuous wave) X-band (9 GHz) EPR spectrum at room temperature of 5,5-dimethyl-1-pyrroline *N*-oxide (DMPO) and dioxane; line (**b**): CW X-band (9 GHz) EPR spectrum at room temperature of DMPO, dioxane, and laccase; line (**c**): CW X-band (9 GHz) EPR spectrum at room temperature of DMPO, dioxane, laccase, and **7b**; line (**d**): CW X-band (9 GHz) EPR spectrum at room temperature of DMPO, dioxane, laccase and **11h** (bold line) paired to it simulation (dotted line). The spectra reported in line **a** and **b** were acquired at t = 0 min and those in line **c** and **d** at t = 180 min.

Compound **10** is reported to be the ring-opening product of 2-hydroperoxy-1,4-dioxane **III** (not isolated, Scheme 1) [39,40,41]. EPR studies with 5,5-dimethyl-1-pyrroline *N*-oxide (DMPO) confirmed the presence of **III** in the reaction mixture. As reported in Figure 2 (line a), 1,4-dioxane alone showed a tiny signal in the magnetic field range of 348–353 mT compatible with the formation of the **III**/DMPO-adduct. The intensity of this signal increased after the addition of laccase (line b). In addition, the same signal was detected during the oxidation of **7b** with laccase (line c). The III/DMPO-adduct was common to all cases studied with increasing intensity after the addition of laccase. From a spectroscopic point of view, the spin-trapping approach allows to trap the first radical species formed in the reaction, and the increase of intensity of the signal at 348–353 mT confirms the role of 1,4-dioxane as co-oxidant in the oxidation.

### 2.3. Synthesis and Characterization of Belladine N-Oxide Derivatives ***12a**–**h***

The procedure was generalized to derivatives **11a**–**h**, covering a large panel of substituents in the aromatic ring and side chain (the synthesis of compounds **11a**–**h** is in SM #2; NMR data of **11a**–**h** are in SM #3). Compounds **11a**–**h** (0.1 mmol) were treated with laccase (100 U·mmol^−1^) under O_2_ atmosphere in 10:1 ratio 1,4-dioxane/sodium acetate buffer (2.50 mL; pH 5.6) at 25 °C for 3.0 h to afford *N*-oxides **12a**–**h** from good to high yield (53–78%), besides to unreacted substrate (Table 2, entries 1–9) (NMR data of **12a**–**h** are in SM #3). Isoindolines were not detected in the reaction mixture. All type of substituent patterns and side-chain length were allowed, highlighting the high chemo-selectivity and functional-group compatibility of the procedure. A further evidence of the **III**/DMPO-adduct is reported in Figure 2 (line **d**) where the EPR signal recorded during the oxidation of **11h** is paired to its simulation. The intensity of the signal is higher than in the previous case. The magnetic parameters obtained from the fitting are: g = 2.0061 ± 0.0001, A_N_ = 1.36 mT, A_H_ = 1.01 mT and A_H_ = 0.117 mT. These parameters are typical of peroxyl radical adduct with the DMPO in organic solvents [42]. The intensity of this signal was higher than that previously observed in the oxidation of **7b**, in accordance with the higher yield of **12h** with respect to **9**.

**Figure 3:**
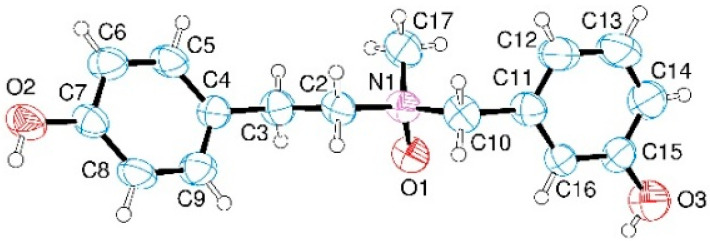
Crystal structure of **12b**. Ellipsoids enclose 50% probability.

X-ray data confirmed the structure of **12b**, which was the only product isolated as a crystal (Figure 3). The compound crystallizes in a centric space group (C2/c) containing both the enantiomers (for details of the X-ray analysis and crystallization procedure see the Section Materials and Methods).

**Figure 4:**
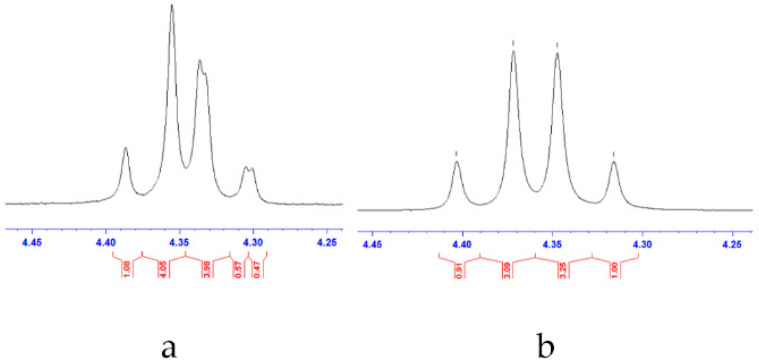
Panel (**a**): ^1^H-NMR spectra of compound **12b** in the presence of Eu(hfc)_3_; panel (**b**): ^1^H-NMR spectra of compound **12b** in the absence of Eu(hfc)_3_.

As a selected case of study, the ^1^H-NMR of **12b** with chiral lanthanide shift reagent Eu(hfc)_3_ (700 µL MeOD, 13.9 mM Eu(hfc)_3_) [43] showed the expected asymmetric shift pattern of the AB quartet system (4.60–4.20 ppm) for the resolution of the two enantiomers, with an enantiomeric excess (ee) of 10% (Figure 4). Polarimetric analyses of **9**, **12a**–**e** and **12g**–**h** are reported in Table 2.

### 2.4. Antiviral Activity of Compound ***7b*** and ***12a**–**h*** against Influenza A Virus

Compounds **9** and **12a**–**h** were tested against influenza A/Puerto Rico/8/34 H1N1 (PR8) virus in order to evaluate previously reported computational hypothesis about the inhibition of viral NA [6]. Influenza is responsible for large epidemics and pandemics causing severe health problems [44]. The influenza A virus (*Orthomyxoviridae* family) is characterized by the release of eight viral RNA segments associated with the nucleoprotein (NP) and the viral RNA-dependent RNA polymerase (RdRp) complex responsible for replication and transcription cycles [45]. Among the inhibitors of the influenza A virus, the compounds active against NA received great attention being involved in the release of viral particles from infected cells [46,47]. In the first set of experiments, A549 cells infected with 0.001 MOI of PR8 were treated with different concentrations (range 10–40 mg/mL) of compounds **9** and **12a**–**h** for 24 h. The expression of Hemagglutinin (HA) was analyzed by means of In Cell Western (ICW) assay (as described in the Materials and Methods section) on cell monolayers. As control of cytotoxicity, cell monolayers were also treated with the same concentrations of compounds **9** and **12a**–**h** and stained with a Cell tag (as described in the Materials and Methods section). The supernatants of the infected A549 cells were recovered and used to newly infect a fresh monolayer of MDCK (Madin-Darby canine epithelial kidney) cells, in order to evaluate whether viral particles released from the infected cells were still infective. Table 3 shows the values of IC50, CC50, and relative SI obtained on A549 and MDCK cells. Compound **12h** was the most effective against viral replication in both cell lines (IC50 range 70–73 μg/mL) with the highest SI.

**Figure 5:**
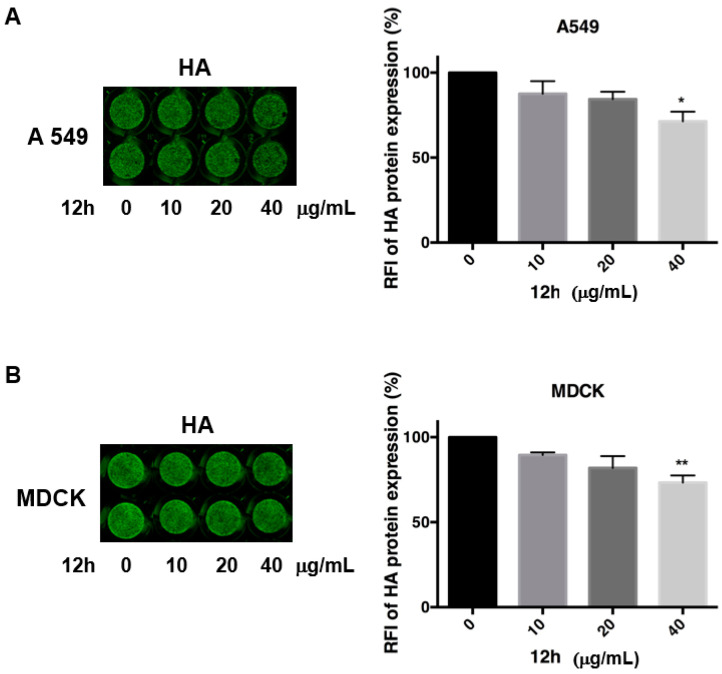
The expression of Hemagglutinin (HA) by means of In Cell Western (ICW) assay in the presence of most active compound **12h**; panel (**A**): A549 cells were infected with PR8 and treated or not with different concentrations (0–40 μg/mL) of compound **12h**. After 24 infection, cells were fixed and stained for HA protein, as described in the Materials and Methods section; panel (**B**): Supernatants were recovered and used to infect a fresh monolayer of MDCK (Madin-Darby canine epithelial kidney) cells. The expression of viral HA was analyzed by ICW assay, using LI-COR Image Studio Software. The percentage of relative fluorescence intensity (RFI) was calculated in comparison to untreated infected cells (considered 100%). Values are the mean ± S.D. of two replicates of one experiment of two performed (n = 2). Statistical significance of the data vs untreated infected cells was defined as * *p* < 0.05 and ** *p* < 0.001.

As an example, **12h** significantly reduced the HA protein expression on A549 cells (Figure 5, panel a). The released viral particles in the supernatants of A549 cells were then used to infect new monolayers of MDCK cells. After 24 h infection, the ICW assay confirmed a dose-dependent reduction of HA protein expression on MDCK cell monolayers (Figure 5, panel b), suggesting the occurrence of a block in the release of viral particles from the infected cells.

**Figure 6:**
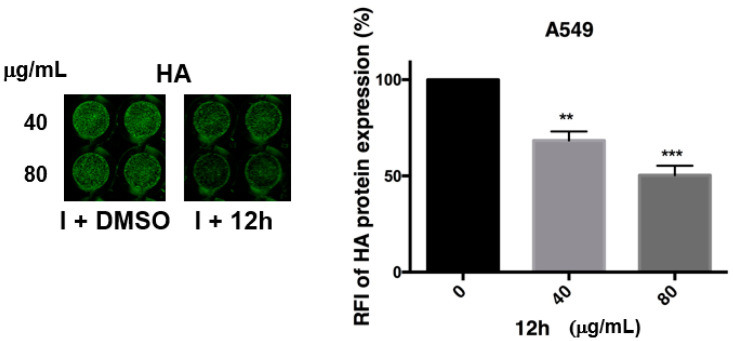
The expression of Hemagglutinin (HA) by means of ICW assay at late steps of viral replication. A549 cells were infected with PR8 and treated or not with 40 or 80 μg/mL of compound **12h**. After 24 infection, cells were fixed and stained for HA protein, as described in the Materials and Methods section. The expression of viral HA was analyzed by ICW assay, using LI-COR Image Studio Software. The percentage of relative fluorescence intensity (RFI) was calculated in comparison to untreated infected cells (considered 100%). Values are the mean ± S.D. of two experiments, each performed in duplicate (n = 4). Statistical significance of the data vs untreated infected cells was defined as ** *p* < 0.001 and *** *p* < 0.0001.

To evaluate whether the compound **12h** was able to impair the cell-to-cell virus spread, higher concentrations (40 and 80 mg/mL) of **12h** were added to A549 cell monolayers after viral challenge, and HA protein expression was analyzed directly on these monolayers after 24 h. The ICW assay showed a significant reduction of relative fluorescence intensity of HA protein (~50% inhibition with 80 mg/mL). Furthermore, the reduction of foci of infection in cell monolayers treated with the compound **12h** compared to DMSO-treated cells [48], suggested a block in the release of viral particles probably due to an interference with the viral NA (Figure 6).

## 3. Conclusions

Laccase was able to activate 1,4-dioxane as co-oxidant in the selective synthesis of belladine *N*-oxides **9** and **12a**–**h**, as confirmed by the EPR detection of the corresponding DMPO/**III** adduct. Other organic solvents were not effective in the transformation, highlighting the specific role of the formation of **III** in the oxygen atom transfer process. This reaction is an alternative to the widespread reported laccase/mediator procedure for the oxidation of amines [9,49,50]. Irrespective of the experimental conditions, the oxidation proceeded from good to high yield, showing high functional-group compatibility and chemo-selectivity avoiding the undesired formation of reactive quinone species and oligomeric products [51,52,53]. In addition, an appreciable stereoselectivity was observed, probably due to partial inhibition of the pyramidal inversion at the nitrogen center as a consequence of steric hindrance of the substituents. Compounds **12a**–**c** and 12h were the most active derivatives against influenza A virus. The highest values of IC_50_ and SI were observed in the case of **12h**, which is characterized by three carbon atoms in the side-chain and only one hydroxy moiety on the aromatic rings. As a general trend, the presence of at least one hydroxy moiety on the aromatic rings and two or three carbon atoms in the side-chain were required to obtain significant antiviral activity. Finally, the presence of an electron-withdrawing substituent on the aromatic ring (**12d**) deprived the molecule of antiviral activity.

## 4. Materials and Methods

### 4.1. Materials

Reagents and laccase from *Trametes versicolor* were obtained from commercial suppliers (Sigma-Aldrich Srl, Milan, Italy).

### 4.2. Enzyme Activity Assay

The enzyme activity was assayed by using 2,2′-azino-bis(3-ethylbenzothiazoline-6-sulfonic acid)diammonium salt (ABTS) procedure. ABTS (5.0 mM), sodium acetate buffer (2.0 mL, pH 5.0), and the enzyme solution (200 µL) were used as a standard solution. The formation of the cation radical was detected by measuring the increase of absorbance at 420 nm (ε_420_ = 36,000 M^−1^ cm^−1^). One unit of laccase activity has been defined as the amount of enzyme that catalyzed the oxidation of 1.0 µmol of ABTS in a 200 µL reaction mixture at 25 °C during 1.0 min.

### 4.3. EPR Analysis

The reaction solution was prepared adding **7b** and **11h** (40 mM), DMPO (60 mM), and laccase (0.12 mM) in 1,4-dioxane/sodium acetate buffer (9:1 ratio). To perform the EPR experiments, capillaries of 1.2 mm diameter were filled in and inserted in a quartz tube of 3 × 3.5 I.D. × O.D. CW (continuous wave) X-band (9 GHz). Experimental condition: 9.86 GHz 123 microwave frequency, 0.1 mT modulation amplitude, and 0.2 mW microwave power. EPR spectra were recorded at room temperature with a Bruker E580 Elexsys Series, using the Bruker ER4122 SHQE cavity. A simulation was carried out with the Easyspin simulation program 5.2.28 version, using the “garlic function”.

### 4.4. X-Ray Crystallography Data for Compound ***12b***

Compound **12b** was crystallized in an NMR tube, adding 5 mg of the compound in 300µL of deuterated methanol (CD_3_OD). Compound **12b** was completely dissolved heating the system, and the solution was slowly cooled overnight. A single crystal of **12b** was submitted to X-ray data collection on an Oxford-Diffraction Xcalibur Sapphire 3 diffractometer with a graphite monochromated Mo-Kα radiation (λ = 0.71073 Å) at 293 K. The structure was solved by direct methods implemented in the SHELXS program (Version 2013/1) [54]. The refinement was carried out by full-matrix anisotropic least-squares on F^2^ for all reflections for non-H atoms by means of the SHELXL program [55]. The structure crystallizes in the monoclinic crystal system, space group C2/*c*. Crystallographic data have been deposited with the Cambridge Crystallographic Data Centre as supplementary publication no. CCDC 2,045,206. Copies of the data can be obtained, free of charge, on application to CCDC, 12 Union Road, Cambridge CB2 1EZ, UK; (fax: +44-(0)-1223-336-033; or e-mail: deposit@ccdc.cam.ac.uk).

### 4.5. Procedure for the Synthesis of Amine N-Oxides ***9*** and ***12a-h***

Compounds **7b** and **11a**–**h** (1.0 eq., 0.1 mmol) were dissolved in a solvent mixture of 1,4-dioxane (2.25 mL) and sodium acetate buffer 0.1 M, pH = 5.6 (0.25 mL). Laccase (100 U·mmol^−1^) was added and the mixture was gently stirred at 25 °C under O_2_ atmosphere (balloon) for 3 h. After this period, the mixture was filtered and the solvent was evaporated under reduced pressure. The crude mixture was purified by silica gel column chromatography (ethyl acetate/methanol 7:1) to afford the desired products **9** and **12a**–**h**. Chemical reactions were monitored using thin-layer chromatography on precoated aluminum silica gel Merck 60 F254 plates and a UV lamp was used for visualization. Merck silica gel 60 (230–400 mesh) was used for chromatography. All products were dried in a high vacuum (10–3 mbar). ^1^H NMR and ^13^C-NMR and, DEPT−135 NMR were recorded on a Bruker Avance DRX400 (400 MHz/100 MHz) spectrometer. Chemical shifts for protons are reported in parts per million (δ scale) and internally referenced to the CD_3_OD and DMSO-*d_6_* signal at δ 3.33 ppm and 2.50 ppm respectively. Coupling constants (*J*) are reported in Hz. Multiplicities are reported in the conventional form: s = singlet, d = doublet, t = triplet, td = triplet of doublets, q = quartet, ABq = AB quartet, m = multiplet, br = broad. Mass spectra (MS) data were obtained using an Agilent 1100 LC/MSD VL system (G1946C) with a 0.4 mL/min flow rate using a binary solvent system of 95:5/methyl alcohol:water. Optical rotations were recorded on a JASCO P−1000 series at 589 nm and reported as follows: ${\left[\alpha \right]}_{D}^{25}$ ± value (concentration in g/100 mL, solvent).

#### 4.5.1. N-(2-Bromo-5-Hydroxy-4-Methoxybenzyl)-2-(4-Hydroxyphenyl)-N-Methylethan-1-Amine Oxide (**9**)

^1^H-NMR (400 MHz, MeOD): 7.30 (s, 1H, Ar*H*), 7.18 (s, 1H, Ar*H*), 7.12 (d, 2H, Ar*H*, *J* = 8.4 Hz), 6.75–6.72 (m, 2H, Ar*H*), 4.65–4.48 (ABq, 2H, ArC*H_2_*N-, *J*_AB_ = 12.8 Hz), 3.88 (s, 3*H*, -OC*H_3_*), 3.63–3.52 (m, 1H, -NC*H_2_*CH_2_Ar), 3.47–3.40 (td, 1H, -NC*H_2_*CH_2_Ar, *J* = 12.0, 4.4 Hz), 3.24–3.17 (td, 1H, -NCH_2_C*H_2_*Ar, *J* = 12.4, 4.4 Hz), 3.10–3.03 (m, 4H, -NCH_2_C*H_2_*Ar, -CH_3_) ppm.^13^C-NMR (100 MHz, MeOD): 156.0, 149.9, 145.9, 129.6, 127.4, 121.3, 120.9, 115.4, 115.3, 115.1, 71.7, 69.9, 55.2, 52.4, 28.0 ppm. DEPT-135-NMR (100 MHz, MeOD): 129.6 (-*C*H), 120.9 (-*C*H), 115.3 (-*C*H), 115.1 (-*C*H), 71.7 (-*C*H_2_), 69.9 (-*C*H_2_), 55.2 (-*C*H_3_), 52.4 (-*C*H_3_), 28.0 (-*C*H_2_) ppm. Non-racemic mixture ${\left[\alpha \right]}_{D}^{25}$ = +12.8 (c 1.00, DMSO). MS (ESI) m/z: (C_17_H_20_BrNO_4_)^−^: 380.08.

#### 4.5.2. N-(3-Hydroxy-4-Methoxybenzyl)-2-(4-Hydroxyphenyl)-N-Methylethan-1-Amine Oxide (**12a**)

^1^H-NMR (400 MHz, MeOD): 7.10–7.06 (m, 3H, Ar*H*), 7.03–6.96 (m, 2H, Ar*H*), 6.75–6.71 (m, 2H, Ar*H*), 4.37–4.28 (ABq, 2H, ArC*H_2_*N-, *J*_AB_ = 12.4 Hz), 3.88 (s, 3H, -OC*H_3_*), 3.41–3.16 (m, 3H, -NC*H_2_*CH_2_Ar, -NCH_2_C*H_2_*Ar) 3.09–3.02 (m, 4H, -NCH_2_C*H_2_*Ar, -CH_3_) ppm.^13^C-NMR (100 MHz, MeOD): 156.0, 149.0, 146.2, 129.5, 127.6, 123.9, 122.5, 118.9, 115.1, 110.9, 73.0, 68.9, 54.9, 53.2, 28.0 ppm. DEPT-135-NMR (100 MHz, MeOD): 129.5 (-*C*H), 123.9 (-*C*H), 118.9 (-*C*H), 115.1 (-*C*H), 110.9 (-*C*H), 73.0 (-*C*H_2_), 68.9 (-*C*H_2_), 54.9 (-*C*H_3_), 53.1 (-*C*H_3_), 28.0 (-*C*H_2_) ppm. Non-racemic mixture ${\left[\alpha \right]}_{D}^{25}\;$= +17.4 (c 1.00, DMSO). MS (ESI) m/z: (C_17_H_21_NO_4_)^−^: 302.09.

#### 4.5.3. N-(3-hydroxybenzyl)-2-(4-hydroxyphenyl)-N-methylethan-1-amine oxide (**12b**)

^1^H-NMR (400 MHz, MeOD): 7.26 (t, 1H, Ar*H*, *J* = 8.0 Hz), 7.11 (d, 2H, Ar*H*, *J* = 8.4 Hz), 7.05–7.03 (m, 2H, Ar*H*), 6.90–6.88 (dd, 1H, Ar*H*, *J* = 8.4, 6.8 Hz), 6.75 (d, 2H, Ar*H*, *J* = 8.8 Hz), 4.42–4.33 (ABq, 2H, ArC*H_2_*N-, *J*_AB_ = 12.8 Hz), 3.45–3.28 (m, 2H, -NC*H_2_*CH_2_Ar), 3.24–3.17 (td, 1H, -NCH_2_C*H_2_*Ar, *J* = 12.4, 4.4 Hz), 3.11–3.08 (m, 4H, -NCH_2_C*H_2_*Ar, -CH_3_) ppm.^13^C-NMR (100 MHz, MeOD): 157.3, 156.0, 131.2, 129.5, 129.2, 127.5, 123.2, 119.0, 116.4, 115.1, 73.1, 69.3, 53.4, 28.1 ppm. DEPT-135-NMR (100 MHz, MeOD): 129.5 (-*C*H), 129.2 (-*C*H), 123.2 (-*C*H), 119.0 (-*C*H), 116.4 (-*C*H), 115.1 (-*C*H), 73.0 (-*C*H_2_), 69.3 (-*C*H_2_), 53.3 (-*C*H_3_), 28.0 (-*C*H_2_) ppm. Non-racemic mixture ${\left[\alpha \right]}_{D}^{25}\;$= +11.5 (c 1.00, DMSO). MS (ESI) m/z: (C_16_H_19_NO_3_)^−^: 272.19.

#### 4.5.4. N-(4-Hydroxybenzyl)-2-(4-Hydroxyphenyl)-N-Methylethan-1-Amine Oxide (**12c**)

^1^H-NMR (400 MHz, MeOD): 7.41–7.37 (m, 2H, Ar*H*), 7.09 (d, 2H, Ar*H*, *J* = 8.4 Hz), 6.85–6.82 (m, 2H, Ar*H*), 6.74–6.72 (m, 2H, Ar*H*), 4.39–4.30 (ABq, 2H, ArC*H_2_*N-, *J*_AB_ = 12.8 Hz), 3.41–3.15 (m, 3H, -NC*H_2_*CH_2_Ar, NCH_2_C*H_2_*Ar), 3.09–3.00 (m, 4H, -NCH_2_C*H_2_*Ar, -CH_3_) ppm.^13^C-NMR (100 MHz, MeOD): 158.8, 156.0, 133.6, 129.5, 127.6, 120.5, 115.1, 114.9, 72.8, 68.8, 52.9, 28.0 ppm. DEPT-135-NMR (100 MHz, MeOD): 133.6 (-*C*H), 129.5 (-*C*H), 115.1 (-*C*H), 114.9 (-*C*H), 72.7 (-*C*H_2_), 68.8 (-*C*H_2_), 52.8 (-*C*H_3_), 28.0 (-*C*H_2_) ppm. Non-racemic mixture ${\left[\alpha \right]}_{D}^{25}$ = +21.5 (c 1.00, DMSO). MS (ESI) m/z: (C_16_H_19_NO_3_)^−^: 272.21.

#### 4.5.5. N-(3-Hydroxy-4-Nitrobenzyl)-2-(4-Hydroxyphenyl)-N-Methylethan-1-Amine Oxide (**12d**)

^1^H-NMR (400 MHz, MeOD): 8.10 (d, 1H, Ar*H*, *J* = 8.8 Hz), 7.44 (d, 1H, Ar*H*, *J* = 1,6 Hz), 7.24–7.22 (dd, 1H, Ar*H*, *J* = 8.8, 1,6 Hz), 7.11 (d, 2H, Ar*H*, *J* = 8.4 Hz), 6.75–6.72 (m, 2H, ArH), 4.50–4.42 (ABq, 2H, *J*_AB_ = ArC*H_2_*N-, 12.8 Hz), 3.55–3.48 (td, 1H, -NC*H_2_*CH_2_Ar, *J* = 12.0, 5.6 Hz), 3.40–3.31 (td, 1H, -NC*H_2_*CH_2_Ar, *J* = 12.0, 4.8 Hz), 3.23–3.16 (td, 1H, -NCH_2_C*H_2_*Ar, *J* = 12.0, 4.8 Hz), 3.13–3.08 (m, 4H, -NCH_2_C*H_2_*Ar, -CH_3_) ppm.^13^C-NMR (100 MHz, MeOD): 155.5, 153.3, 138.1, 134.9, 129.0, 126.9, 124.0, 123.7, 122.7, 114.6, 70.6, 69.9, 53.2, 27.6 ppm. DEPT-135-NMR (100 MHz, MeOD): 129.5 (-*C*H), 124.6 (-*C*H), 124.2 (-*C*H), 123.2 (-*C*H) 115.1 (-*C*H), 71.2 (-*C*H_2_), 70.5 (-*C*H_2_), 53.5 (-*C*H_3_), 28.1 (-*C*H_2_) ppm. Non-racemic mixture ${\left[\alpha \right]}_{D}^{25}$ = +6.4 (c 1.00, DMSO). MS (ESI) m/z: (C_16_H_18_N_2_O_5_)^−^: 317.09.

#### 4.5.6. N-Benzyl-2-(4-Hydroxyphenyl)-N-Methylethan-1-Amine Oxide (**12e**)

^1^H-NMR (400 MHz, MeOD): 7.61–7.59 (m, 2H, Ar*H*,), 7.49–7.42 (m, 3H, Ar*H*), 7.10–7.07 (m, 2H, Ar*H*), 6.75–6.71 (m, 2H, Ar*H*) 4.51–4.43 (ABq, 2H, ArC*H_2_*N-, *J*_AB_ = 12.8 Hz), 3.49–3.41 (td, 1H, -NC*H_2_*CH_2_Ar, *J* = 12.0, 5.6 Hz), 3.38–3.30 (m, 1H, -NC*H_2_*CH_2_Ar), 3.24–3.16 (td, 1H, -NCH_2_C*H_2_*Ar, *J* = 12.0, 4.8 Hz), 3.12–3.04 (m, 4H, -NCH_2_C*H_2_*Ar, -C*H_3_*) ppm.^13^C-NMR (100 MHz, MeOD): 156.0, 132.4, 129.8, 129.6, 129.5, 128.2, 127.4, 115.2, 72.7, 69.4, 53.0, 28.0 ppm. DEPT-135-NMR (100 MHz, MeOD): 132.4 (-*C*H), 129.5 (-*C*H), 129.5 (-*C*H), 128.2 (-*C*H) 115.1 (-*C*H), 72.7 (-*C*H_2_), 69.4 (-*C*H_2_), 53.0 (-*C*H_3_), 28.0 (-*C*H_2_) ppm. Non-racemic mixture ${\left[\alpha \right]}_{D}^{25}$ = +18.6 (c 1.00, DMSO). MS (ESI) m/z: (C_16_H_19_NO_2_)^−^: 256.27.

#### 4.5.7. 1-(3-hydroxyphenyl)-N,N-dimethylmethanamine oxide (**12f**)

^1^H-NMR (400 MHz, MeOD): 7.28–7.24 (m, 1H, Ar*H*), 7.02–7.00 (m, 2H, Ar*H*), 6.91–6.88 (m, 1H, Ar*H*), 4.35 (s, 2H, ArC*H_2_*N-), 3.12 (s, 6H, -N(C*H*_3_)_2_).^13^C-NMR (100 MHz, MeOD): 157.4, 131.3, 129.2, 123.1, 119.0, 116.4, 74.2, 56.5 ppm. DEPT-135-NMR (100 MHz, MeOD): 129.2 (-*C*H), 123.1 (-*C*H), 119.0 (-*C*H), 116.4 (-*C*H), 74.1 (-*C*H_2_), 56.5 (-*C*H_3_) ppm. MS (ESI) m/z: (C_9_H_13_NO_2_)^−^: 166.12.

#### 4.5.8. N-(3-hydroxybenzyl)-1-(4-hydroxyphenyl)-N-methylmethanamine oxide (**12g**)

^1^H-NMR (400 MHz, MeOD): 7.42–7.40 (m, 2H, Ar*H*), 7.25 (t, 1H, Ar*H*, *J* = 7.6 Hz), 6.89–6.82 (m, 5H, Ar*H*), 4.32–4.30 (m, 4H, ArC*H_2_*NC*H_2_*Ar), 2.77 (s,3H, -C*H_3_*).^13^C-NMR (100 MHz, MeOD): 158.7, 157.2, 139.4, 133.9, 131.2, 123.5, 120.5, 119.3, 116.2, 114.8, 72.8, 72.5, 51.3 ppm. DEPT-135-NMR (100 MHz, MeOD): 134.9 (-*C*H), 131.5 (-*C*H), 120.5 (-*C*H), 119.3 (-*C*H), 116.3 (-*C*H), 114.8 (-*C*H), 72.5 (-*C*H_2_), 72.3 (-*C*H_2_), 51.3 (-*C*H_3_) ppm. Non-racemic mixture ${\left[\alpha \right]}_{D}^{25}$ = +8.9 (c 1.00, DMSO). MS (ESI) m/z: (C_15_H_17_NO_3_)^−^: 258.16.

#### 4.5.9. N-(3-Hydroxybenzyl)-N-Methyl-3-Phenylpropan-1-Amine Oxide (**12h**)

^1^H-NMR (400 MHz, MeOD): 7.31–7.18 (m, 6H, Ar*H*), 7.00 (t, 1H, Ar*H*, *J* = 2.0 Hz), 6.95–6.93 (m, 1H, Ar*H*), 6.89–6.87 (m, 1H, Ar*H*), 4.38–4.29 (ABq, 2H, ArC*H_2_*N-, *J*_AB_ = 12.4 Hz), 3.32–3.20 (m, 2H, -NC*H_2_*CH_2_CH_2_Ar), 3.00 (s, 3H, -CH_3_), 2.69 (t, 2H, -NCH_2_CH_2_C*H_2_*Ar, *J* = 7.6 Hz), 2.34–2.16 (m, 2H, -NCH_2_C*H_2_*CH_2_Ar) ppm. ^13^C-NMR (100 MHz, MeOD): 157.3, 140.5, 130.8, 129.2, 128.2, 128.0, 125.9, 123.1, 119.1, 116.4, 72.6, 67.4, 53.2, 32.2, 24.4 ppm. DEPT-135-NMR (100 MHz, MeOD): 129.2 (-*C*H), 128.1 (-*C*H), 128.0 (-*C*H), 125.9 (-*C*H), 123.1 (-*C*H) 119.1, (-*C*H), 116.4 (-*C*H), 72.6 (-*C*H_2_-), 67.4 (-*C*H_2_), 53.1 (-*C*H_3_), 32.2 (-*C*H_2_), 24.4 (-*C*H_2_) ppm. Non-racemic mixture ${\left[\alpha \right]}_{D}^{25}$ = –7.1 (c 1.00, DMSO). MS (ESI) m/z: (C_17_H_21_NO_2_)^−^: 270.19.

### 4.6. Cell Cultures

A 549 (human lung epithelial carcinoma) and MDCK (Madin-Darby canine epithelial kidney) cell lines were grown in RPMI 1640 medium supplemented with 10% fetal calf serum (FCS); glutamine 0.3 mg/mL; penicillin 100 U/mL and streptomycin 100 mg/mL. Cell viability was estimated by trypan blue (0.02%) exclusion. All reagents were purchased from Invitrogen (Milan, Italy).

### 4.7. Virus Production and Infection

Influenza virus A/Puerto Rico/8/34 H1N1 (PR8 virus) was grown in the allantoic cavities of 10-day-old embryonated chicken eggs. After 48 h at 37 °C, the allantoic fluid was harvested, centrifuged at 5000 rpm for 30 min to remove cellular debris, and stored at −80 °C. Virus titration was performed by Tissue Culture Infectious Dose 50% (TCID50%).

Confluent monolayers of A549 epithelial cells were challenged for 1 h at 37 °C with PR8 at a multiplicity of infection (m.o.i.) of 0.001 (TCID50%/cell) incubated for 1 h at 37 °C, washed with PBS, and then incubated with medium supplemented with 2% FCS. Mock infection was performed with the same dilution of allantoic fluid from uninfected eggs. [56].

### 4.8. In Cell Western (ICW) Assay

The ICW assay was performed using the Odyssey Imaging System (LI-COR, Lincoln, NE, USA) as previously described [47]. Briefly, A549 or MDCK cells grown in 96-well plates (2 × 10^4^ cells/well), either infected or mock-infected (Ctr) with PR8, were fixed with 4% formaldehyde, washed, permeabilized with 0.1% Triton X-100 and incubated with PBS containing Odyssey Blocking buffer (LI-COR Biosciences, Lincoln, NE, USA). The cells were then stained at 4 °C overnight with mouse anti HA (Santa Cruz Biotechnology, Santa Cruz, CA, USA) together with Cell Tag (LI-COR Biosciences, Lincoln, NE, USA) in PBS containing 5% Odyssey Blocking Buffer. Cells were then washed and stained with a mixture of fluorochrome-conjugated secondary antibodies (fluorescence emission at 800 nm) (LI-COR Biosciences, Lincoln, NE, USA) properly diluted in Odyssey blocking buffer and fluorochrome-conjugated Cell Tag (fluorescence emission at 700 nm), for 1 h at room temperature. Cell Tag was used as control of the integrity of the cell monolayer. Subsequently, three washes with PBS plus 0.1% Tween 20 were performed and plates were analyzed by the Odyssey infrared imaging system (LI-COR). Integrated intensities of fluorescence were determined by the LI-COR Image Studio software and the relative fluorescence intensity (RFI) was expressed as a percentage compared to untreated infected cells (100%). The concentration of compounds causing a 50% reduction of viral infection (IC_50_) and the 50% cytotoxic concentration (CC_50_), defined as the compound concentration required to reduce cell viability by 50%, were calculated by regression analysis, considering untreated infected cells as control (100%). The Selectivity Index (SI) of each compound was calculated as the ratio CC_50_/IC_50_.

## Figures and Tables

**Table 1:** Laccase-catalyzed 1,4-dioxane-mediated synthesis of belladine *N*-oxide derivative **9** starting from **7b**. ^1^

Entry	pH	Solvent	Solvent/Buffer Ratio	Conversion (%)	Product(s)	Yield ^5^ (%)
1	4.5	1,4-dioxane	1:4	81	**8**	76 ^2^
2	4.5	1,4-dioxane	1:4	45	**8**(**9**)	34(7) ^3^
3	5.6	1,4-dioxane	1:4	50	**8**(**9**)	19(28) ^3^
4	5.6	1,4-dioxane	10:1	60	**8**(**9**){**10**}	5(47){5} ^3^
5	5.6	1,4-dioxane	10:1	71	**8**(**9**){**10**}	2(56){8} ^3, 4^
6	5.6	THF	10:1	16	(**9**)	(2) ^3^
7	5.6	CH_3_CN	10:1	5	**8**	2 ^3^

^1^ All the reactions were performed using 0.1 mmol of the substrate in the appropriate solvent/buffer ratio (2.50 mL of total volume) and sodium-acetate buffer (0.1 M) at appropriate pH under O_2_ atmosphere at 25 °C for 3 h. The experiments were conducted in triplicate. ^2^ Reaction performed in the presence of 1000 U·mmol^−1^ of laccase and TEMPO (0.06 mmol). ^3^ Reaction performed in the presence of 100 U·mmol^−1^ of laccase alone. ^4^ Reaction performed in the presence of 1,4-dioxane deprived of butyl hydroxytoluene (BHT). ^5^ The yield was calculated on the basis of starting mmol of the substrate.

**Table 2:** Synthesis of belladine *N*-oxide derivatives **9** and **12a**–**h ^1^^.^**

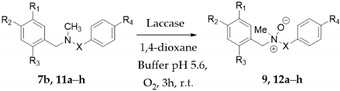									
Entry	Compd	X	R_1_	R_2_	R_3_	R_4_	Product	Yield [%] ^2^	${\left[\alpha \right]}_{D}^{25}$ ^3^
1	**7b**	-(CH_2_)_2_-	-OH	-OCH_3_	-Br	-OH	**9**	56	+12.8
2	**11a**	-(CH_2_)_2_-	-OH	-OCH_3_	-H	-OH	**12a**	55	+17.4
3	**11b**	-(CH_2_)_2_-	-OH	-H	-H	-OH	**12b**	53	+11.5
4	**11c**	-(CH_2_)_2_-	-H	-OH	-H	-OH	**12c**	52	+21.5
5	**11d**	-(CH_2_)_2_-	-OH	-NO_2_	-H	-OH	**12d**	70	+6.4
6	**11e**	-(CH_2_)_2_-	-H	-H	-H	-OH	**12e**	71	+18.6
7	**11f**	-CH_3_	-OH	-H	-H	none	**12f**	67	-
8	**11g**	-(CH_2_)-	-OH	-H	-H	-OH	**12g**	55	+8.9
9	**11h**	-(CH_2_)_3_-	-OH	-H	-H	-H	**12h**	78	−7.1

^1^ The reactions were performed using 100 U·mmol^−1^ of laccase for 0.1 mmol of the substrate in a solvent mixture of 1,4-dioxane (2.25 mL) and sodium-acetate buffer 0.1 M pH 5.6 (0.25 mL) under O_2_ atmosphere at 25 °C for 3. All the reactions were conducted in triplicate. Reactions were performed in the presence of 1,4-dioxane deprived by distillation of the commercial additive butyl hydroxytoluene (BHT). ^2^ The yield was calculated on the basis of starting mmol of the substrate. ^3^ Optical rotations were recorded on a JASCO P−1000 series at 589 nm.

**Table 3:** IC50, CC50 values and selective index (SI) of belladine *N*-oxides derivatives **9** and **12a**–**h** ^1^

Entry	Compd	A549			MDCK		
		IC50	CC50	SI	IC50	CC50	SI
1	**9**	376.88 ± 11.4	210.65 ± 8.1	0.56	86.84 ± 5.1	205.90 ± 7.9	2.37
2	**12a**	78.76 ± 4.3	200 ± 7.1	2.5	137.23 ± 5.9	137.48 ± 5.8	1.00
3	**12b**	83.23 ± 5.1	149.31 ± 6.2	1.79	138.28 ± 6.1	129.18 ± 6.0	0.93
4	**12c**	80.20 ± 4.9	83.79 ± 5.0	1.04	84.73 ± 4.8	113.86 ± 5.2	1.34
5	**12d**	1243.97 ± 30.2	112.69 ± 6.1	0.09	70.15 ± 4.0	100.51 ± 4.9	1.43
6	**12e**	ND ^2^	ND ^2^	ND ^2^	133.32 ± 5.8	108.61 ± 5.1	0.81
7	**12f**	ND ^2^	ND ^2^	ND ^2^	ND ^2^	ND ^2^	ND ^2^
8	**12g**	ND ^2^	ND ^2^	ND ^2^	ND ^2^	ND ^2^	ND ^2^
9	**12h**	70.40 ± 3.1	287.28 ± 8.3	4.0	73.20 ± 3.3	220.74 ± 8.9	3.01

^1^ IC_50:_ the concentration of compound causing 50% reduction of viral infection. CC_50_: the concentration of compound required to reduce cell viability by 50%. IC_50_ and CC_50_ are expressed in micromolar units as mean ± SD. All experiments were conducted in triplicate. The Selectivity Index (SI) of each compound was calculated as the ratio CC_50_/IC_50_. ^2^ ND = Not Determined.

## Data Availability

The data presented in this study are available in Supplementary Materials.

## Supplementary Materials

Supplementary Materials can be found at https://www.mdpi.com/1422-0067/22/3/1337/s1.
