# Supplementary material for: Laccase-Catalyzed 1,4-Dioxane-Mediated Synthesis of Belladine N-Oxides with Anti-Influenza A Virus Activity
Source: Int J Mol Sci. 2021 Jan 29;22(3):1337. doi: 10.3390/ijms22031337 (PMC7866262; doi:10.3390/ijms22031337)
Supplement: Supplementary file 1 [file ijms-22-01337-s001.pdf]

Supplementary Materials for

Laccase-catalyzed 1,4-dioxane-mediated synthesis of belladine *N*-oxides with anti-influenza A virus activity.

Claudio Zippilli,[a] Lorenzo Botta,[a] Bruno Mattia Bizzarri,[a] Lucia Nencioni,[b,*] Marta De Angelis,[b] Virginia Protto,[b] Gianluca Giorgi,[c] Maria Camilla Baratto,[c] Rebecca Pogni,[c] Raffaele Saladino[a,*].

[a]*Department of Biological and Ecological Sciences (DEB), University of Tuscia, Via S. Camillo de Lellis snc, 01100 Viterbo, Italy.*

[b]*Department of public health and infectious diseases, laboratory affiliated to Istituto Pasteur Italia, Fondazione Cenci Bolognetti, Sapienza University of Rome, 00185 Rome, Italy.*

[c]*Department of biotechnology, chemistry and pharmacy, University of Siena, Via Aldo Moro 2, 53100 Siena, Italy.*

Content

SM #1. General procedures *1*

SM #2. Synthesis of compounds 7b and 11a-h

- *SM #2.1. Synthesis of compounds **7b** and **11a-e*** 2
- *SM #2.2. Synthesis of compound **11f*** 4
- *SM #2.3. Synthesis of compound **11g*** 4
- *SM #2.4. Synthesis of compound **11h*** 5

SM #3. NMR spectra

- *SM #3.1. NMR spectra of intermediates **iii a-h*** 6
- *SM #3.2. NMR spectra of compounds **7b** and **11a-h*** 14
- *SM #3.3. NMR spectra of compounds **9** and **12a-h*** 23
- *SM #3.4. NMR spectra of compound **10*** 36

SM #1. General procedures

Reagents and solvents were obtained from commercial suppliers (Sigma-Aldrich Srl, Milan, Italy). Reactions were monitored using thin layer chromatography on precoated aluminium silica gel Merck 60 F254 plates and an UV lamp (λmax = 254 nm) was used for visualization. Merck silica gel 60 (230-400 mesh) was used for flash chromatography with the indicated solvent system. All products were dried in high vacuum (10-3 mbar). ^{1}H NMR and ^{13}C-NMR and, DEPT-135 NMR were recorded on a Bruker Avance DRX400 (400 MHz/100 MHz) spectrometer. Chemical shifts for protons are reported in parts per million (δ scale) and internally referenced to the CD_3OD and DMSO-d_6 signal at δ 3.33 ppm and 2.50 ppm respectively. Coupling constants (*J*) are reported in Hz. Multiplicities are reported in the conventional form: s = singlet, d = doublet, t = triplet, td= triplet of doublets, q = quartet, ABq = AB quartet, m = multiplet, br = broad.

SM #2. Synthesis of compounds 7b and 11a-h

*SM #2.1. Synthesis of compounds **7b** and **11a-e***

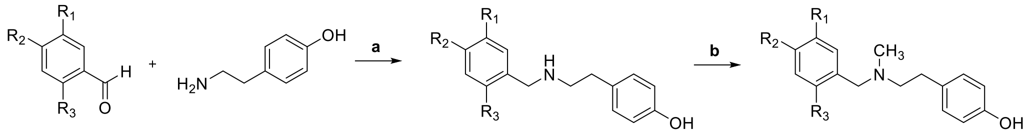

ia-f **iia** **iiia-f** **7b, 11a-e**

a: $NaBH_4$, MeOH, r.t. (90-96%); **b**: H_2CO, $NaBH_4$, MeOH, r.t. (93-98%).

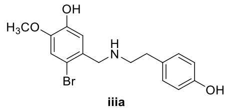

iiia

4-bromo-5-(((4-hydroxyphenethyl)amino)methyl)-2-methoxyphenol ***iiia***: 2-bromo-5-hydroxy-4-methoxybenzaldehyde **ia** (1.0 eq., 1.0 mmol) and tyramine **iia** (1.1 eq., 1.1 mmol) were dissolved in methanol (2.0 mL) and the solution was stirred for 4h at room temperature. Then $NaBH_4$ (1.1 eq., 1.1 mmol) was added at 0°C and the mixture was stirred for 5h at room temperature. After this period the mixture was filtered over Celite® and the solvent was evaporated under reduced pressure. The crude mixture was purified by silica gel gradient column chromatography (DCM/MeOH/NH_4OH 7:1:0.1) to afford **iiia** as a white powder (96%). **^{1}H-NMR** (400 MHz, MeOD): 7.05 (s, 1H, Ar*H*), 7.00 (d, 2H, Ar*H*, J= 8.4 Hz), 6.84 (s, 1H, Ar*H*), 6.71-6.69 (m, 2H, Ar*H*), 3.83 (s, 3H, -OC*H*$_3$), 3.71 (s, 2H, ArC*H*$_2$N-), 2.80-2-76 (m, 2H, -NC*H*$_2$CH$_2$Ar), 2.73-2.70 (m, 2H, -NCH$_2$C*H*$_2$Ar) ppm. **^{13}C-NMR** (100 MHz, MeOD): 155.5, 147.8, 146.0, 130.2, 129.9, 129.2, 117.1, 115.4, 114.9, 111.7, 55.3, 52.2, 49.7, 34.2 ppm.

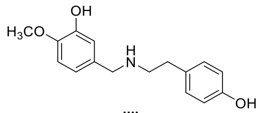

iiib

5-(((4-hydroxyphenethyl)amino)methyl)-2-methoxyphenol ***iiib:*** 3-hydroxy-4-methoxybenzaldehyde **ib** (1.0 eq., 1.0 mmol) and tyramine **iia** (1.1 eq., 1.1 mmol) were dissolved in methanol (2.0 mL) and the solution was stirred for 4h at room temperature. Then $NaBH_4$ (1.1 eq., 1.1 mmol) was added at 0°C and the mixture was stirred for 5h at room temperature. Compound **iiib** was purified by crystallization in methanol to afford a colorless crystal (90%). **^{1}H-NMR** (400 MHz, DMSO-*d*$_6$): 6.97 (d, 2H, Ar*H*, J= 8.0 Hz), 6.81 (d, 1H, Ar*H*, J= 8.0 Hz), 6.73 (s, 1H, Ar*H*), 6.65 (m, 3H, Ar*H*), 3.72 (s, 3H, -OC*H*$_3$), 3.54 (s, 2H, ArC*H*$_2$N-), 2.60-2.58 (brm , 4H, -NC*H*$_2$C*H*$_2$Ar), ppm. **^{13}C-NMR** (100 MHz, DMSO-*d*$_6$): 155.8, 146.7, 146.6, 134.0, 130.9, 129.8, 118.9, 115.8, 115.4, 112.4, 56.1, 53.0, 51.1, 35.4 ppm.

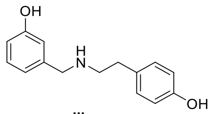

iiic

3-(((4-hydroxyphenethyl)amino)methyl)phenol ***iiic*** : 3-hydroxybenzaldehyde **ic** (1.0 eq., 1.0 mmol) and tyramine **iia** (1.1 eq., 1.1 mmol) were dissolved in methanol (2.0 mL) and the solution was stirred for 4h at room temperature. Then $NaBH_4$ (1.1 eq., 1.1 mmol) was added at 0°C and the mixture was stirred for 5h at room temperature. Compound **iiic** was purified by crystallization in methanol to afford a colorless crystal (91%). **^{1}H-NMR** (400 MHz, DMSO-*d*$_6$): 7.06 (t, 1H, Ar*H*, J= 7.6), 6.98 (d, 2H, Ar*H*, J= 8.0 Hz), 6.72-6.58 (m, 5H, Ar*H*), 3.60 (s, 2H, ArC*H*$_2$N-), 2.62-2.61 (brd, 4H, -NC*H*$_2$C*H*$_2$Ar), ppm. **^{13}C-NMR** (100 MHz, DMSO-*d*$_6$): 157.7, 155.8, 142.9, 130.9, 129.8, 129.4, 118.9, 115.4, 115.1, 113.8, 53.3, 51.3, 35.4 ppm.

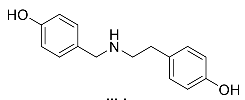

iiid

4-(2-((4-hydroxybenzyl)amino)ethyl)phenol ***iiid:*** 4-hydroxybenzaldehyde **id** (1.0 eq., 1.0 mmol) and tyramine **iia** (1.1 eq., 1.1 mmol) were dissolved in methanol (2.0 mL) and the solution was stirred for 4h at room temperature. Then $NaBH_4$ (1.1 eq., 1.1 mmol) was added at 0°C and the mixture was stirred for 5h at room temperature. After this period the mixture was filtered over Celite® and the solvent evaporated under reduced pressure. The crude mixture was purified by silica gel gradient column chromatography (DCM/MeOH/NH_4OH 7:1:0.1) to afford **iiid** as a white powder (94%). **^{1}H-NMR** (400 MHz, MeOD): 7.13-7.11 (dd, 2H, Ar*H*, J= 6.4, 2.0 Hz), 7.02-7.00 (dd, 2H, Ar*H*, J= 6.4, 2.0 Hz), 6.75-6.70 (m, 4H, Ar*H*), 3.67 (s, 2H, ArC*H*$_2$N-), 2.81-2.70 (m, 4H, -NC*H*$_2$C*H*$_2$Ar), ppm. **^{13}C-NMR** (100 MHz, MeOD): 156.5, 155.5, 130.0, 129.4, 129.3, 129.1, 114.9, 114.8, 52.3, 49.9, 34.0 ppm

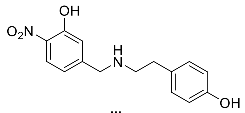

iiie

5-(((4-hydroxyphenethyl)amino)methyl)-2-nitrophenol ***iiie:*** 3-hydroxy-4-nitrobenzaldehyde **ie** (1.0 eq., 1.0 mmol) and tyramine **iia** (1.1 eq., 1.1 mmol) were dissolved in methanol (2.0 mL) and the solution was stirred for 4h at room temperature. Then $NaBH_4$ (1.1 eq., 1.1 mmol) was added at 0°C and the mixture was stirred for 5h at room temperature. Compound **iiie** was purified by crystallization in methanol to afford an orange crystal (91%). **^{1}H-NMR** (400 MHz, DMSO-*d*$_6$): 7.80 (d, 1H, Ar*H*, J= 8.4 Hz), 7.00-6.98 (m, 3H, Ar*H*), 6.78 (d, 2H, Ar*H*, J= 8.4 Hz), 6.66 (d, 1H, Ar*H*, J= 8.4 Hz), 3.70 (s, 2H, ArC*H*$_2$N-), 2.69-2.60 (m, 4H, -NC*H*$_2$C*H*$_2$Ar), ppm. **^{13}C-NMR** (100 MHz, DMSO-*d*$_6$): 155.9, 155.1, 149.0, 135.5, 130.4, 129.8, 125.7, 119.6, 117.7, 115.5, 52.3, 51.0, 35.0 ppm.

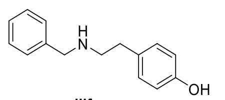

iiif

4-(2-(benzylamino)ethyl)phenol ***iiif*** : benzaldehyde **if** (1.0 eq., 1.0 mmol) and tyramine **iia** (1.1 eq., 1.1 mmol) were dissolved in methanol (2.0 mL) and the solution was stirred for 4h at room temperature. Then $NaBH_4$ (1.1 eq., 1.1 mmol) was added at 0°C and the mixture was stirred for 5h at room temperature. After this period the mixture was filtered over Celite® and evaporated under reduced pressure. The crude mixture was purified by silica gel gradient column chromatography (DCM/MeOH/NH_4OH 15:1:0.1) to afford **iiif** as a white powder (96%). ^{1}**H-NMR** (400 MHz, DMSO-d_6): 7.28-7.17 (m, 5H, Ar*H*), 6.97 (d, 2H, Ar*H*, J= 8.4 Hz), 6.65 (d, 2H, Ar*H*, J= 8.4 Hz), 3.68 (s, 2H, ArCH_2N-), 2.64-2-59 (brm, 4H, -NCH_2CH_2Ar), ppm. ^{13}**C-NMR** (100 MHz, DMSO-d_6): 155.8, 141.4, 130.9, 129.8, 128.5, 128.3, 126.9, 115.4, 53.3, 51.2, 35.4 ppm.

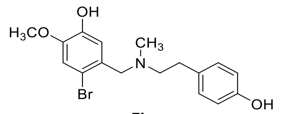

7b

4-bromo-5-(((4-hydroxyphenethyl)(methyl)amino)methyl)-2-methoxyphenol ***7b*** : compound **iiia** (1.0 eq., 1.0 mmol) and formaldehyde (1.05 eq., 1.05 mmol) were dissolved in methanol (2.0 mL) and the solution was stirred for 4h at room temperature. Then $NaBH_4$ (1.1 eq., 1.1 mmol) was added at 0°C and the mixture was stirred for 5h at room temperature. After this period the mixture was filtered over Celite® and the solvent was evaporated under reduced pressure. The crude mixture was purified by silica gel gradient column chromatography (DCM/MeOH/NH_4OH 14:1:0.1) to afford **7b** as a white powder (98%). ^{1}**H-NMR** (400 MHz, MeOD): 7.07 (s, 1H, Ar*H*), 7.01 (d, 2H, Ar*H*, J= 8.8 Hz), 6.93 (s, 1H, Ar*H*), 6.71-6.67 (m, 2H, Ar*H*), 3.83 (s, 3H, -OCH_3), 3.58 (s, 2H, ArCH_2N-), 2.76-2-72 (m, 2H, -NCH_2CH$_2$Ar), 2.65-2.60 (m, 2H, -NCH$_2$CH_2Ar), 2.29 (s, 3H, -NCH_3) ppm. ^{13}**C-NMR** (100 MHz, MeOD): 155.2, 147.7, 145.8, 130.8, 129.5, 129.1, 117.5, 115.3, 114.8, 112.8, 60.0, 59.5, 55.2, 40.9, 32.1 ppm.

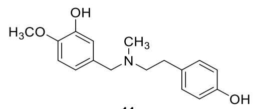

11a

5-(((4-hydroxyphenethyl)(methyl)amino)methyl)-2-methoxyphenol ***11a:*** compound **iiib** (1.0 eq., 1.0 mmol) and formaldehyde (1.05 eq., 1.05 mmol) were dissolved in methanol (2.0 mL) and the solution was stirred for 4h at room temperature. Then $NaBH_4$ (1.1 eq., 1.1 mmol) was added at 0°C and the mixture was stirred for 5h at room temperature. After this period the mixture was filtered over Celite® and the solvent was evaporated under reduced pressure. The crude mixture was purified by silica gel gradient column chromatography (DCM/MeOH/NH_4OH 16:1:0.1) to afford **11a** as a white powder (97%). ^{1}**H-NMR** (400 MHz, MeOD): 7.01-6.98 (m, 2H, Ar*H*), 6.90 (d, 1H, Ar*H*, J= 8.4 Hz), 6.82 (s, 1H, Ar*H*), 6.78-6.75 (dd, 1H, Ar*H*, J= 8.0, 2 Hz), 6.72-6.68 (m, 2H, Ar*H*), 3.86 (s, 3H, -OCH_3), 3.50 (s, 2H, ArCH_2N-), 2.76-2.72 (m, 2H, -NCH_2CH$_2$Ar), 2.60-2.56 (m, 2H, -NCH$_2$CH_2Ar), 2.29 (s, 3H, -NCH_3) ppm. ^{13}**C-NMR** (100 MHz, MeOD): 155.3, 147.1, 146.0, 130.5, 129.9, 129.1, 120.8, 116.4, 114.8, 111.0, 60.9, 58.8, 55.0, 40.7, 31.8 ppm.

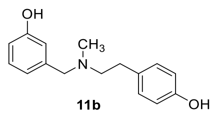

11b

3-(((4-hydroxyphenethyl)(methyl)amino)methyl)phenol ***11b:*** compound **iiic** (1.0 eq., 1.0 mmol) and formaldehyde (1.05 eq., 1.05 mmol) were dissolved in methanol (2.0 mL) and the solution was stirred for 4h at room temperature. Then $NaBH_4$ (1.1 eq., 1.1 mmol) was added at 0°C and the mixture was stirred for 5h at room temperature. After this period the mixture was filtered over Celite® and the solvent was evaporated under reduced pressure. The crude mixture was purified by silica gel gradient column chromatography (DCM/MeOH/NH_4OH 14:1:0.1) to afford **11b** as a white powder (94%). ^{1}**H-NMR** (400 MHz, MeOD): 7.15 (t, 1H, Ar*H*, J= 6.4, 1.6 Hz), 7.01-6.98 (m, 2H, Ar*H*), 6.81-6.79 (m, 2H, Ar*H*), 6.73-6.68 (m, 3H, Ar*H*), 3.53 (s, 2H, ArCH_2N-), 2.76-2.72 (m, 2H, -NCH_2CH$_2$Ar), 2.61-2.57 (m, 2H, -NCH$_2$CH_2Ar), 2.29 (s, 3H, -NCH_3) ppm. ^{13}**C-NMR** (100 MHz, MeOD): 157.1, 155.2, 138.8, 130.6, 129.2, 128.9, 120.6, 116.2, 114.9, 114.0, 61.4, 59.0, 41.0, 31.9 ppm.

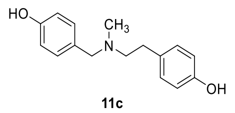

11c

4-(2-((4-hydroxybenzyl)(methyl)amino)ethyl)phenol ***11c:*** compound **iiid** (1.0 eq., 1.0 mmol) was dissolved in methanol (2.0 mL) and formaldehyde (1.05 eq., 1.05 mmol) was added to the solution. Then $NaBH_4$ (1.1 eq., 1.1 mmol) was added at 0°C and the mixture was stirred for 5h at room temperature. After this period the mixture was filtered over Celite® and evaporated under reduced pressure. The crude mixture was purified by silica gel gradient column chromatography (DCM/MeOH/NH_4OH 14:1:0.1) to afford **11c** as a white powder. ^{1}**H-NMR** (400 MHz, MeOD): 7.15 (d, 2H, Ar*H*, J= 8.4 Hz), 6.99 (d, 2H, Ar*H*, J= 8.4 Hz), 6.77-6.74 (m, 2H, Ar*H*), 6.71-6.67 (m, 2H, Ar*H*), 3.52 (s, 2H, ArCH_2N-), 2.75-2.71 (m, 2H, -NCH_2CH$_2$Ar), 2.61-2.57 (m, 2H, -NCH$_2$CH_2Ar), 2.28 (s, 3H, -NCH_3) ppm. ^{13}**C-NMR** (100 MHz, MeOD): 156.6, 155.3, 130.7, 130.4, 129.1, 127.5, 114.8, 114.6, 60.7, 58.7, 40.6, 31.7 ppm.

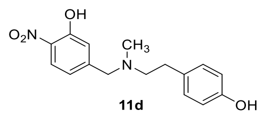

11d

5-(((4-hydroxyphenethyl)(methyl)amino)methyl)-2-nitrophenol ***11d:*** compound **iiie** (1.0 eq., 1.0 mmol) and formaldehyde (1.05 eq., 1.05 mmol) were dissolved in methanol (2.0 mL) and the solution was stirred for 4h at room temperature. Then $NaBH_4$ (1.1 eq., 1.1 mmol) was added at 0°C and the mixture was stirred for 5h at room temperature. After this period the mixture was filtered over Celite® and the solvent was evaporated under reduced pressure. The crude mixture was purified by silica gel gradient column chromatography (DCM/MeOH/NH_4OH 12:1:0.1) to afford **11d** as a yellow powder (93%). ^{1}**H-NMR** (400 MHz, MeOD): 8.02 (d, 1H, Ar*H*, J= 8.8 Hz), 7.10 (d, 1H, Ar*H*, J= 1.6 Hz), 7.01-6.98 (d, 2H, Ar*H*, J= 8.4 Hz), 6.96-6.93 (dd, 1H, Ar*H*, J= 8.4, 1.6 Hz), 6.71-6.76 (m, 2H, Ar*H*), 3.61 (s, 2H, ArCH_2N-), 2.76-2.72 (m, 2H, -NCH_2CH$_2$Ar), 2.64-2.60 (m, 2H, -NCH$_2$CH_2Ar), 2.31 (s, 3H, -NCH_3) ppm. ^{13}**C-NMR** (100 MHz, MeOD): 155.3, 154.5, 149.0, 133.3, 130.6, 129.2, 124.7, 120.0, 119.6, 114.7, 60.7, 59.2, 41.1, 32.2 ppm.

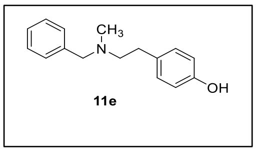

4-(2-(benzyl(methyl)amino)ethyl)phenol 11e: compound **iiif** (1.0 eq., 1 mmol) and formaldehyde (1.05 eq., 1.05 mmol) were dissolved in methanol (2.0 mL) and the solution was stirred for 4h at room temperature. Then $NaBH_4$ (1.1 eq., 1.1 mmol) was added at 0°C and the mixture was stirred for 5h at room temperature. After this period the mixture was filtered over Celite® and the solvent was evaporated under reduced pressure. The crude mixture was purified by silica gel gradient column chromatography (DCM/MeOH/NH_4OH 20:1:0.1) to afford **11e** as a white powder (98%). **^{1}H-NMR** (400 MHz, MeOD): 7.34-7.25 (m, 5H, Ar*H*), 6.99-6.96 (m, 2H, Ar*H*), 6.70-6.67 (m, 2H, Ar*H*), 3.58 (s, 2H, ArC*H*$_2$N-), 2.75-2.71 (m, 2H, -NC*H*$_2$CH$_2$Ar), 2.59-2.57 (m, 2H, -NCH$_2$C*H*$_2$Ar), 2.27 (s, 3H, -NC*H*$_3$) ppm. **^{13}C-NMR** (100 MHz, MeOD): 155.3, 137.5, 130.7, 129.2, 129.1, 127.9, 127.0, 114.8, 61.5, 59.0, 40.9, 31.9 ppm.

*SM #2.2. Synthesis and characterization of compound **11f***

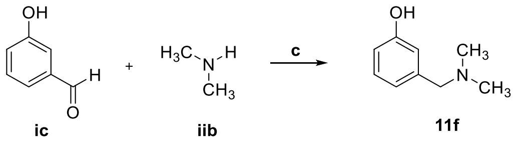

ic **iib** **11f**

c: Ti(O*i*-Pr)$_4$, $NaBH_4$, MeOH dry, r.t., argon (91%)

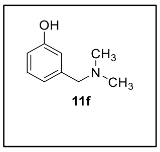

*3-((dimethylamino)methyl)phenol **11f***: 3-hydroxybenzaldehyde **ic** (1.0 eq., 1.0 mmol) was dissolved in dry methanol (2.0 mL). Ti(O*i*-Pr)$_4$ (1.3 eq., 1.3 mmol) and dimethylamine **iib** (1.1 eq., 1.1 mmol, 2M solution in methanol) was added and the mixture was stirred for 4h at room temperature under inert atmosphere. After this period, $NaBH_4$ (1.1 eq., 1.1 mmol) was added at 0°C and the mixture was stirred for 5h at room temperature, filtered over Celite® and evaporated under reduced pressure. The crude mixture was purified by silica gel gradient column chromatography (DCM/MeOH 10:1) to afford **11f** as a colorless oil (91%). **^{1}H-NMR** (400 MHz, MeOD): 7.14 (t, 1H, Ar*H*, J= 8.4, 8.0 Hz), 6.80-6.76 (m, 2H, Ar*H*), 6.71-6.68 (dd, 1H, Ar*H*, J= 8.0, 2.0 Hz), 3.63 (s, 2H, ArC*H*$_2$N-), 2.37 (s, 6H, -N(C*H*$_3$)$_2$) ppm. **^{13}C-NMR** (100 MHz, MeOD): 152.8, 135.6, 124.4, 114.5, 110.4, 109.2, 50.2, 29.3 ppm.

*SM #2.3. synthesis and characterization of compound **11g***

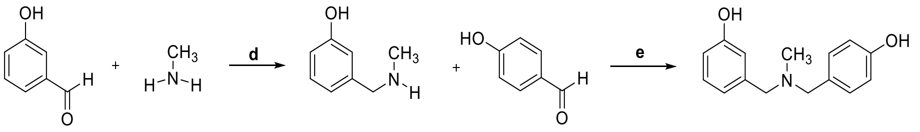

ic **iic** **iiig** **id** **11g**

d: $NaBH_4$, MeOH, r.t. (96%); **e**:Ti(Oi-Pr)$_4$, $NaBH_4$, MeOH dry, r.t., argon (82%)

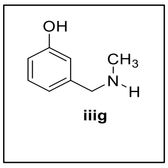

*3-((methylamino)methyl)phenol **iiig***:3-hydroxybenzaldehyde **ic** (1.0 eq., 1 mmol) and methylamine (1.1 eq., 1.1 mmol, 2M solution in methanol) were dissolved in methanol and the solution was stirred over night at room temperature. Then $NaBH_4$ (1.1 eq., 1.1 mmol) was added at 0°C and the mixture was stirred for 5h at room temperature. After this period the mixture was filtered over Celite® and the solvent was evaporated under reduced pressure. The crude mixture was purified by silica gel gradient column chromatography (DCM/MeOH/NH_4OH, 7:1:0.1) to afford **iiig** as colorless oil (96%). **^{1}H-NMR** (400 MHz, DMSO-*d*$_6$): 7.06 (t, 1H, Ar*H*, J= 7.6 Hz), 6.71-6.69 (m, 2H, Ar*H*), 6.60-6.58 (dd, 1H, Ar*H*, J= 7.6, 1.2 Hz), 3.53 (s, 2H, ArC*H*$_2$N-), 2.23 (s, 3H, -NC*H*$_3$), ppm. **^{13}C-NMR** (100 MHz, DMSO-*d*$_6$): 157.1, 141.9, 128.8, 118.3, 114.6, 113.2, 54.8, 35.3 ppm

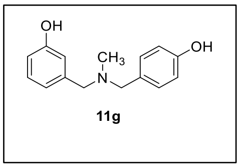

*3-(((4-hydroxybenzyl)(methyl)amino)methyl)phenol **11g**:* 4-hydroxybenzaldehyde **id** (1.0 eq., 1 mmol) was dissolvend in dry methanol (2 .0 mL). Then Ti(O*i*-Pr)$_4$ (1.3 eq., 1.3 mmol) and **iiig** (1.1 eq., 1.1 mmol) were added and the mixture was stirred for 4h at room temperature under inert atmosphere. After this period, $NaBH_4$ (1.1 eq., 1.1 mmol) was added at 0°C and the solution was stirred for 5h at room temperature. The solution was then filtered over Celite® and the solvent was evaporated under reduced pressure. The crude mixture was purified by silica gel gradient column chromatography (DCM/MeOH 10:1) to afford **11g** as a white powder (82%). **^{1}H-NMR** (400 MHz, MeOD): 7.17-7.12 (m, 3H, Ar*H*), 6.81-6.75 (m, 4H, Ar*H*), 6.72-6.70 (m, 1H, Ar*H*), 3.45 (s, 4H, ArC*H*$_2$NC*H*$_2$Ar), 2.17 (s, 3H, -NC*H*$_3$), ppm. **^{13}C-NMR** (100 MHz, MeOD): 157.1, 156.5, 139.2, 130.4, 128.9, 128.2, 120.3, 115.9, 114.6, 113.9, 60.9, 60.6, 40.7 ppm

SM #2.4. synthesis and characterization of compound ***11h***

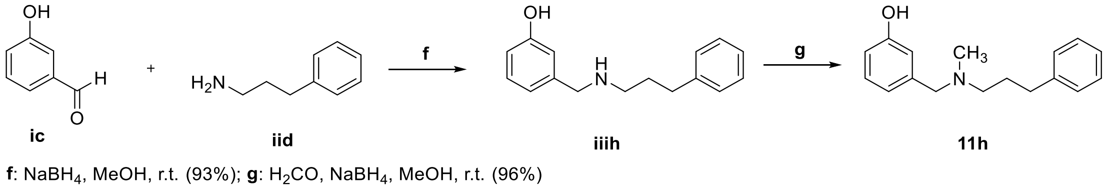

ic **iid** **iiih** **11h**

f: $NaBH_4$, MeOH, r.t. (93%); **g**: H_2CO, $NaBH_4$, MeOH, r.t. (96%)

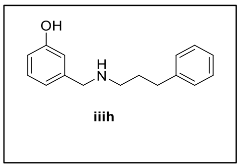

3-(((3-phenylpropyl)amino)methyl)phenol ***iiih:*** 3-hydroxybenzaldehyde **ic** (1.0 eq., 1 mmol) and 3-phenyl-1-propylamine **iid** were dissolved in methanol (2.0 mL) and the solution was stirred for 4h at room temperature. Then $NaBH_4$ (1.1 eq., 1.1 mmol) was added at 0°C and the mixture was stirred for 5h at room temperature. After this period the solution was filtered over Celite® and the solvent evaporated under reduced pressure. The crude mixture was purified by silica gel gradient column chromatography (DCM/MeOH/NH_4OH 20:1:0.1) to afford **iiih** as a white powder (93%).**^{1}H-NMR** (400 MHz, MeOD): 7.27-7.11 (m, 6H,), 6.78-6.76 (m, 2H, Ar*H*), 6.71-6.68 (m, 1H, Ar*H*), 3.65 (s, 2H, ArC*H*$_2$N-), 2.65-2.58 (m, 4H, -NC*H*$_2$CH$_2$C*H*$_2$Ar), 1.87-1.80 (m, 2H, -NCH$_2$C*H*$_2$CH$_2$Ar) ppm. **^{13}C-NMR** (100 MHz, MeOD): 157.3, 141.8, 140.5, 129.0, 128.0, 127.9, 125.4, 119.1, 115.0, 113.7, 52.9, 33.2, 30.7 ppm.

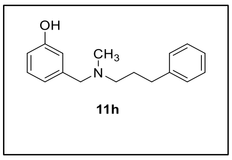

3-((methyl(3-phenylpropyl)amino)methyl)phenol ***11h:*** compound **iiih** (1.0 eq., 1 mmol) and formaldehyde (1.05 eq., 1.05 mmol) were dissolved in methanol (2.0 mL) and the solution was stirred for 5h at room temperature. Then $NaBH_4$ (1.1 eq., 1.1 mmol) was added at 0°C and the mixture was stirred for 5h at room temperature. After this period the mixture was filtered over Celite® and the solvent evaporated under reduced pressure. The crude mixture was purified by silica gel gradient column chromatography (DCM/MeOH/NH_4OH 9:1:0.1) to afford **11h** as a white powder (96%). **^{1}H-NMR** (400 MHz, MeOD): 7.27-7.23 (m, 2H, Ar*H*), 7.19-7.11 (m, 4H, Ar*H*), 6.78-6.76 (m, 2H, Ar*H*), 6.72-6.69 (m, 1H, Ar*H*), 3.47 (s, 2H, ArC*H*$_2$N-), 2.65-2.61 (t, 2H, -NC*H*$_2$CH$_2$CH$_2$Ar, J= 8.0 Hz), 2.46-2.42 (m, 2H, -NCH$_2$CH$_2$C*H*$_2$Ar), 2.22 (s, 3H, -NC*H*$_3$), 1.91-1.82 (m, 2H, -NCH$_2$C*H*$_2$CH$_2$Ar) ppm. **^{13}C-NMR** (100 MHz, MeOD): 157.1, 141.8, 138.9, 128.8, 128.0, 127.9, 125.4, 120.4, 116.0, 113.9, 61.6, 56.3, 41.0, 33.1, 28.3 ppm.

SM #3. NMR spectra.

SM #3.1. NMR specra of compounds ***iiia-h***

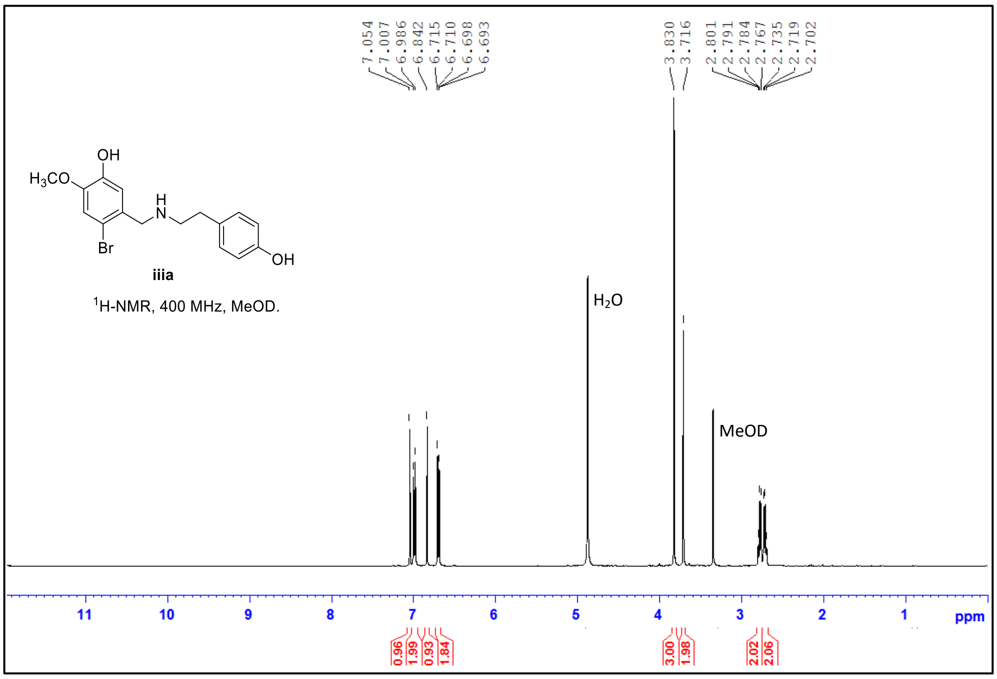

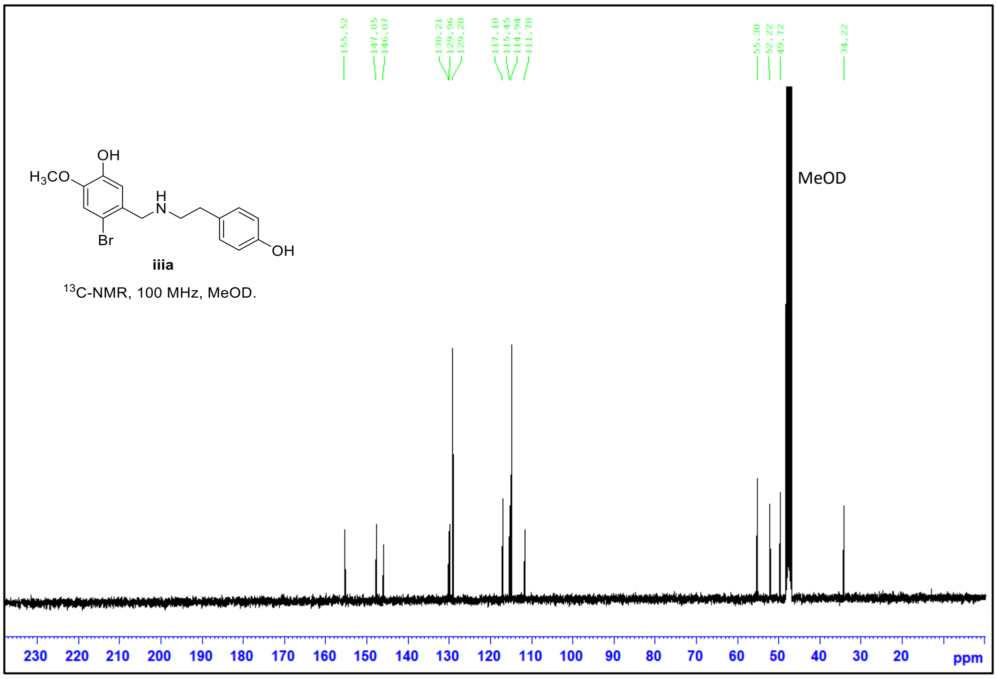

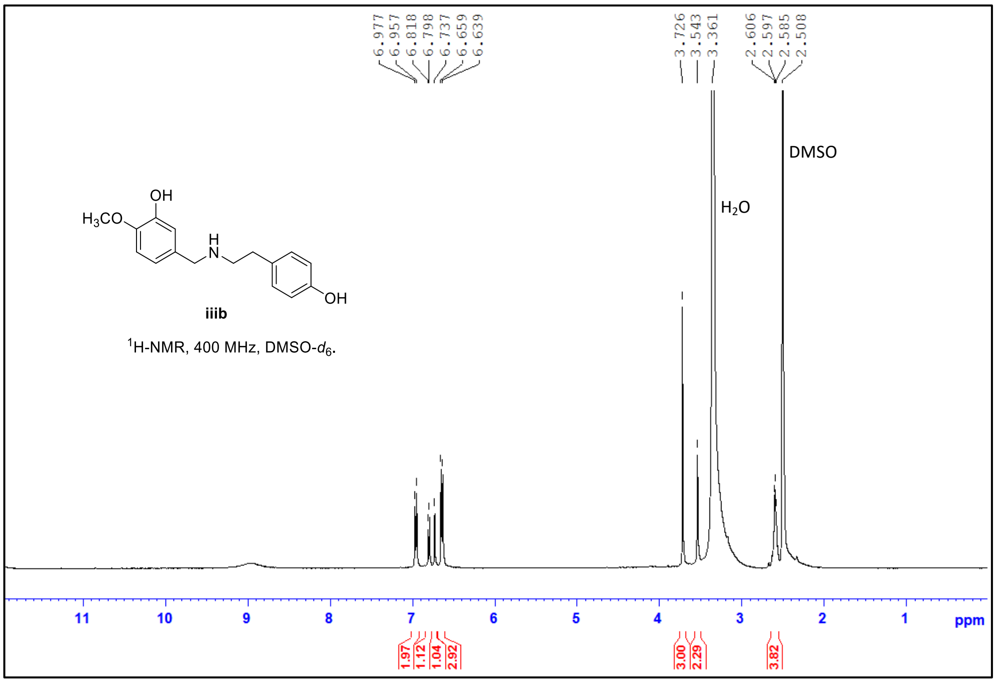
6.977
6.957
6.818
6.798
6.737
6.659
6.639
3.726
3.543
3.361
2.606
2.597
2.585
2.508
DMSO
H2O
OH
H3CO
H
N
OH
iiib
1H-NMR, 400 MHz, DMSO-d6.
11
10
9
8
7
6
5
4
3
2
1
ppm
1.97
1.12
1.04
2.92
3.00
2.29
3.82

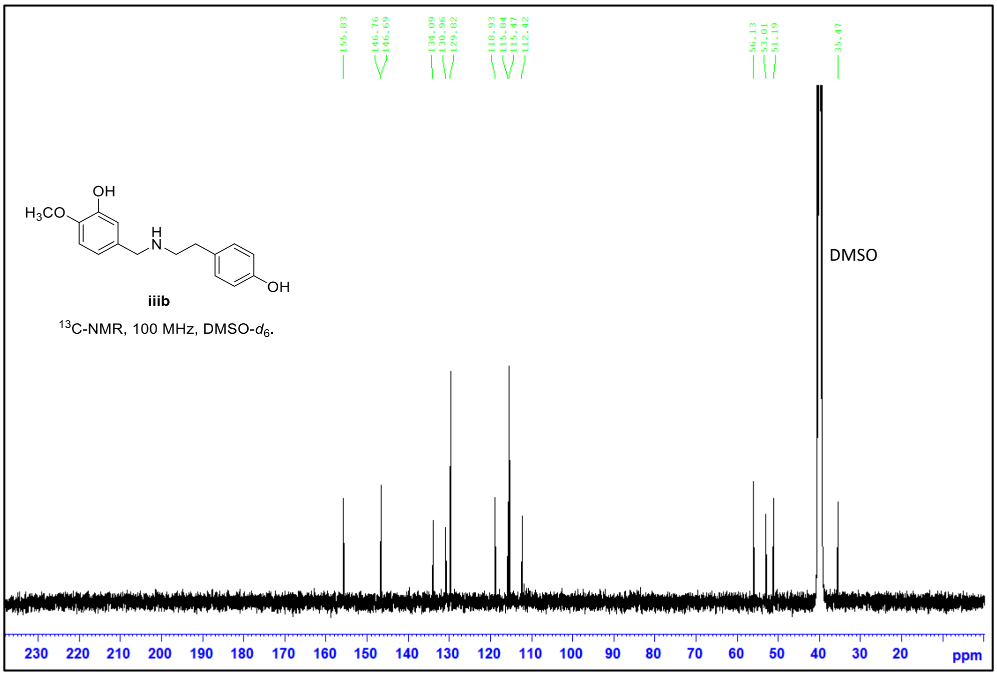
155.03
146.76
146.69
134.09
130.06
129.82
118.93
116.04
115.47
112.42
56.13
53.01
51.19
35.47
OH
H3CO
H
N
OH
iiib
13C-NMR, 100 MHz, DMSO-d6.
DMSO
230
220
210
200
190
180
170
160
150
140
130
120
110
100
90
80
70
60
50
40
30
20
ppm

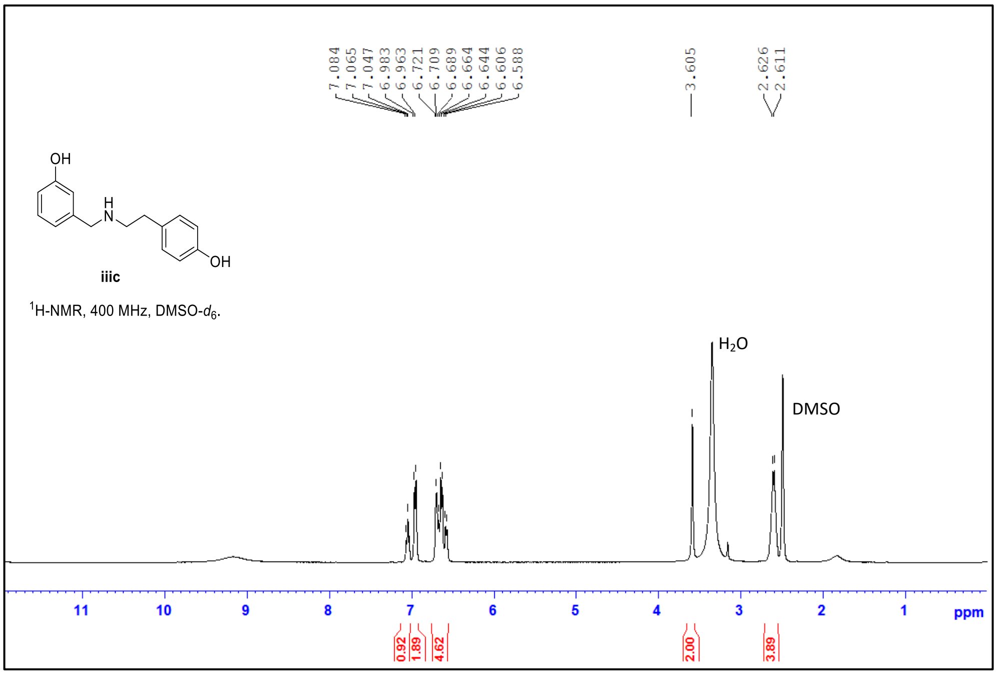
7.084
7.065
7.047
6.983
6.963
6.721
6.709
6.689
6.664
6.644
6.606
6.588
3.605
2.626
2.611
OH
H
N
OH
iiic
1H-NMR, 400 MHz, DMSO-d6.
H2O
DMSO
11
10
9
8
7
6
5
4
3
2
1
ppm
0.92
1.89
4.62
2.00
3.89

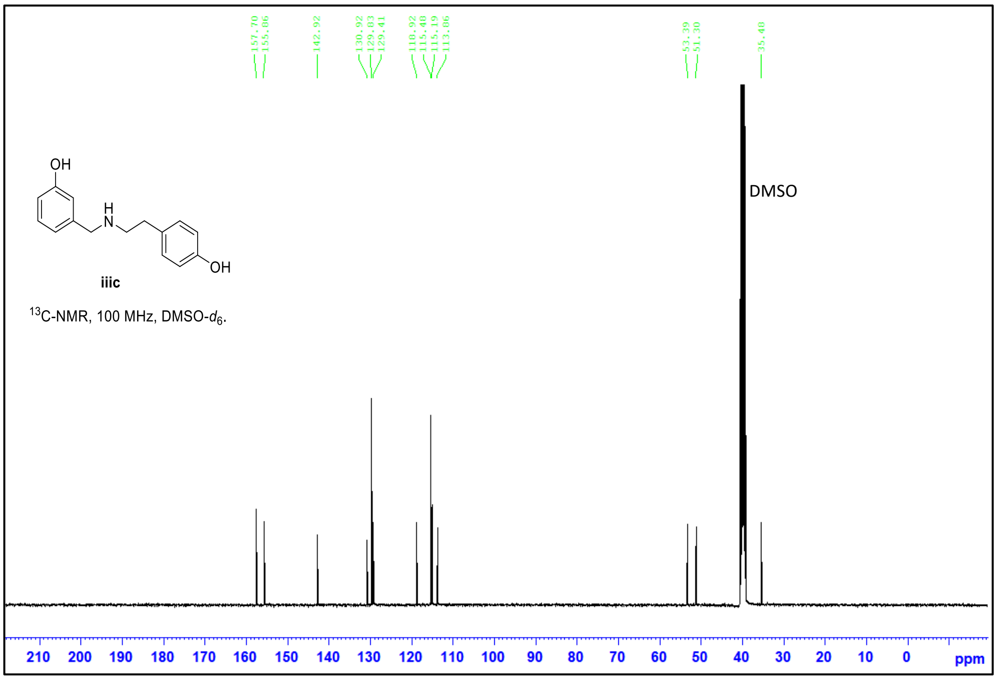
157.70
155.86
142.92
130.92
129.83
129.41
118.92
115.48
115.19
113.86
53.39
51.30
35.48
OH
H
N
OH
iiic
13C-NMR, 100 MHz, DMSO-d6.
DMSO
210
200
190
180
170
160
150
140
130
120
110
100
90
80
70
60
50
40
30
20
10
0
ppm

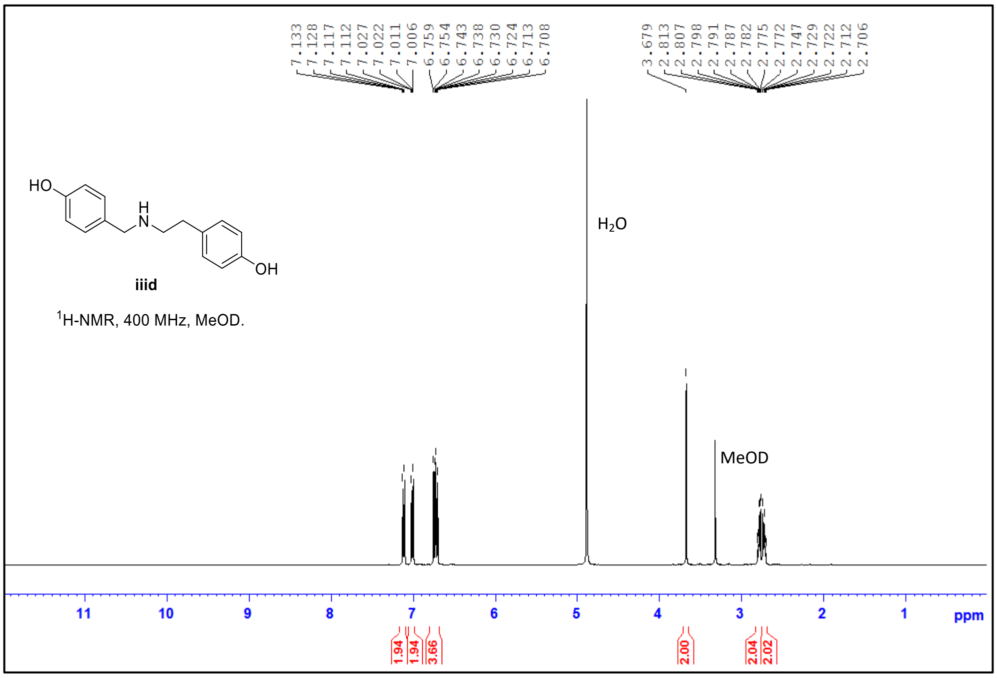
7.133
7.128
7.117
7.112
7.027
7.022
7.011
7.006
6.759
6.754
6.743
6.738
6.730
6.724
6.713
6.708
3.679
2.813
2.807
2.798
2.791
2.787
2.782
2.775
2.772
2.747
2.729
2.722
2.712
2.706
HO
H
N
OH
iiid
1H-NMR, 400 MHz, MeOD.
H2O
MeOD
11
10
9
8
7
6
5
4
3
2
1
ppm
1.94
1.94
3.66
2.00
2.04
2.02

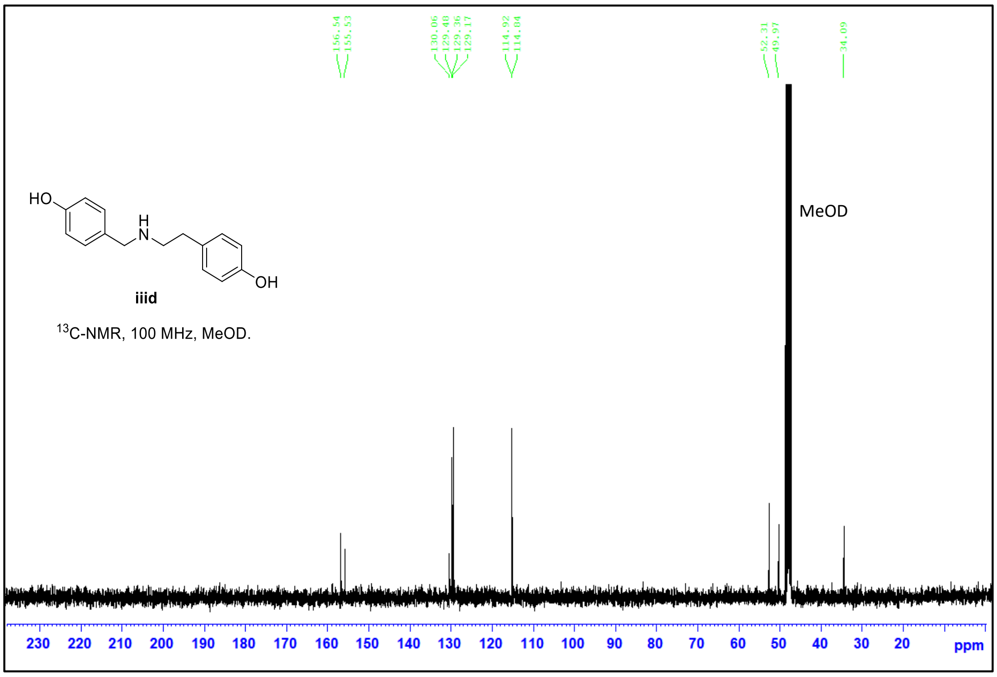
156.54
155.53
130.06
129.48
129.36
129.17
114.92
114.84
52.31
49.97
34.09
HO
H
N
OH
iiid
13C-NMR, 100 MHz, MeOD.
MeOD
230
220
210
200
190
180
170
160
150
140
130
120
110
100
90
80
70
60
50
40
30
20
ppm

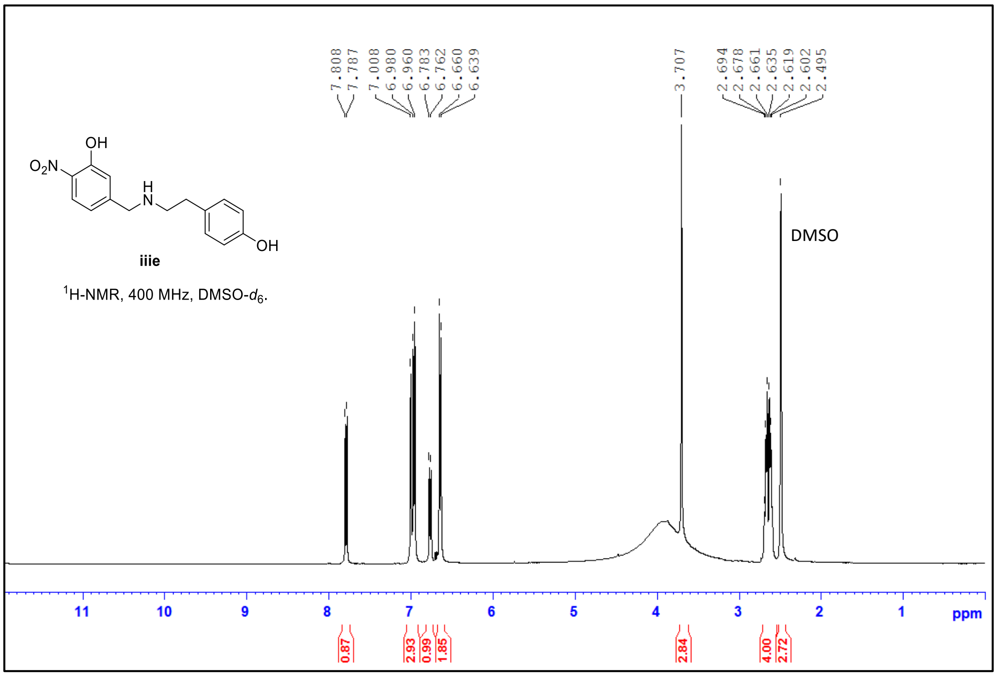
7.808
7.787
7.008
6.980
6.960
6.783
6.762
6.660
6.639
3.707
2.694
2.678
2.661
2.635
2.619
2.602
2.495
OH
O2N
H
N
OH
iiie
1H-NMR, 400 MHz, DMSO-d6.
DMSO
11 10 9 8 7 6 5 4 3 2 1 ppm
0.87
2.93
0.99
1.85
2.84
4.00
2.72

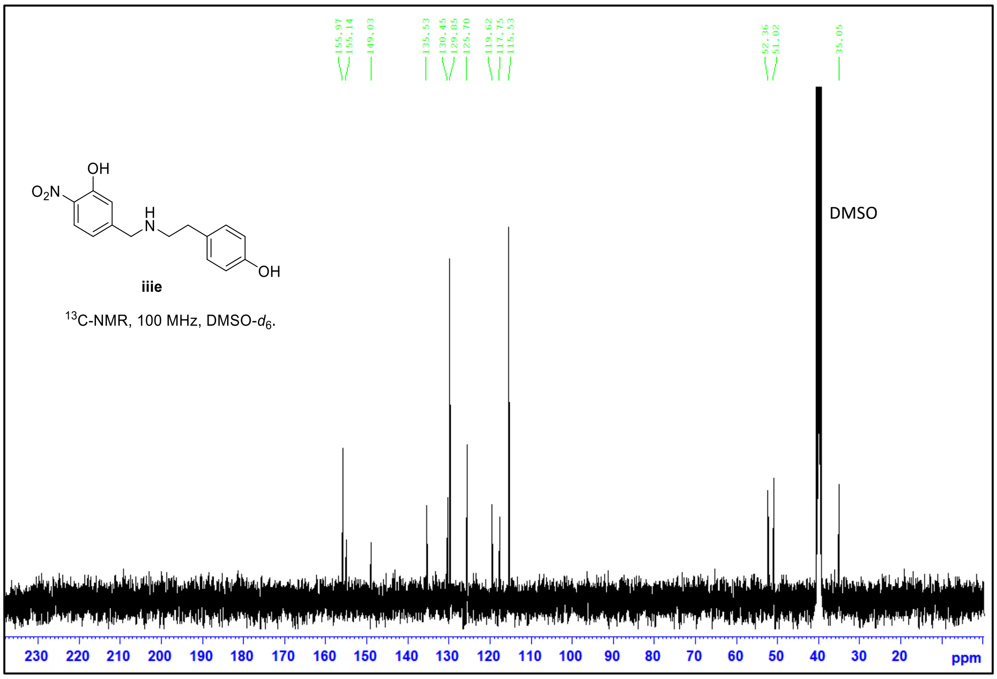
OH
O2N
H
N
OH
iiie
13C-NMR, 100 MHz, DMSO-d6.
DMSO
230 220 210 200 190 180 170 160 150 140 130 120 110 100 90 80 70 60 50 40 30 20 ppm

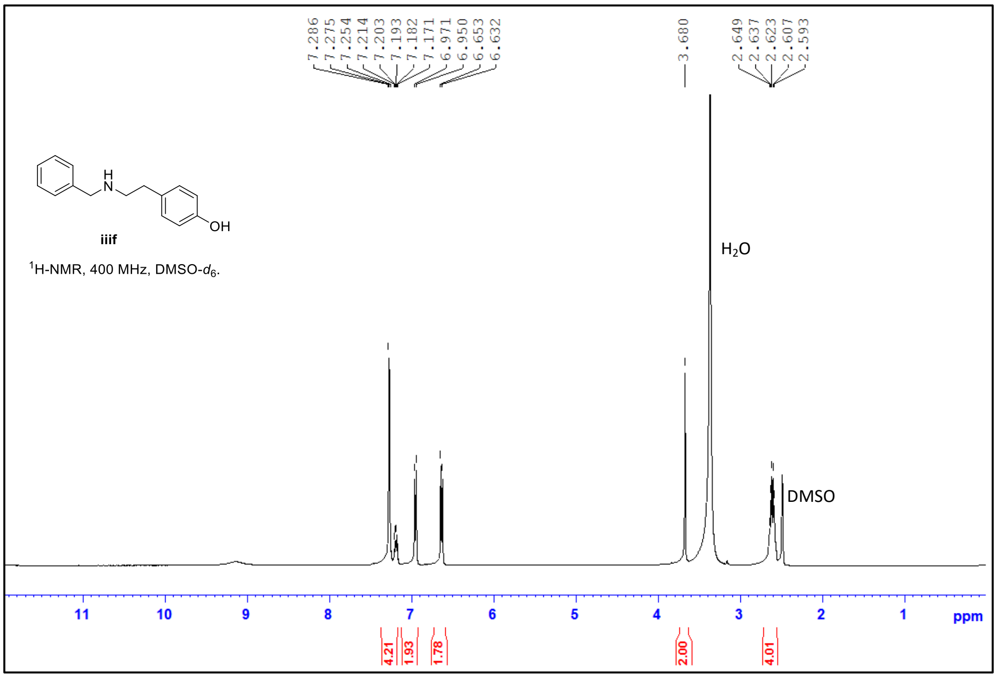

7.286
7.275
7.254
7.214
7.203
7.193
7.182
7.171
6.971
6.950
6.653
6.632
3.680
2.649
2.637
2.623
2.607
2.593
H
N
OH
iiif
^{1}H-NMR, 400 MHz, DMSO-d_6.
H_2O
DMSO
11 10 9 8 7 6 5 4 3 2 1 ppm
4.21
1.93
1.78
2.00
4.01

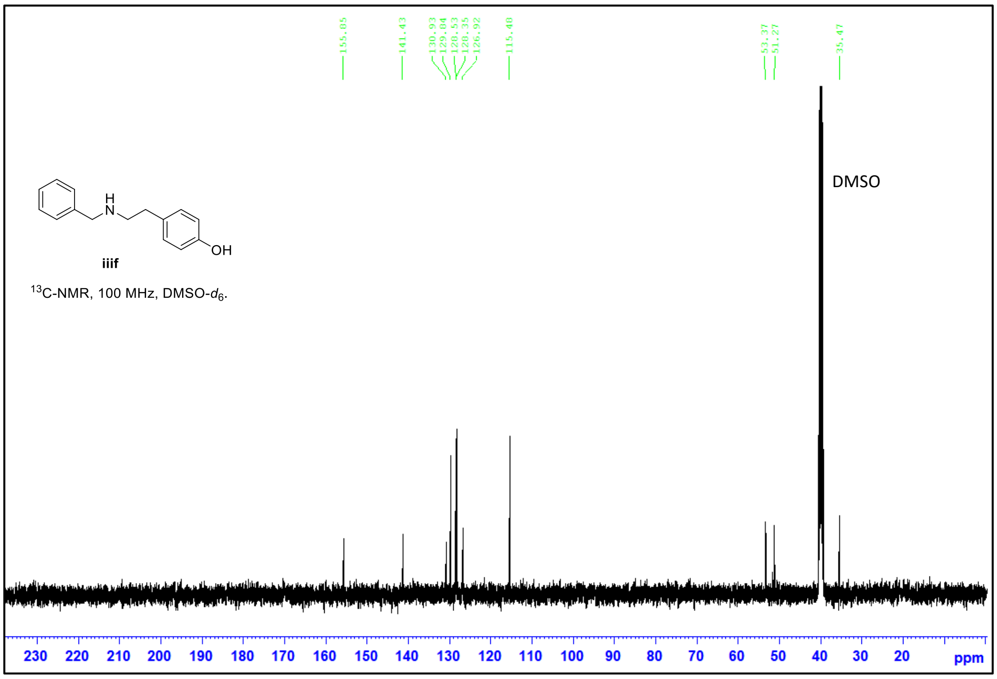

155.85
141.43
130.93
129.04
128.53
128.35
126.92
115.40
53.37
51.27
35.47
H
N
OH
iiif
^{13}C-NMR, 100 MHz, DMSO-d_6.
DMSO
230 220 210 200 190 180 170 160 150 140 130 120 110 100 90 80 70 60 50 40 30 20 ppm

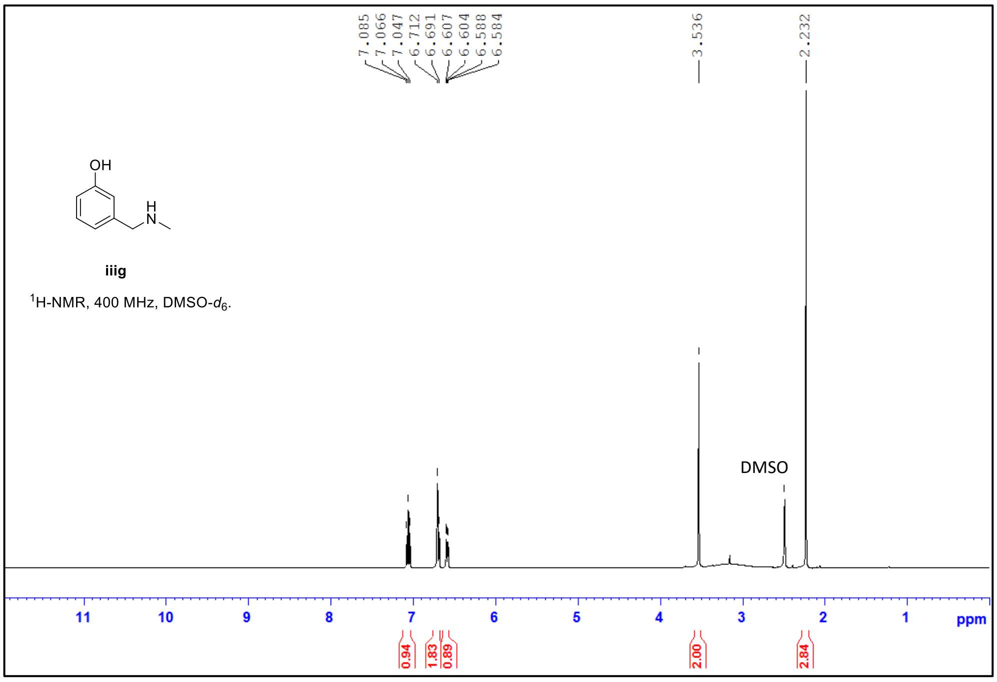

7.085
7.066
7.047
6.712
6.691
6.607
6.604
6.588
6.584
3.536
2.232
OH
H
N
iiig
1H-NMR, 400 MHz, DMSO-d6.
DMSO
11
10
9
8
7
6
5
4
3
2
1
ppm
0.94
1.83
0.89
2.00
2.84

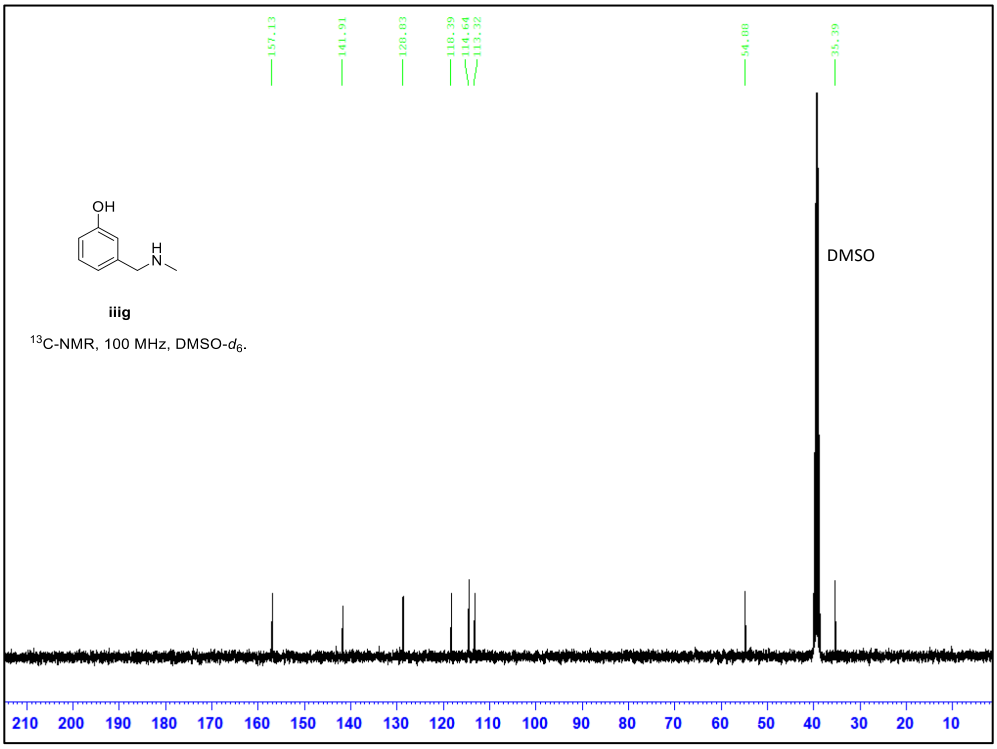

157.13
141.91
129.03
118.39
114.64
113.32
54.00
35.39
OH
H
N
iiig
13C-NMR, 100 MHz, DMSO-d6.
DMSO
210
200
190
180
170
160
150
140
130
120
110
100
90
80
70
60
50
40
30
20
10

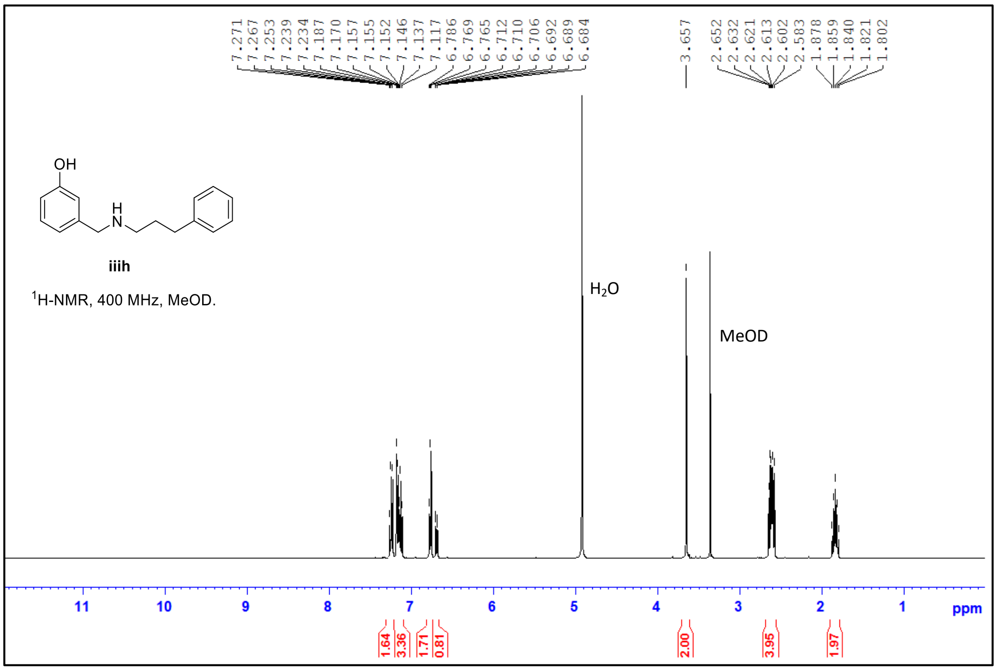
OH
H
N
iiih
^{1}H-NMR, 400 MHz, MeOD.
H_2O
MeOD
ppm

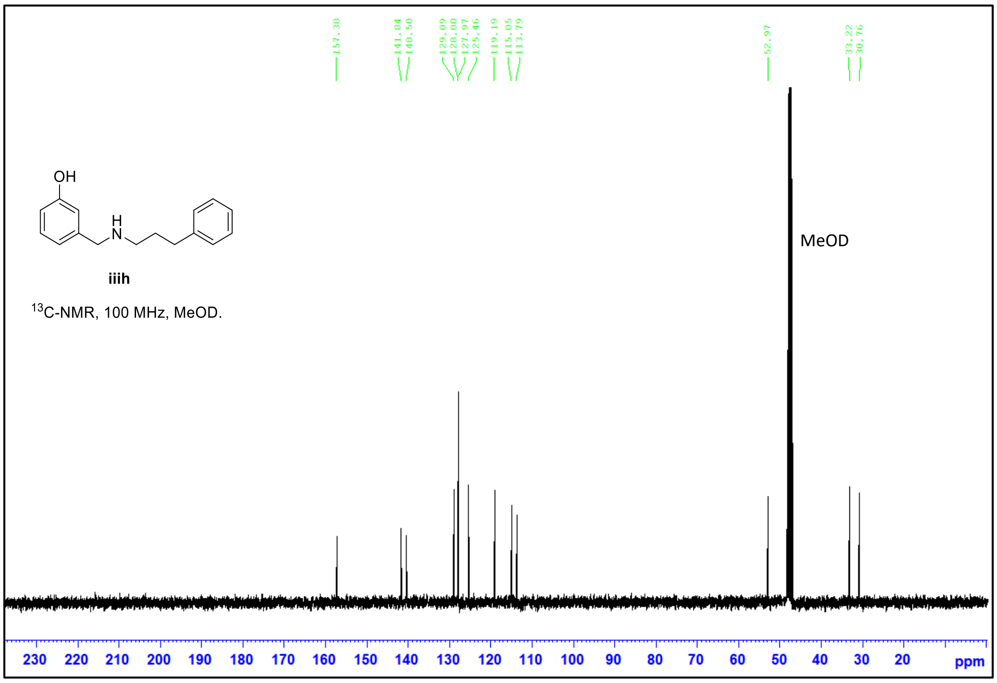
OH
H
N
iiih
^{13}C-NMR, 100 MHz, MeOD.
MeOD
ppm

*SM #3.2. NMR specra of compounds **7b** and **11a-h***

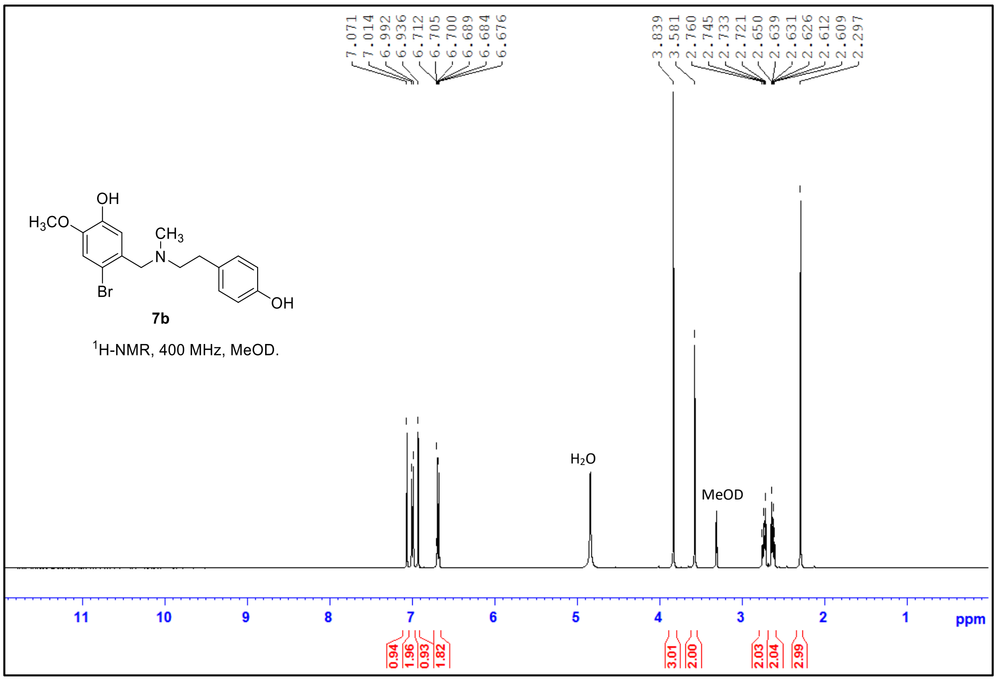

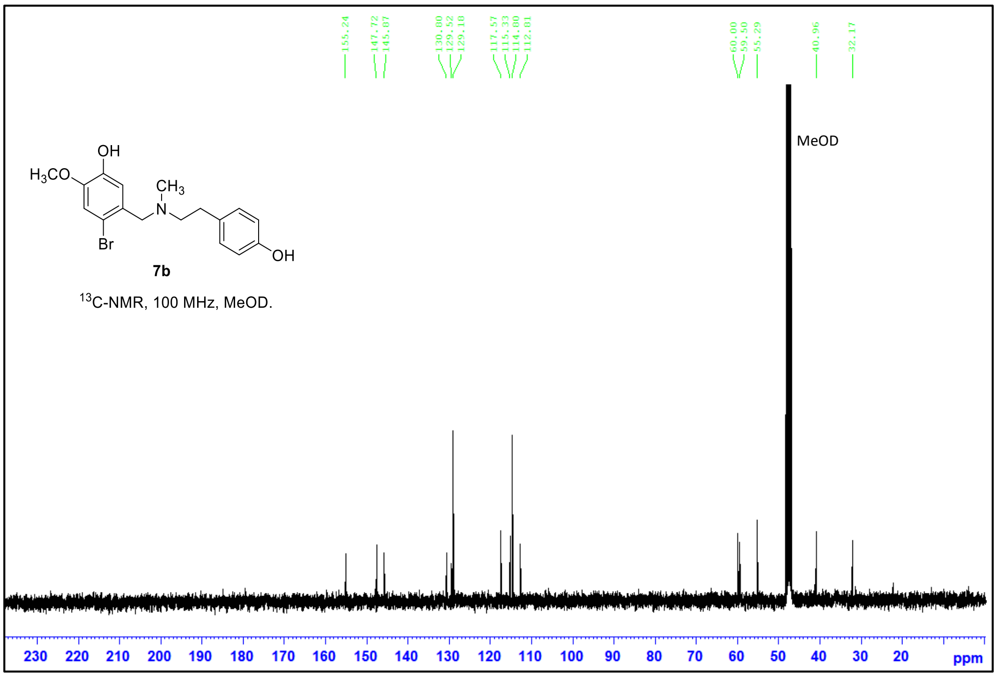

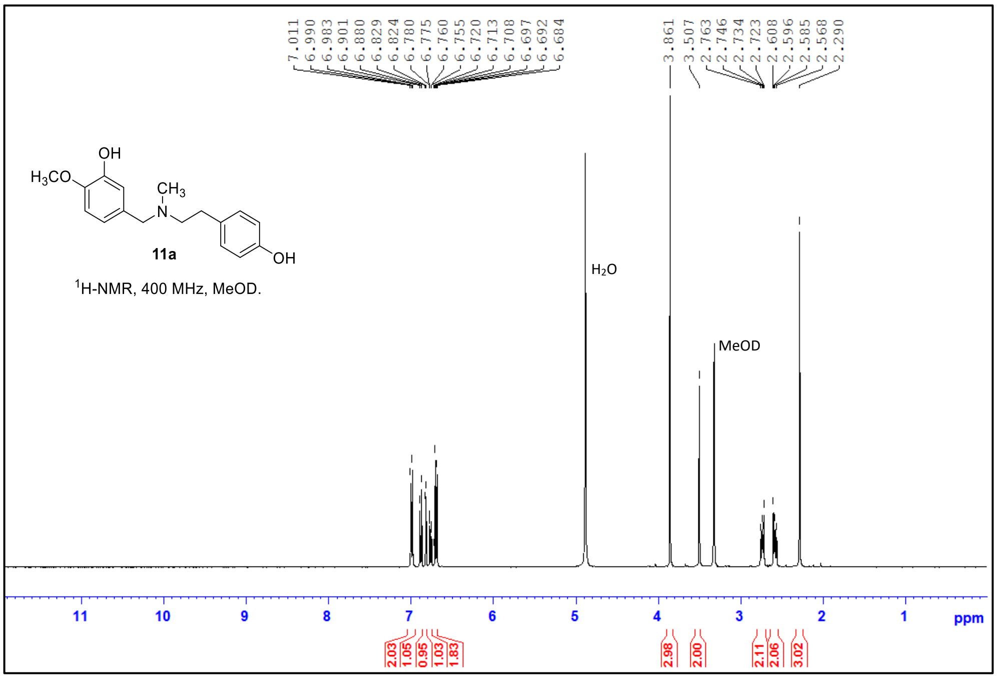
7.011 6.990 6.983 6.901 6.880 6.829 6.824 6.780 6.775 6.760 6.755 6.720 6.713 6.708 6.697 6.692 6.684
3.861 3.507 2.763 2.746 2.734 2.723 2.608 2.596 2.585 2.568 2.290
OH
H3CO
CH3
N
OH
11a
1H-NMR, 400 MHz, MeOD.
H2O
MeOD
11 10 9 8 7 6 5 4 3 2 1 ppm
2.03 1.05 0.95 1.03 1.83
2.98 2.00
2.11 2.06 3.02

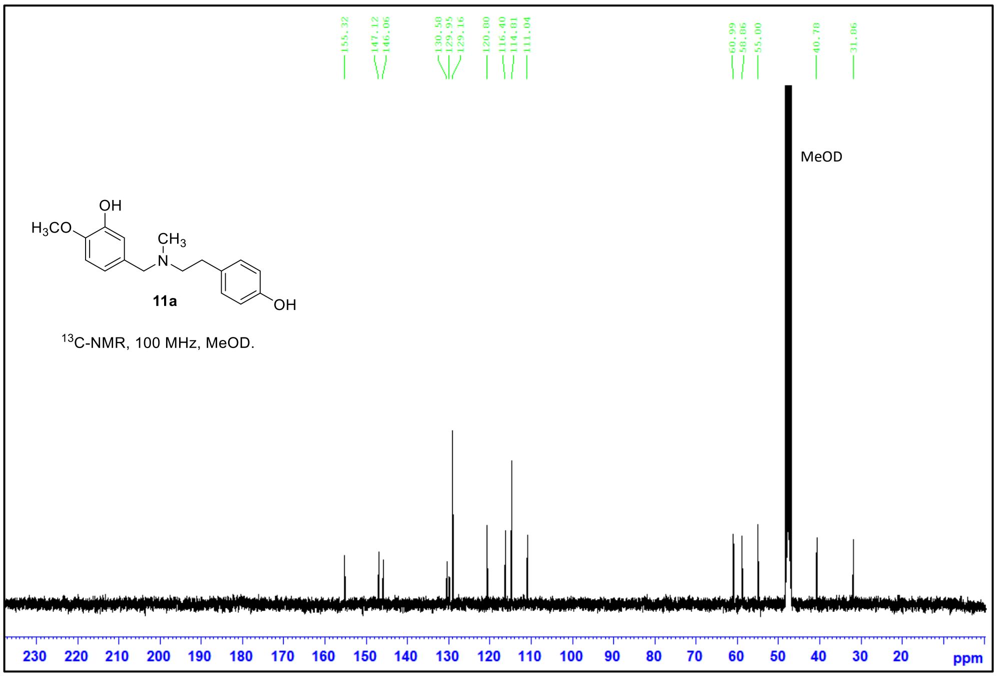
155.32 147.12 146.06 130.58 129.95 129.16 120.80 116.40 114.81 111.04
60.99 58.86 55.00 40.78 31.86
MeOD
OH
H3CO
CH3
N
OH
11a
13C-NMR, 100 MHz, MeOD.
230 220 210 200 190 180 170 160 150 140 130 120 110 100 90 80 70 60 50 40 30 20 ppm

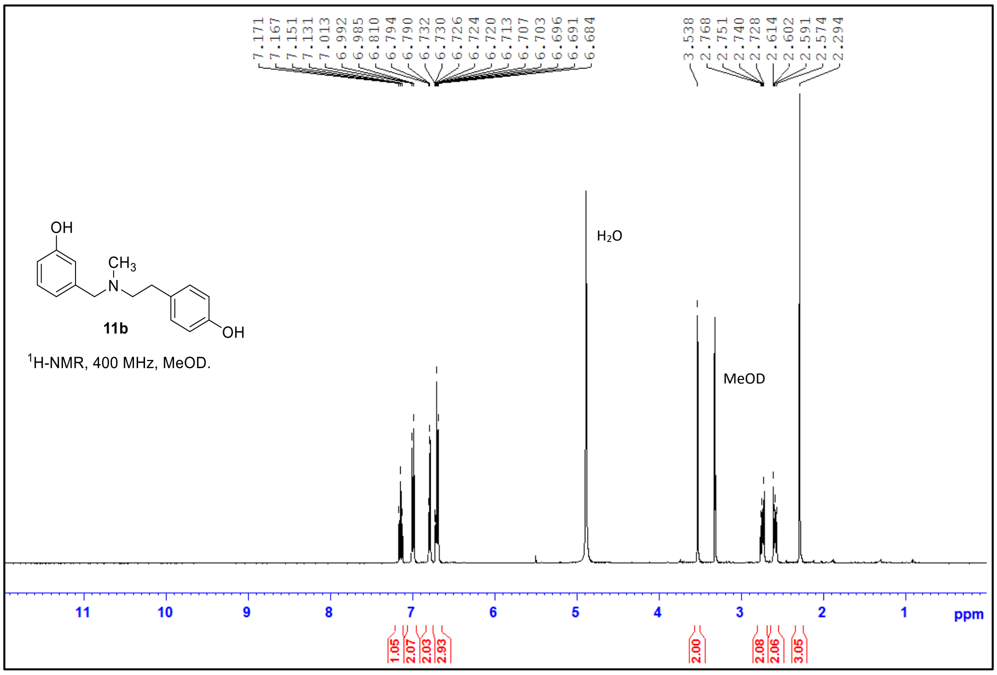
7.171
7.167
7.151
7.131
7.013
6.992
6.985
6.810
6.794
6.790
6.732
6.730
6.726
6.724
6.720
6.713
6.707
6.703
6.696
6.691
6.684
3.538
2.768
2.751
2.740
2.728
2.614
2.602
2.591
2.574
2.294
OH
CH3
N
OH
11b
1H-NMR, 400 MHz, MeOD.
H2O
MeOD
11
10
9
8
7
6
5
4
3
2
1
ppm
1.05
2.07
2.03
2.93
2.00
2.08
2.06
3.05

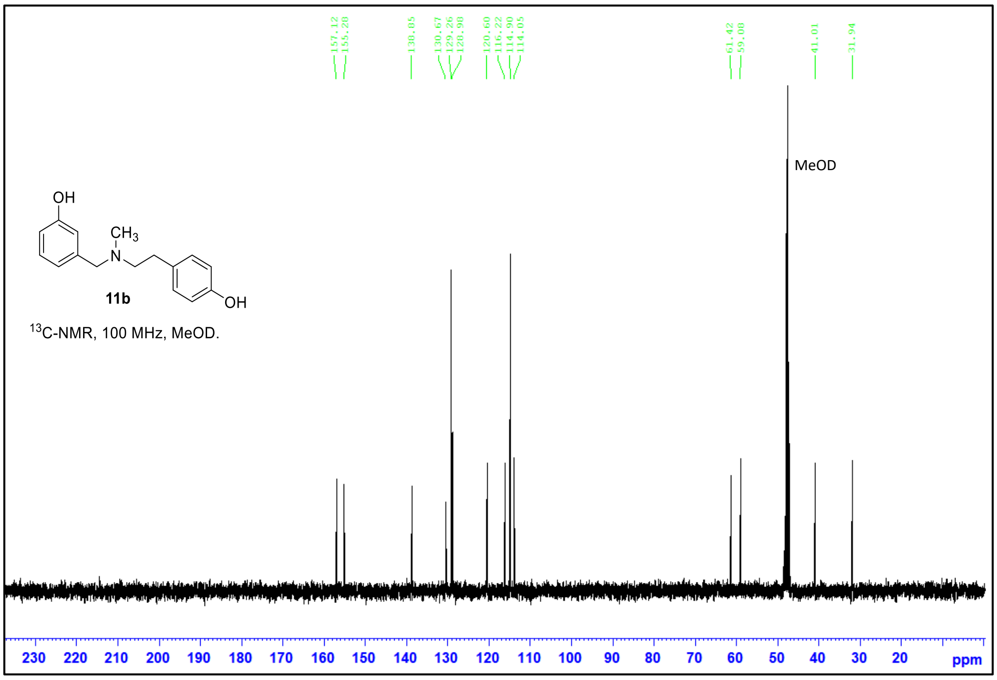
157.12
155.20
139.05
130.67
129.26
120.90
120.60
116.22
114.90
114.05
61.42
59.00
41.01
31.94
MeOD
OH
CH3
N
OH
11b
13C-NMR, 100 MHz, MeOD.
230
220
210
200
190
180
170
160
150
140
130
120
110
100
90
80
70
60
50
40
30
20
ppm

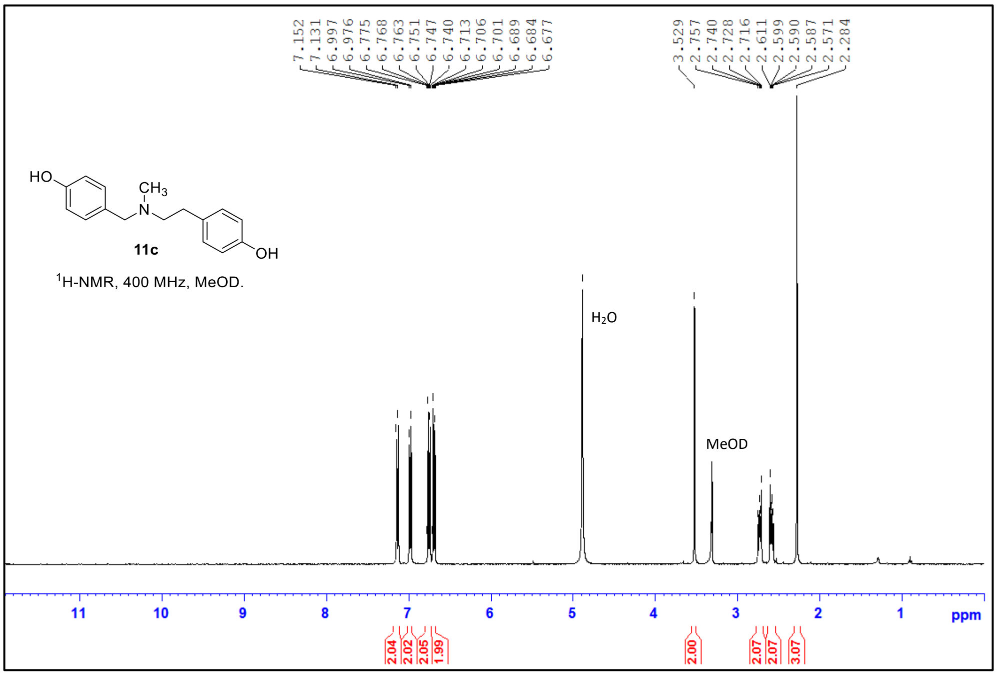
7.152 7.131 6.997 6.976 6.775 6.768 6.763 6.751 6.747 6.740 6.713 6.706 6.701 6.689 6.684 6.677
3.529 2.757 2.740 2.728 2.716 2.611 2.599 2.590 2.587 2.571 2.284
HO
CH3
N
OH
11c
1H-NMR, 400 MHz, MeOD.
H2O
MeOD
11 10 9 8 7 6 5 4 3 2 1 ppm
2.04 2.02 2.05 1.99 2.00 2.07 2.07 3.07

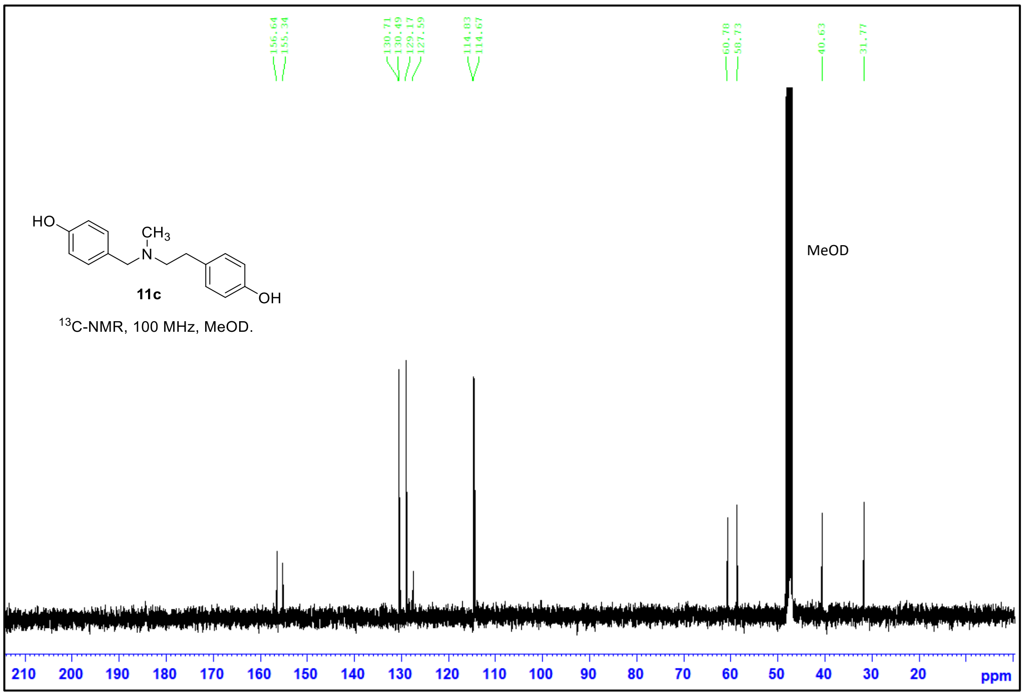
156.64 155.34 130.71 130.49 129.17 127.59 114.83 114.67 60.78 58.73 40.63 31.77
HO
CH3
N
OH
11c
13C-NMR, 100 MHz, MeOD.
MeOD
210 200 190 180 170 160 150 140 130 120 110 100 90 80 70 60 50 40 30 20 ppm

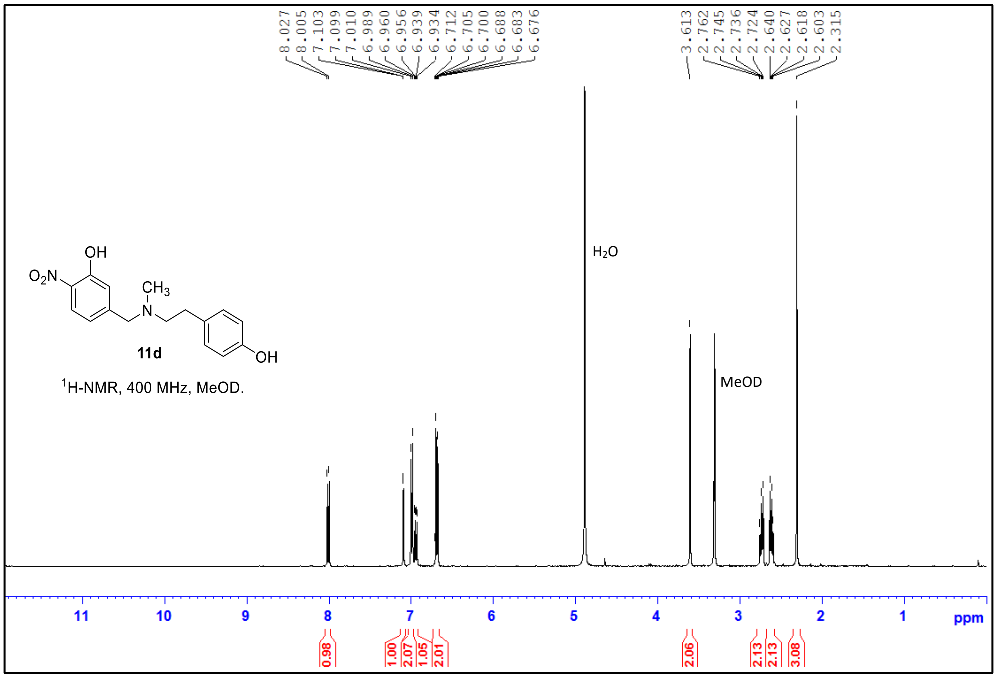
8.027
8.005
7.103
7.099
7.010
6.989
6.960
6.956
6.939
6.934
6.712
6.705
6.700
6.688
6.683
6.676
3.613
2.762
2.745
2.736
2.724
2.640
2.627
2.618
2.603
2.315
H2O
MeOD
11d
1H-NMR, 400 MHz, MeOD.
ppm
0.98
1.00
2.07
1.05
2.01
2.06
2.13
2.13
3.08

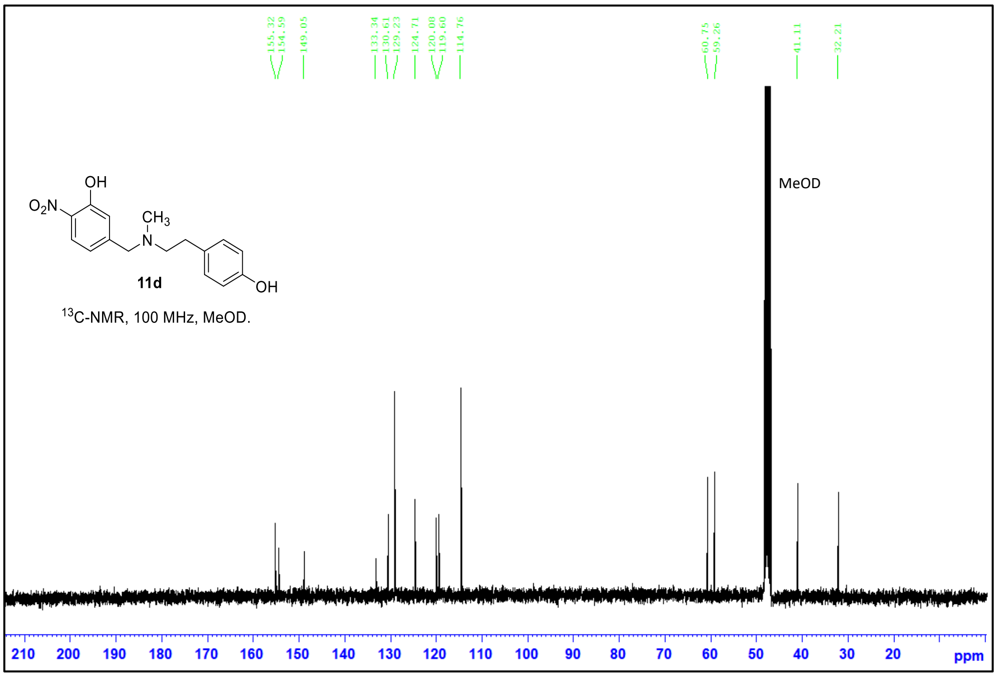
155.32
154.59
149.05
132.34
130.61
129.23
124.71
120.08
119.60
114.76
60.75
59.26
41.11
32.21
MeOD
11d
13C-NMR, 100 MHz, MeOD.
ppm

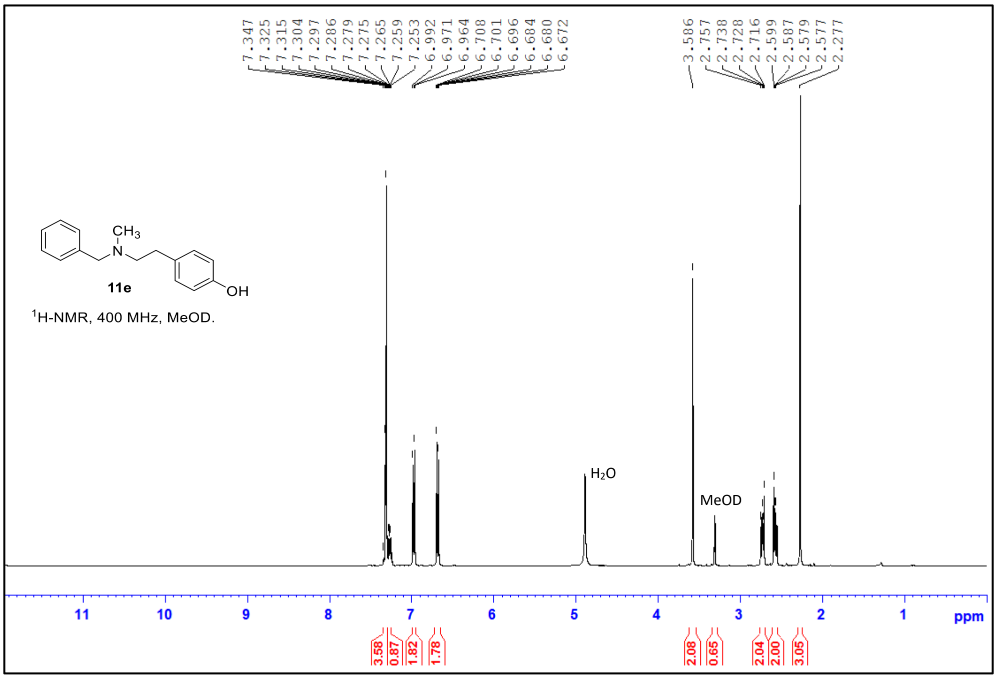
7.347
7.325
7.315
7.304
7.297
7.286
7.279
7.275
7.265
7.259
7.253
6.992
6.971
6.964
6.708
6.701
6.696
6.684
6.680
6.672
3.586
2.757
2.738
2.728
2.716
2.599
2.587
2.579
2.577
2.277
CH3
N
OH
11e
1H-NMR, 400 MHz, MeOD.
H2O
MeOD
11
10
9
8
7
6
5
4
3
2
1
ppm
3.58
0.87
1.82
1.78
2.08
0.65
2.04
2.00
3.05

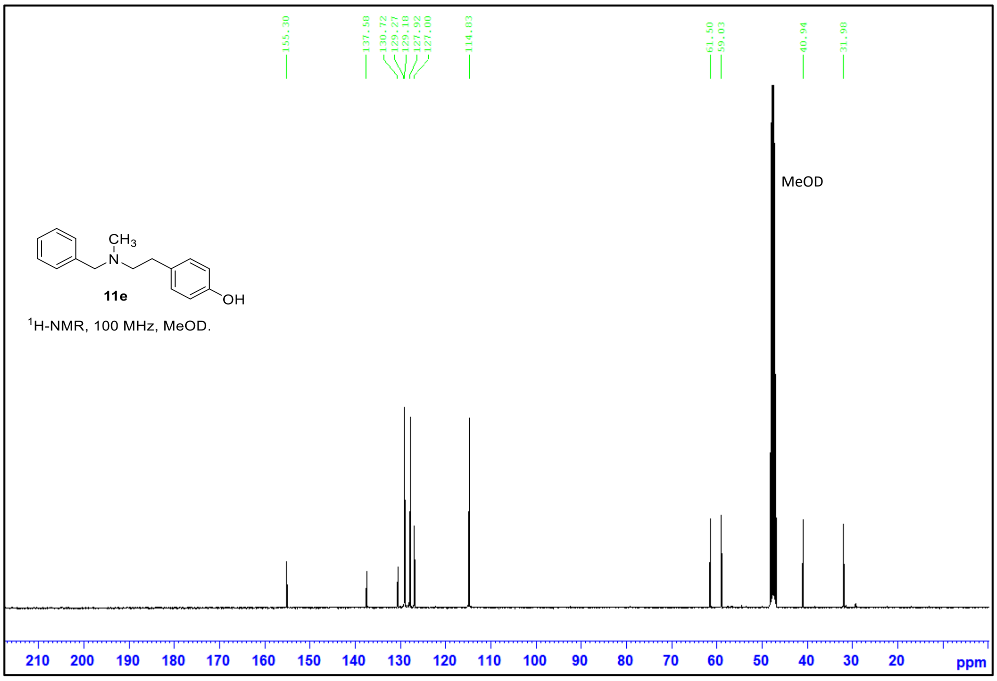
155.30
137.58
130.72
129.27
129.18
127.92
127.00
114.03
61.50
59.03
40.94
31.90
MeOD
CH3
N
OH
11e
1H-NMR, 100 MHz, MeOD.
210
200
190
180
170
160
150
140
130
120
110
100
90
80
70
60
50
40
30
20
ppm

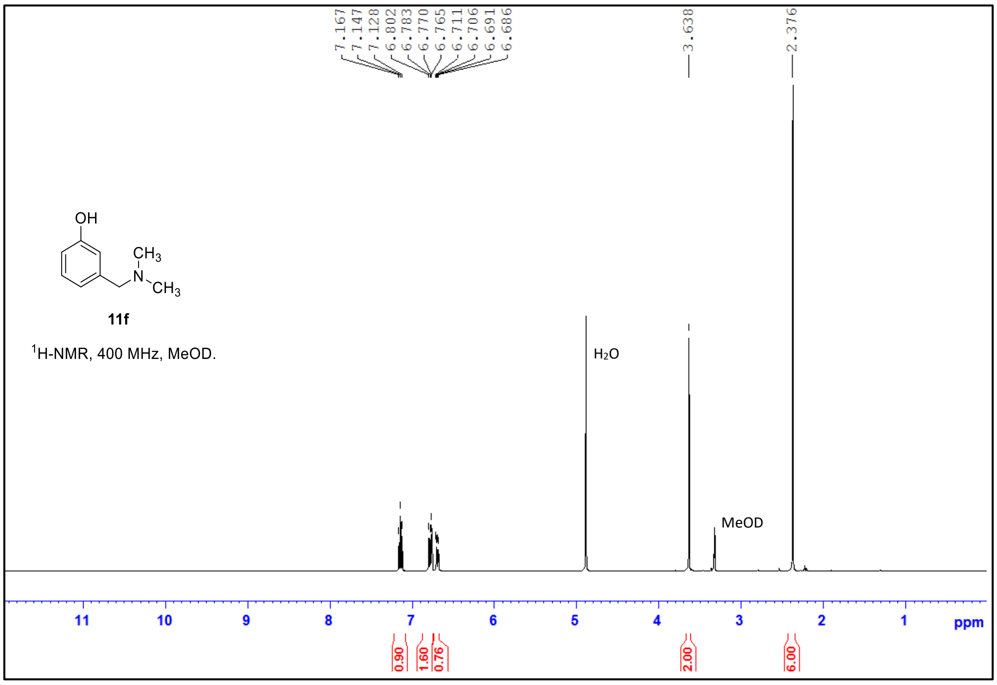
7.167
7.147
7.128
6.802
6.783
6.770
6.765
6.711
6.706
6.691
6.686
3.638
2.376
OH
CH3
N
CH3
11f
1H-NMR, 400 MHz, MeOD.
H2O
MeOD
11
10
9
8
7
6
5
4
3
2
1
ppm
0.90
1.60
0.76
2.00
6.00

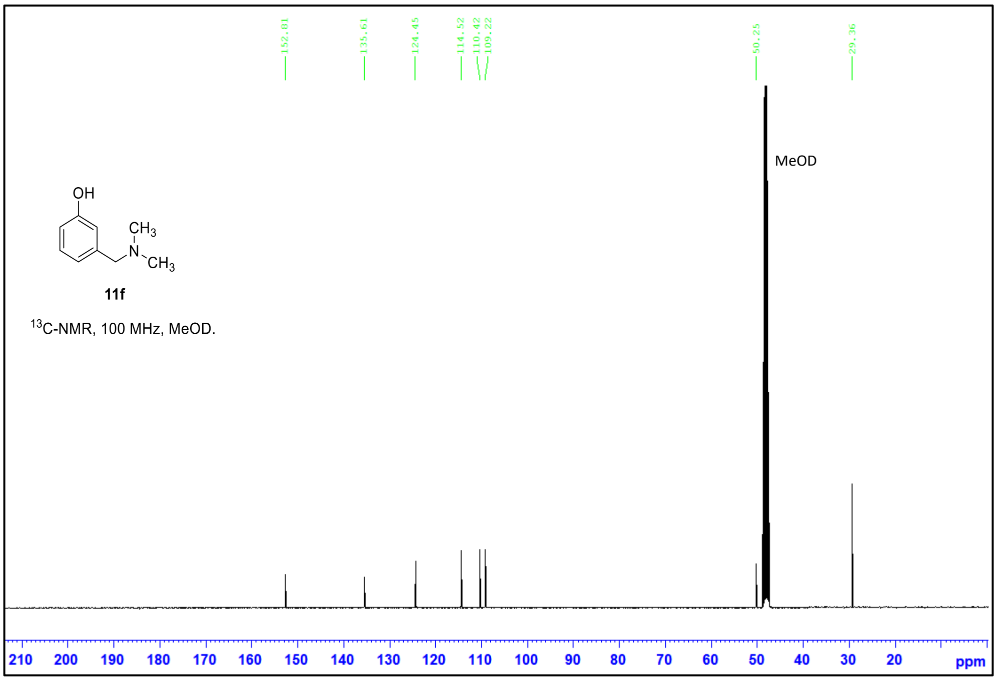
152.01
135.61
124.45
114.52
110.42
109.22
50.25
29.36
MeOD
OH
CH3
N
CH3
11f
13C-NMR, 100 MHz, MeOD.
210
200
190
180
170
160
150
140
130
120
110
100
90
80
70
60
50
40
30
20
ppm

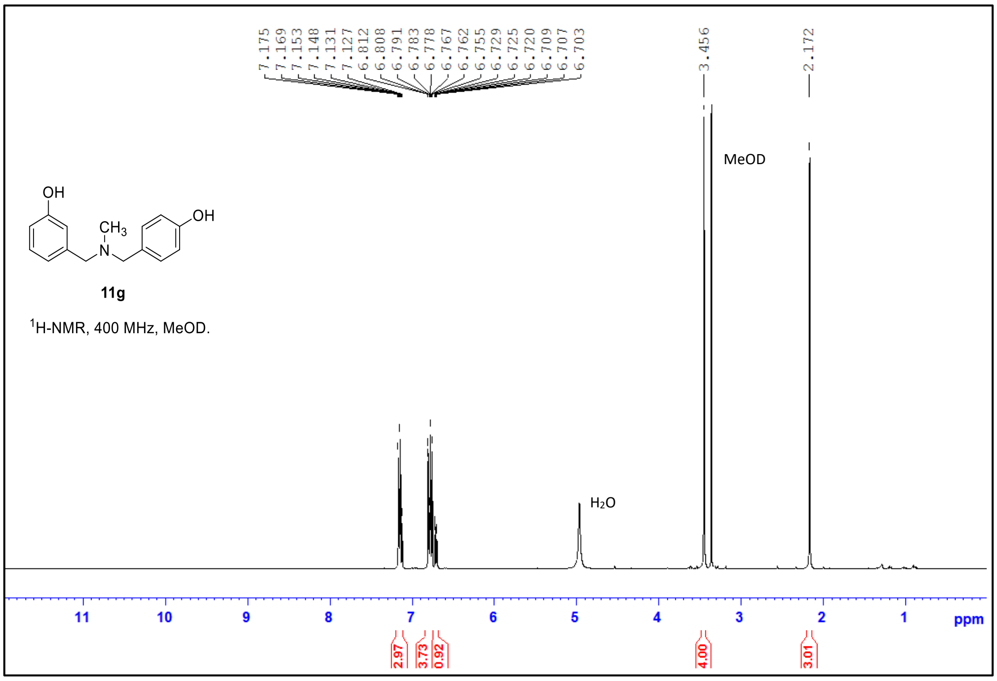
7.175
7.169
7.153
7.148
7.131
7.127
6.812
6.808
6.791
6.783
6.778
6.767
6.762
6.755
6.729
6.725
6.720
6.709
6.707
6.703
3.456
2.172
MeOD
OH
CH3
N
OH
11g
1H-NMR, 400 MHz, MeOD.
H2O
11
10
9
8
7
6
5
4
3
2
1
ppm
2.97
3.73
0.92
4.00
3.01

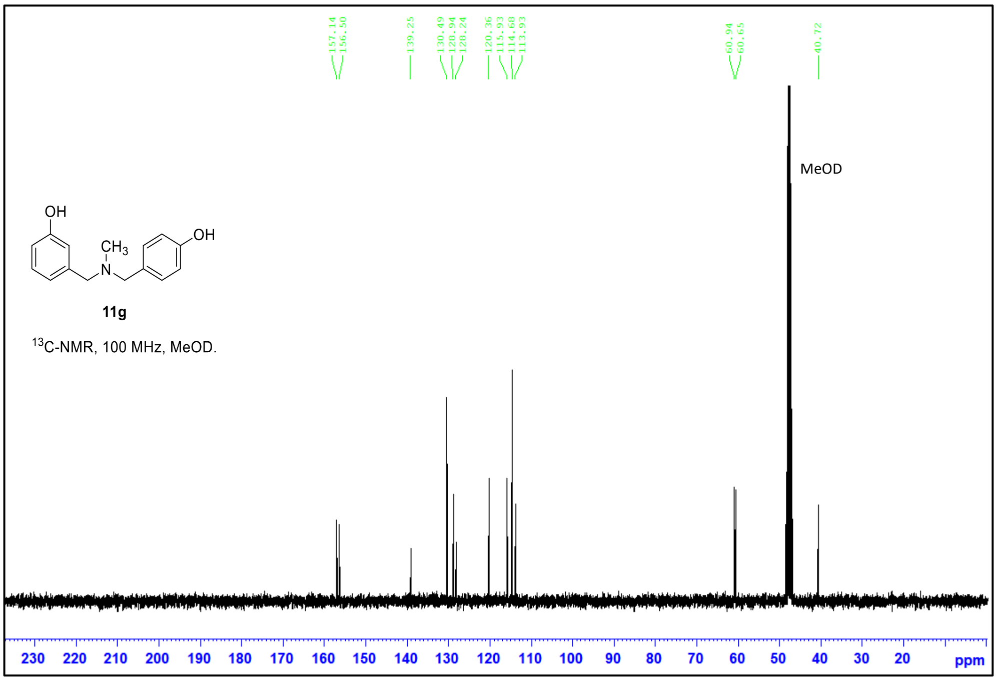
157.14
156.50
139.25
130.49
128.94
120.24
120.36
115.93
114.60
113.93
60.94
60.65
40.72
MeOD
OH
CH3
N
OH
11g
13C-NMR, 100 MHz, MeOD.
230
220
210
200
190
180
170
160
150
140
130
120
110
100
90
80
70
60
50
40
30
20
ppm

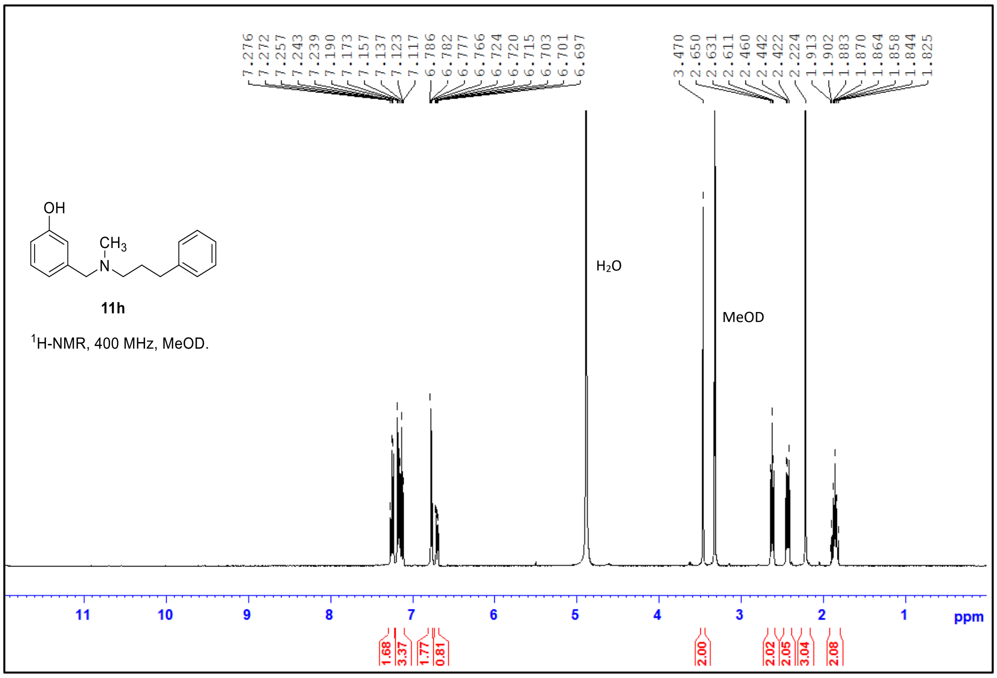

7.276
7.272
7.257
7.243
7.239
7.190
7.173
7.157
7.137
7.123
7.117
6.786
6.782
6.777
6.766
6.724
6.720
6.715
6.703
6.701
6.697
3.470
2.650
2.631
2.611
2.460
2.442
2.422
2.224
1.913
1.902
1.883
1.870
1.864
1.858
1.844
1.825
OH
CH3
N
11h
1H-NMR, 400 MHz, MeOD.
H2O
MeOD
1.68
3.37
1.77
0.81
2.00
2.02
2.05
3.04
2.08
ppm

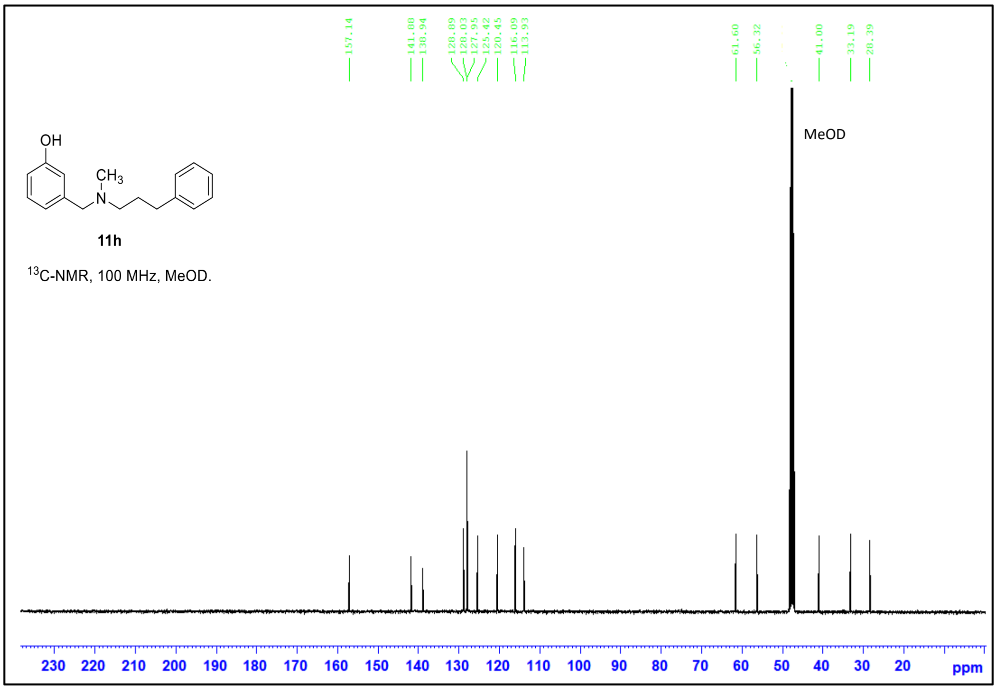

157.14
141.08
138.94
128.09
128.03
127.95
125.42
120.45
116.09
113.93
61.60
56.32
41.00
33.19
28.39
OH
CH3
N
11h
13C-NMR, 100 MHz, MeOD.
MeOD
ppm

*SM #3.3. NMR specra of compounds **9** and **12a-h***

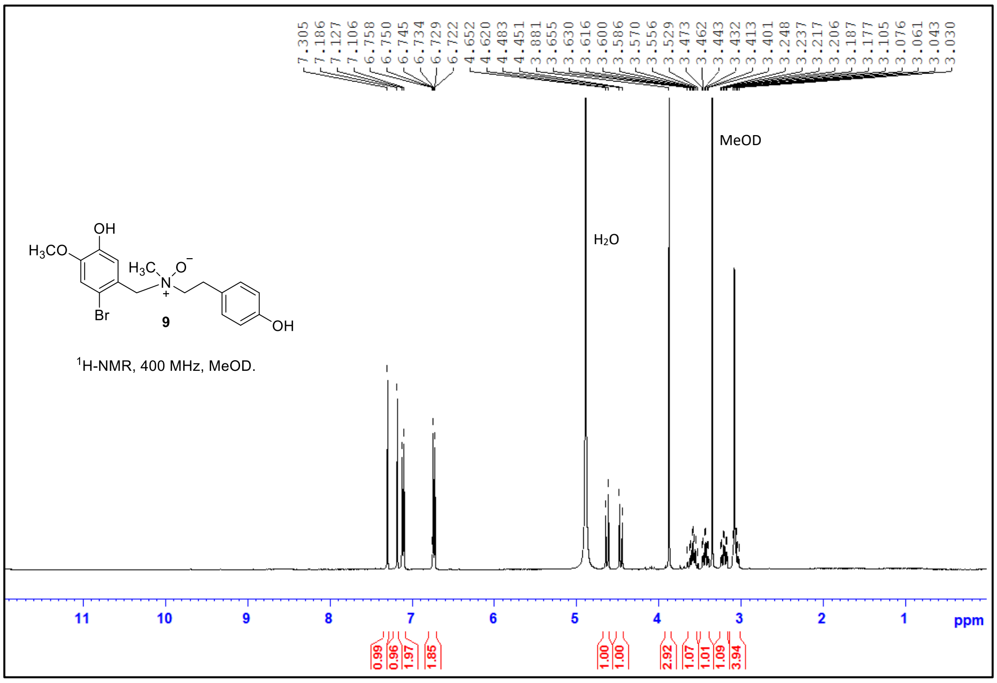

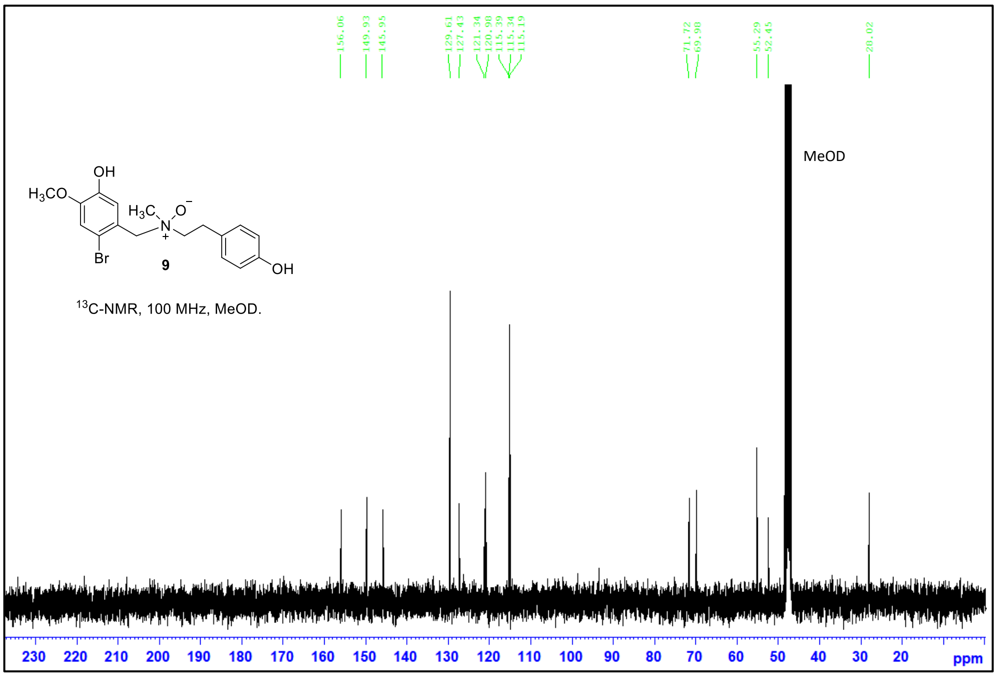

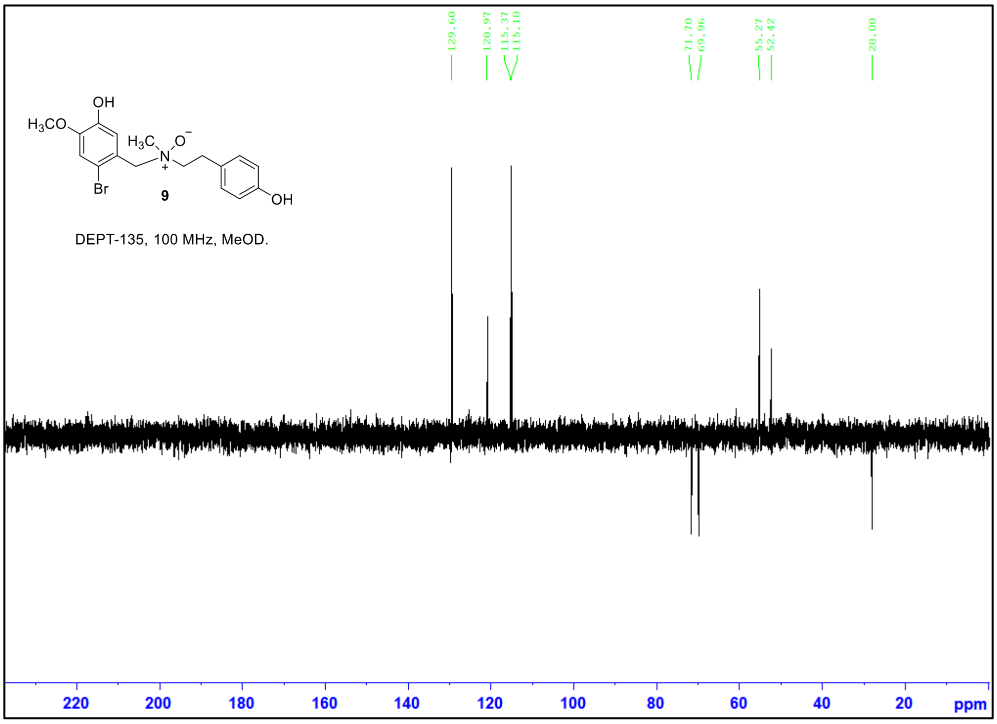
129.60
120.97
115.37
115.10
71.70
69.96
55.27
52.42
28.00
OH
H3CO
H3C
O−
N+
Br
9
OH
DEPT-135, 100 MHz, MeOD.
220 200 180 160 140 120 100 80 60 40 20 ppm

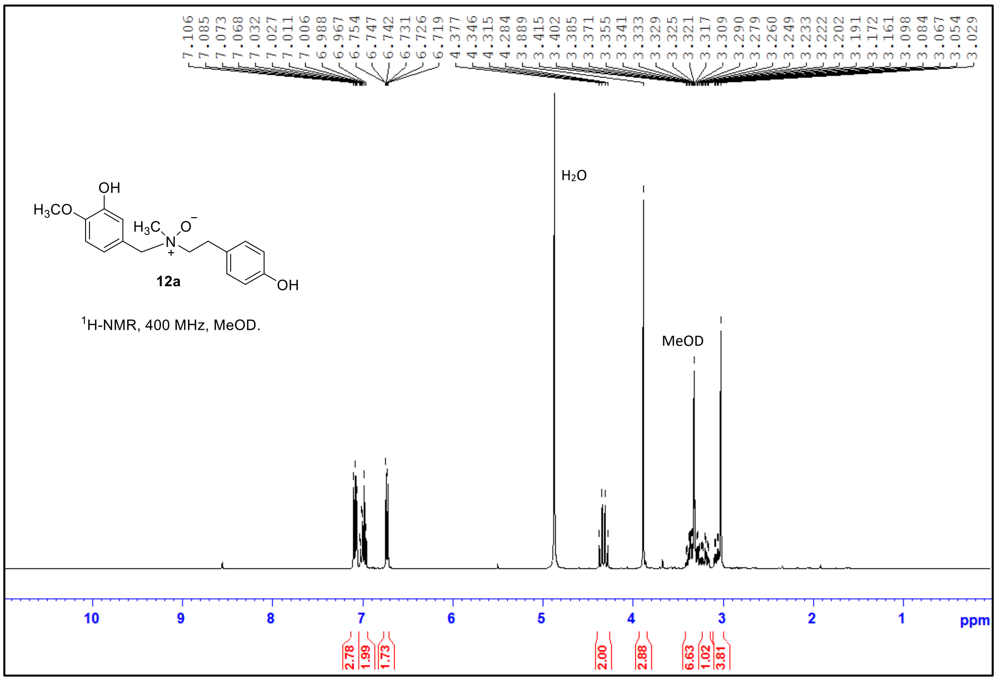
7.106 7.085 7.073 7.068 7.032 7.027 7.011 7.006 6.988 6.967 6.754 6.747 6.742 6.731 6.726 6.719 4.377 4.346 4.315 4.284 3.889 3.415 3.402 3.385 3.371 3.355 3.341 3.333 3.329 3.325 3.321 3.317 3.309 3.290 3.279 3.260 3.249 3.233 3.222 3.202 3.191 3.172 3.161 3.098 3.084 3.067 3.054 3.029
OH
H3CO
H3C
O−
N+
12a
OH
1H-NMR, 400 MHz, MeOD.
H2O
MeOD
10 9 8 7 6 5 4 3 2 1 ppm
2.78 1.99 1.73 2.00 2.88 6.63 1.02 3.81

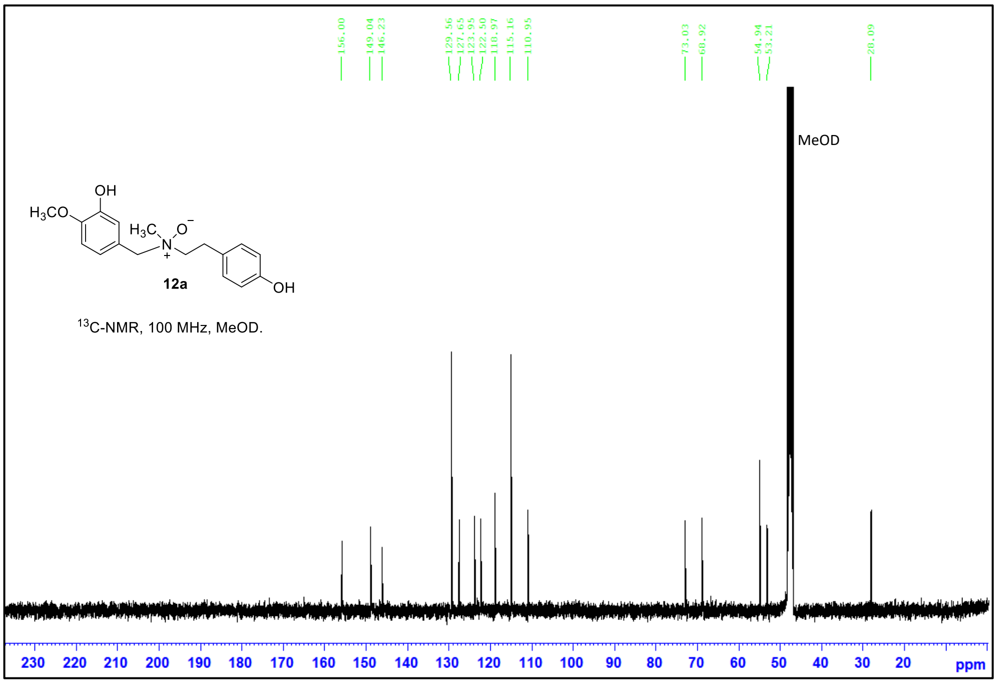
MeOD
12a
13C-NMR, 100 MHz, MeOD.
ppm

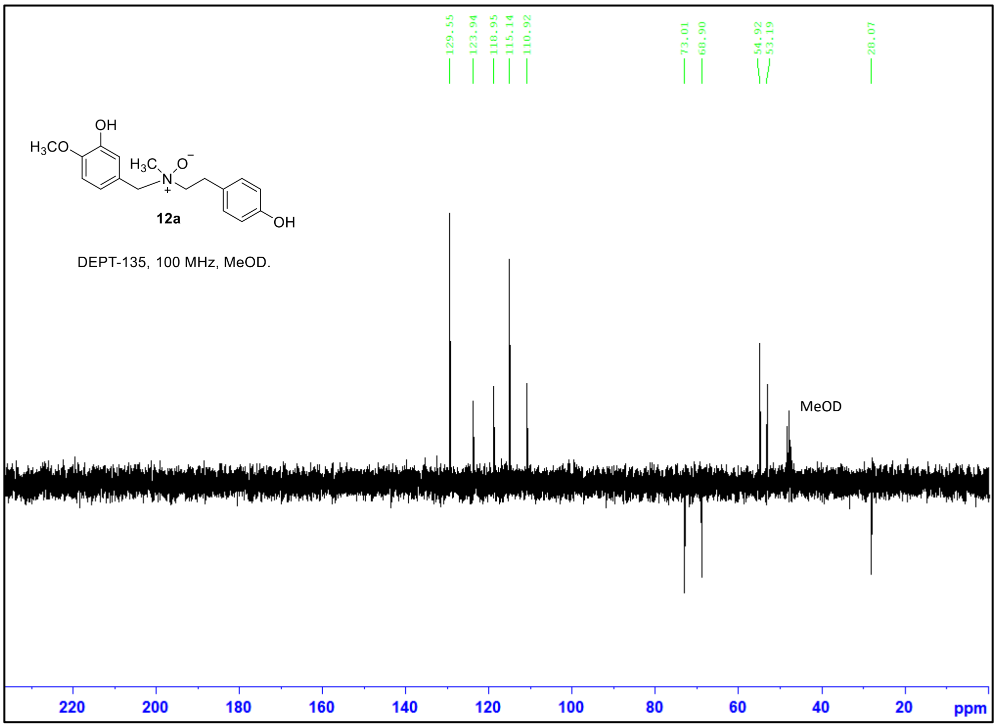
MeOD
12a
DEPT-135, 100 MHz, MeOD.
ppm

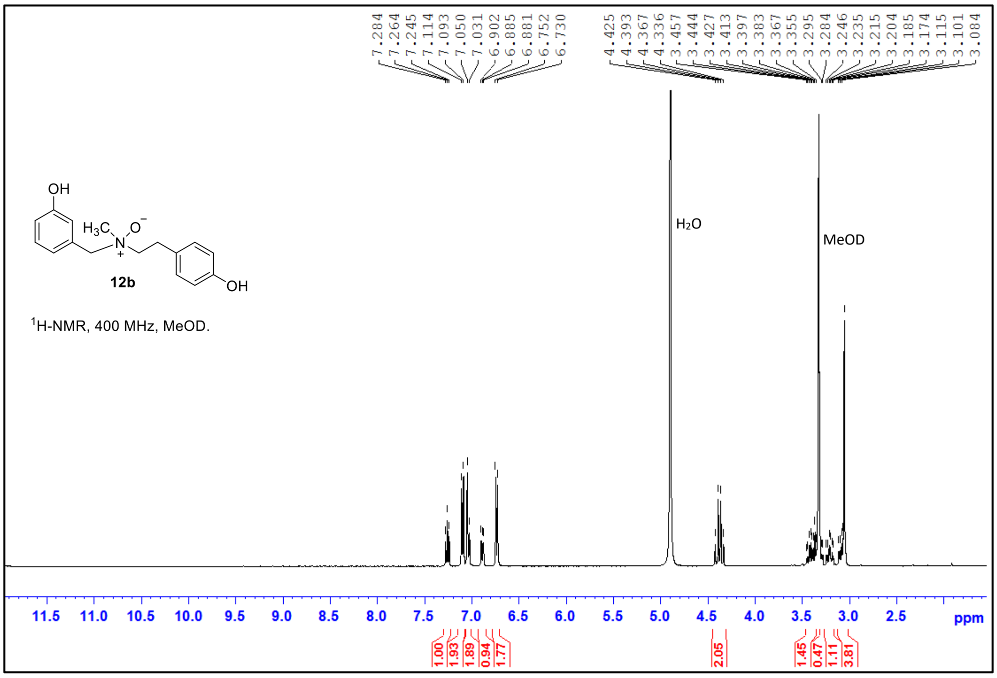

7.284
7.264
7.245
7.114
7.093
7.050
7.031
6.902
6.885
6.881
6.752
6.730
4.425
4.393
4.367
4.336
3.457
3.444
3.427
3.413
3.397
3.383
3.367
3.355
3.295
3.284
3.246
3.235
3.215
3.204
3.185
3.174
3.115
3.101
3.084
H2O
MeOD
12b
1H-NMR, 400 MHz, MeOD.
ppm
1.00
1.93
1.89
0.94
1.77
2.05
1.45
0.47
1.11
3.81

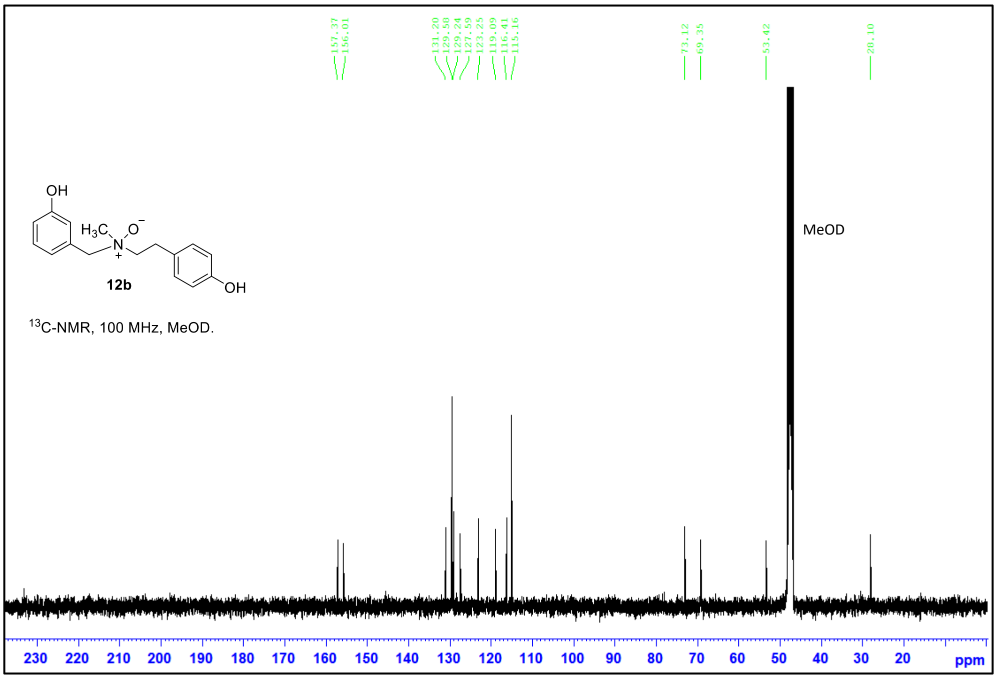

157.37
156.01
131.20
129.58
129.24
127.50
123.25
119.09
116.41
115.16
73.12
69.35
53.42
29.10
MeOD
12b
13C-NMR, 100 MHz, MeOD.
ppm

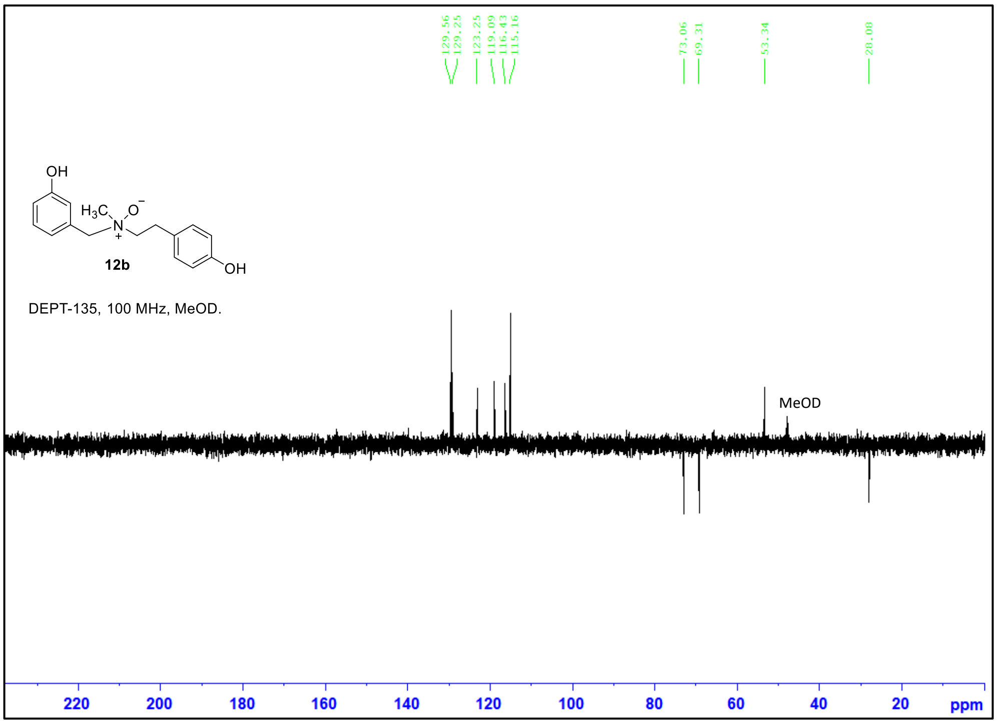

129.56
129.25
123.25
119.09
116.43
115.16
73.06
69.31
53.34
28.00
OH
H_3C
O
N
+
OH
12b
DEPT-135, 100 MHz, MeOD.
MeOD
220
200
180
160
140
120
100
80
60
40
20
ppm

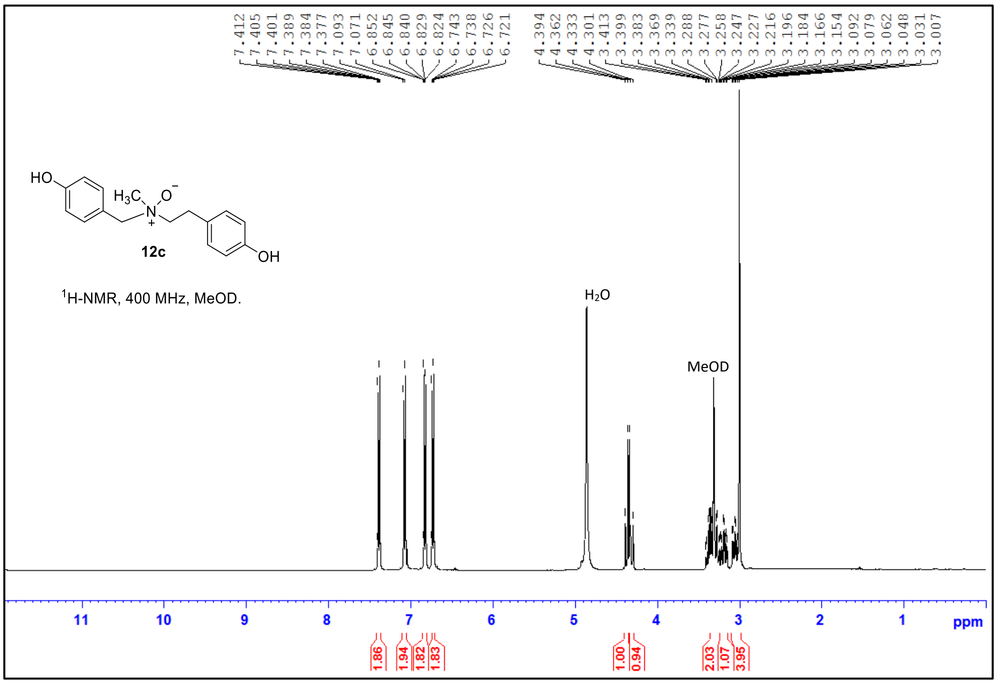

7.412
7.405
7.401
7.389
7.384
7.377
7.093
7.071
6.852
6.845
6.840
6.829
6.824
6.743
6.738
6.726
6.721
4.394
4.362
4.333
4.301
3.413
3.399
3.383
3.369
3.339
3.288
3.277
3.258
3.247
3.227
3.216
3.196
3.184
3.166
3.154
3.092
3.079
3.062
3.048
3.031
3.007
HO
H_3C
O
N
+
OH
12c
^{1}H-NMR, 400 MHz, MeOD.
H_2O
MeOD
11
10
9
8
7
6
5
4
3
2
1
ppm
1.86
1.94
1.82
1.83
1.00
0.94
2.03
1.07
3.95

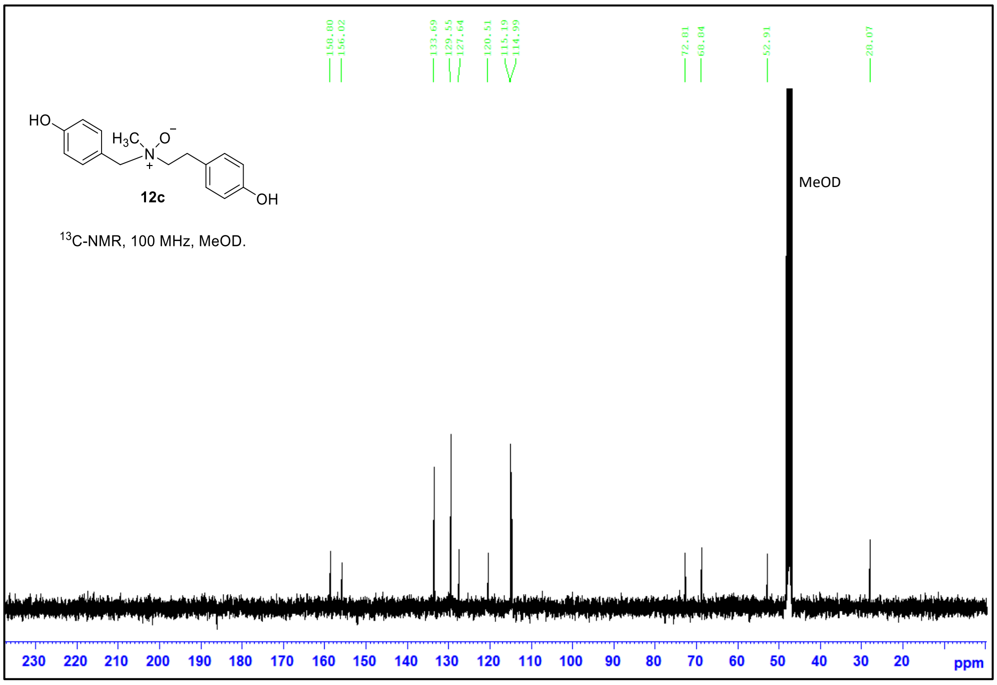
158.80
156.02
133.69
129.55
127.64
120.51
115.19
114.99
72.81
68.84
52.91
28.07
HO
H_3C
O
N
12c
OH
MeOD
^{13}C-NMR, 100 MHz, MeOD.
230 220 210 200 190 180 170 160 150 140 130 120 110 100 90 80 70 60 50 40 30 20 ppm

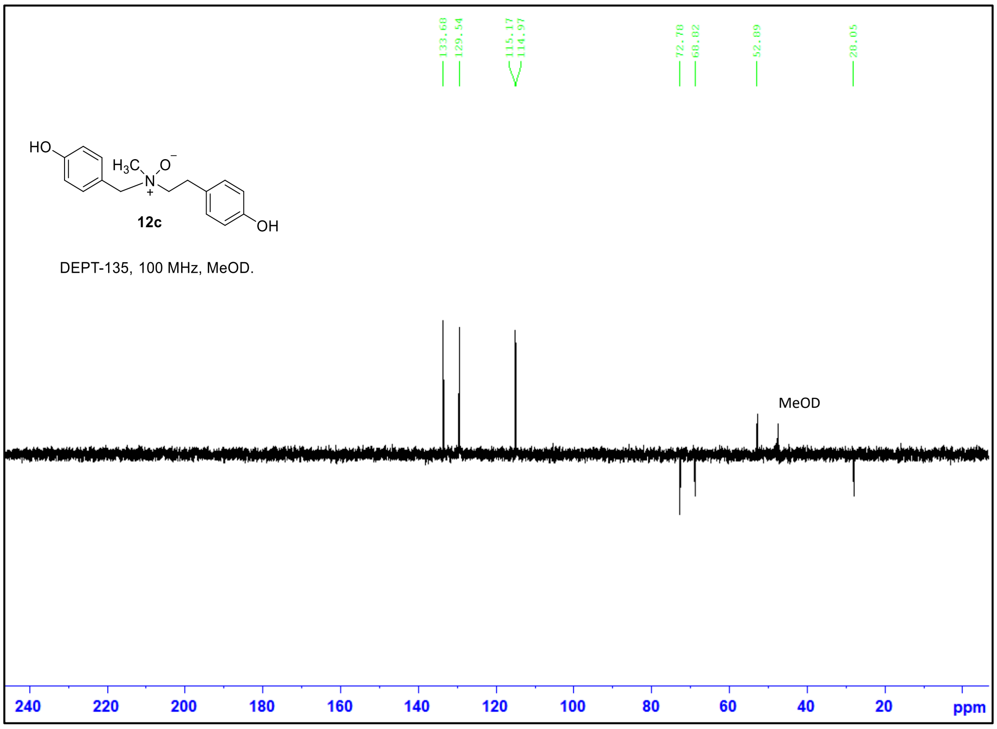
133.68
129.54
115.17
114.97
72.78
68.82
52.89
28.05
HO
H_3C
O
N
12c
OH
DEPT-135, 100 MHz, MeOD.
MeOD
240 220 200 180 160 140 120 100 80 60 40 20 ppm

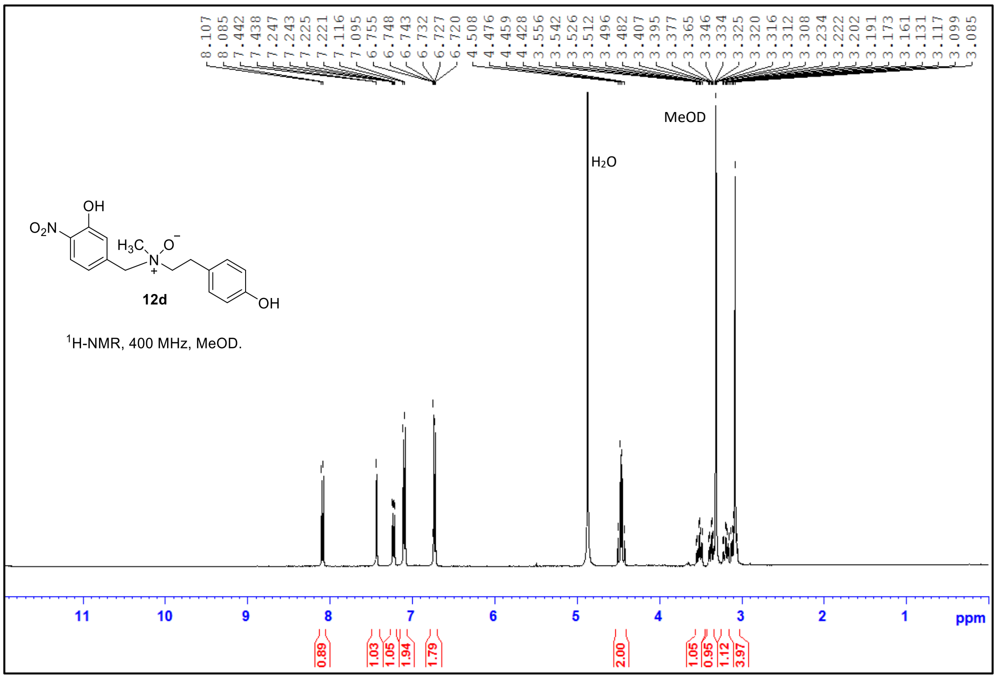
8.107
8.085
7.442
7.438
7.247
7.243
7.225
7.221
7.116
7.095
6.755
6.748
6.743
6.732
6.727
6.720
4.508
4.476
4.459
4.428
3.556
3.542
3.526
3.512
3.496
3.482
3.407
3.395
3.377
3.365
3.346
3.334
3.325
3.320
3.316
3.312
3.308
3.234
3.222
3.202
3.191
3.173
3.161
3.131
3.117
3.099
3.085
MeOD
H2O
OH
O2N
H3C
O
N
OH
12d
1H-NMR, 400 MHz, MeOD.
11
10
9
8
7
6
5
4
3
2
1
ppm
0.89
1.03
1.05
1.94
1.79
2.00
1.05
0.95
1.12
3.97

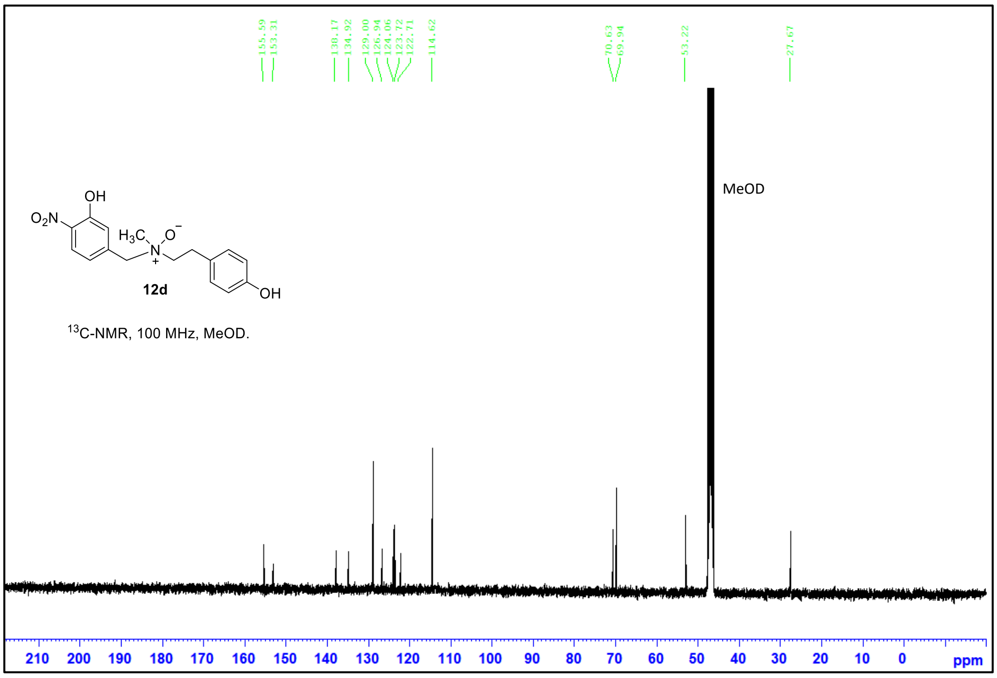
155.59
153.31
139.17
134.92
129.00
126.94
124.06
123.72
122.71
114.62
70.63
69.94
53.22
27.67
MeOD
OH
O2N
H3C
O
N
OH
12d
13C-NMR, 100 MHz, MeOD.
210
200
190
180
170
160
150
140
130
120
110
100
90
80
70
60
50
40
30
20
10
0
ppm

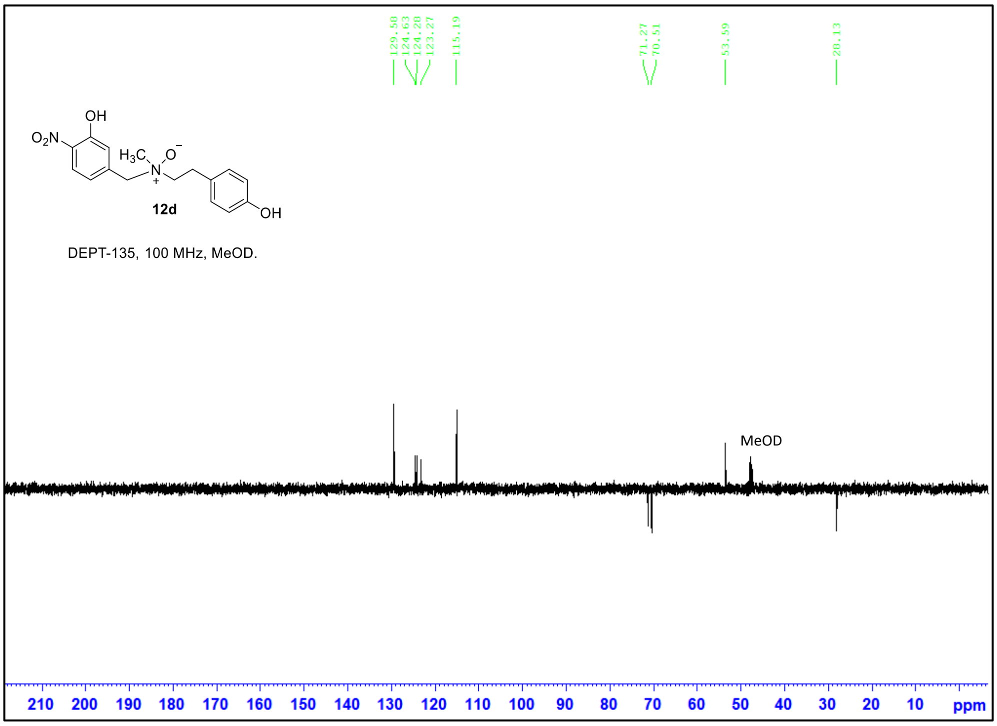
129.58
124.63
124.28
123.27
115.19
71.27
70.51
53.59
28.13
OH
O_2N
H_3C
N
O
OH
12d
DEPT-135, 100 MHz, MeOD.
MeOD
210 200 190 180 170 160 150 140 130 120 110 100 90 80 70 60 50 40 30 20 10 ppm

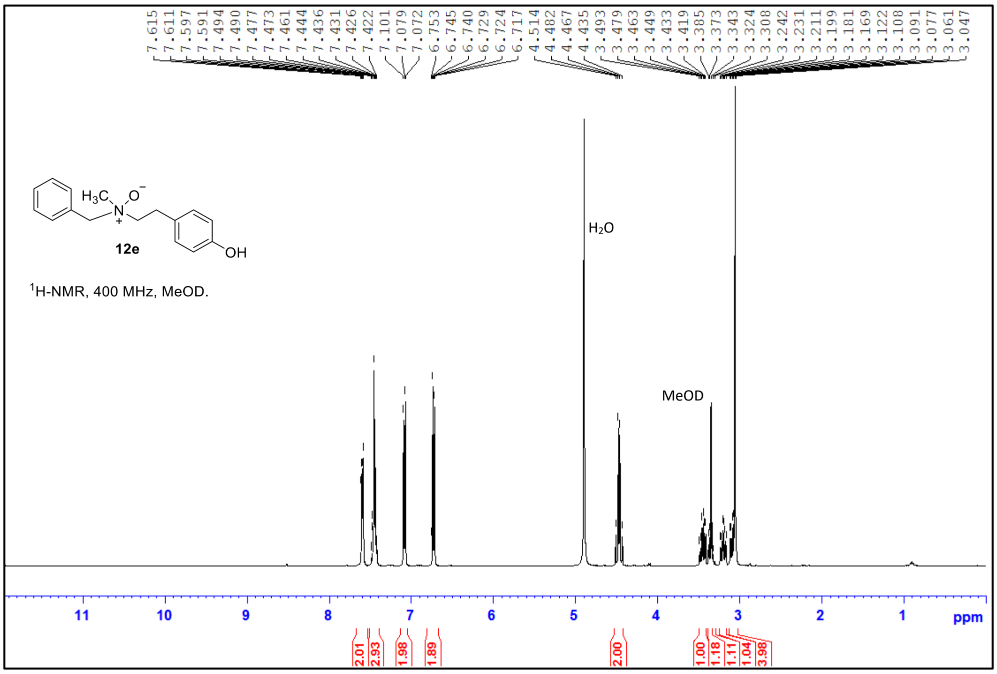
7.615 7.611 7.597 7.591 7.494 7.490 7.477 7.473 7.461 7.444 7.436 7.431 7.426 7.422 7.101 7.079 7.072 6.753 6.745 6.740 6.729 6.724 6.717 4.514 4.482 4.467 4.435 3.493 3.479 3.463 3.449 3.433 3.419 3.385 3.373 3.343 3.324 3.308 3.242 3.231 3.211 3.199 3.181 3.169 3.122 3.108 3.091 3.077 3.061 3.047
H_3C
N
O
OH
12e
^{1}H-NMR, 400 MHz, MeOD.
H_2O
MeOD
11 10 9 8 7 6 5 4 3 2 1 ppm
2.01 2.93 1.98 1.89 2.00 1.00 1.18 1.11 1.04 3.98

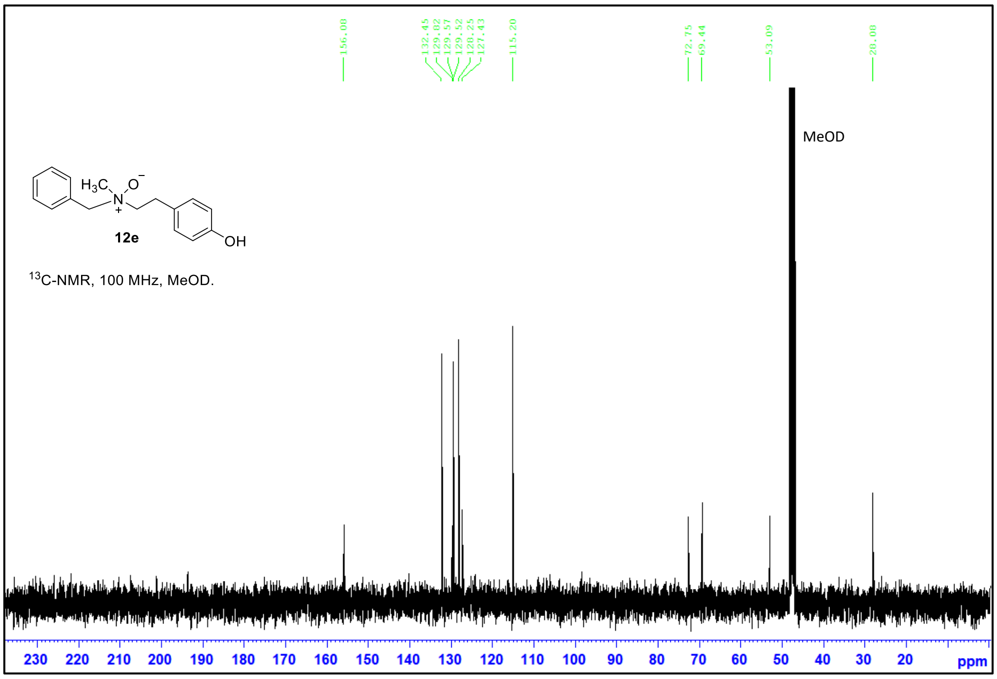
MeOD
12e
OH
^{13}C-NMR, 100 MHz, MeOD.
ppm

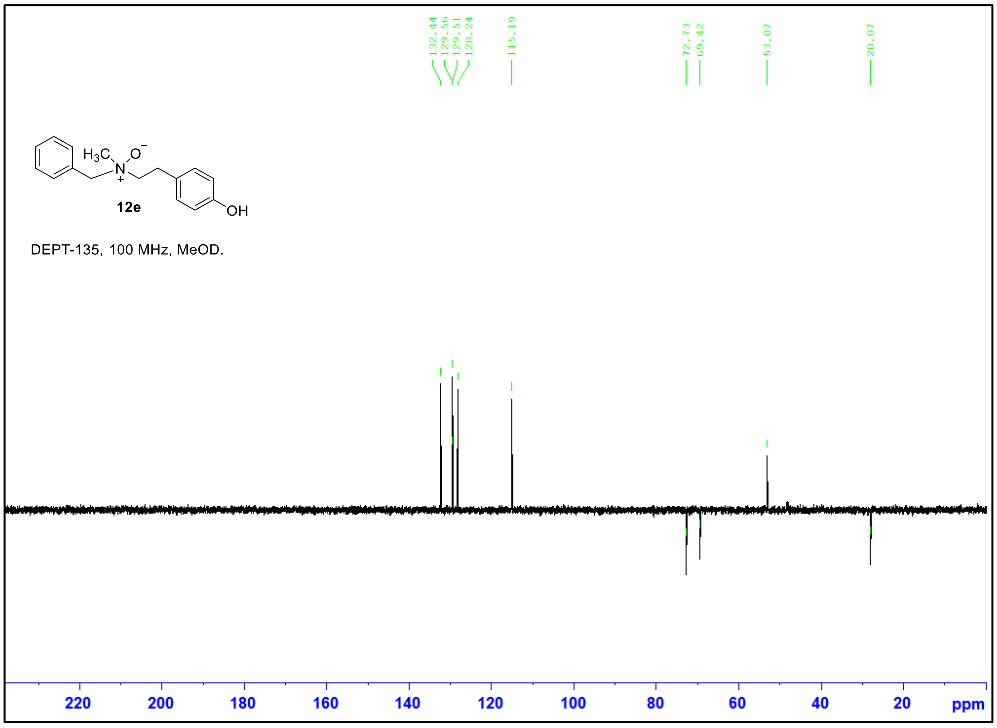
12e
OH
DEPT-135, 100 MHz, MeOD.
ppm

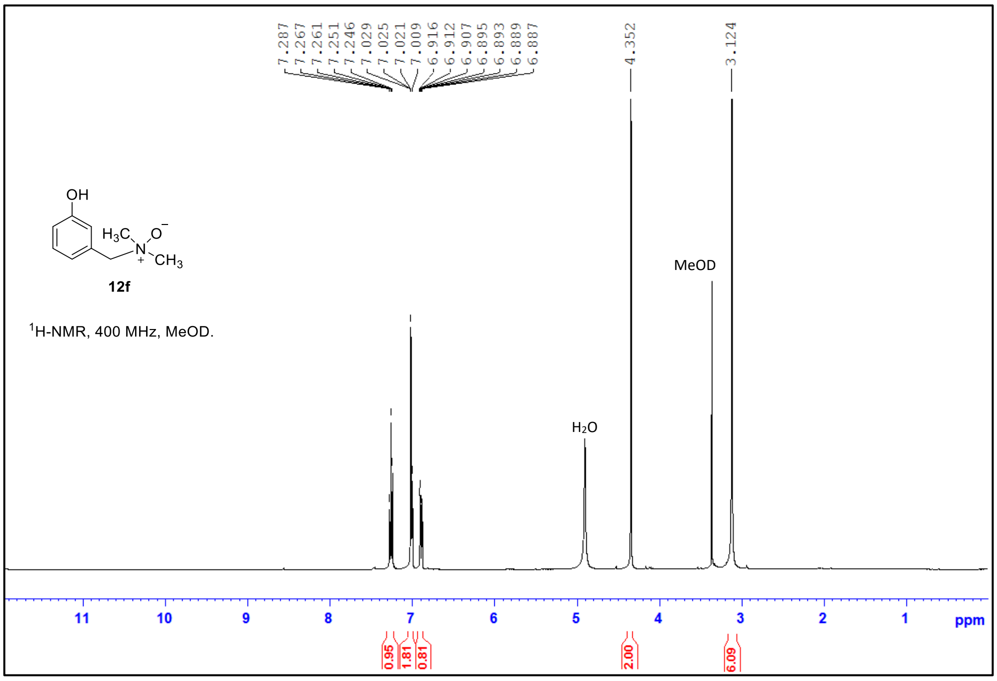
7.287
7.267
7.261
7.251
7.246
7.029
7.025
7.021
7.009
6.916
6.912
6.907
6.895
6.893
6.889
6.887
4.352
3.124
OH
H_3C
O
N
CH_3
12f
^{1}H-NMR, 400 MHz, MeOD.
MeOD
H_2O
11
10
9
8
7
6
5
4
3
2
1
ppm
0.95
1.81
0.81
2.00
6.09

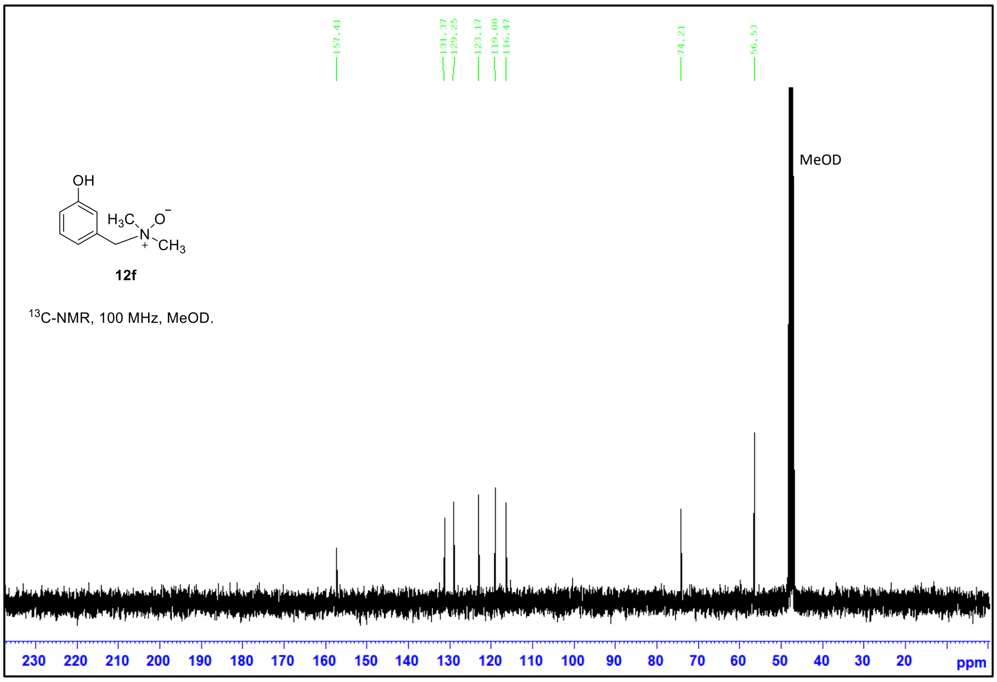
157.41
131.37
129.25
123.17
119.08
116.47
74.21
56.53
MeOD
OH
H_3C
O
N
CH_3
12f
^{13}C-NMR, 100 MHz, MeOD.
230
220
210
200
190
180
170
160
150
140
130
120
110
100
90
80
70
60
50
40
30
20
ppm

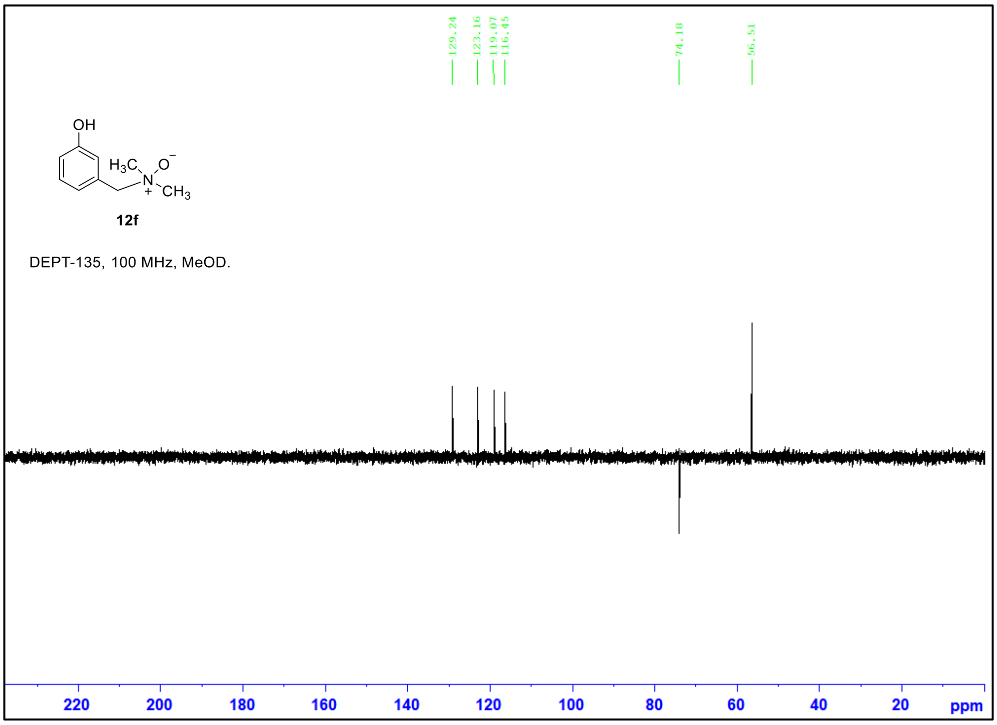
129.24
123.16
119.07
116.45
74.18
56.51
OH
H3C
O−
N+
CH3
12f
DEPT-135, 100 MHz, MeOD.
220 200 180 160 140 120 100 80 60 40 20 ppm

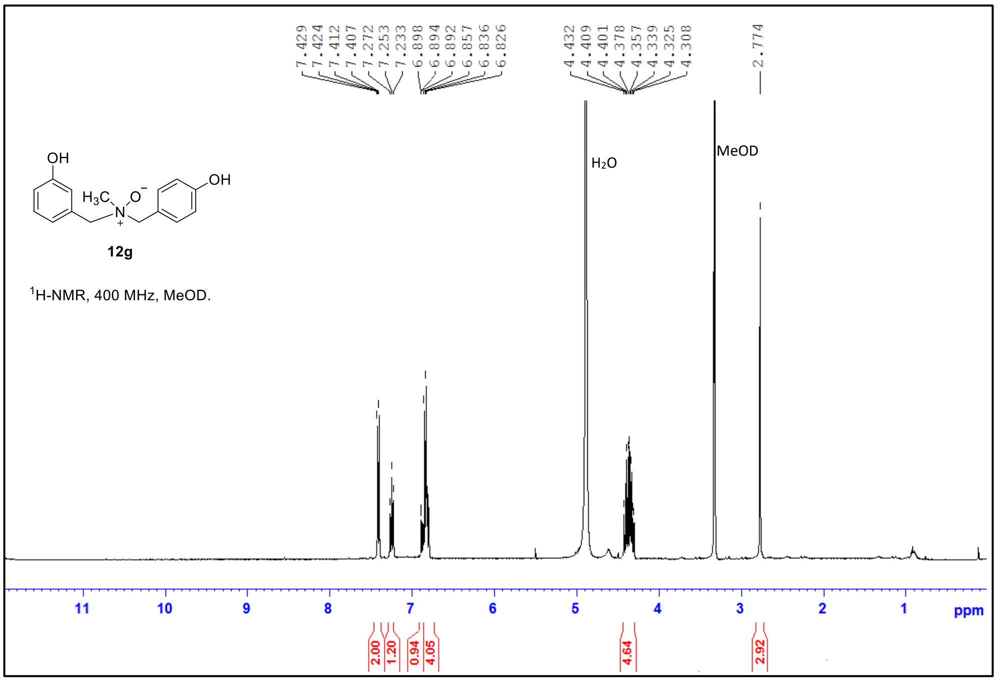
7.429 7.424 7.412 7.407 7.272 7.253 7.233 6.898 6.894 6.892 6.857 6.836 6.826
4.432 4.409 4.401 4.378 4.357 4.339 4.325 4.308
2.774
H2O
MeOD
OH
H3C
O−
N+
OH
12g
1H-NMR, 400 MHz, MeOD.
11 10 9 8 7 6 5 4 3 2 1 ppm
2.00 1.20 0.94 4.05 4.64 2.92

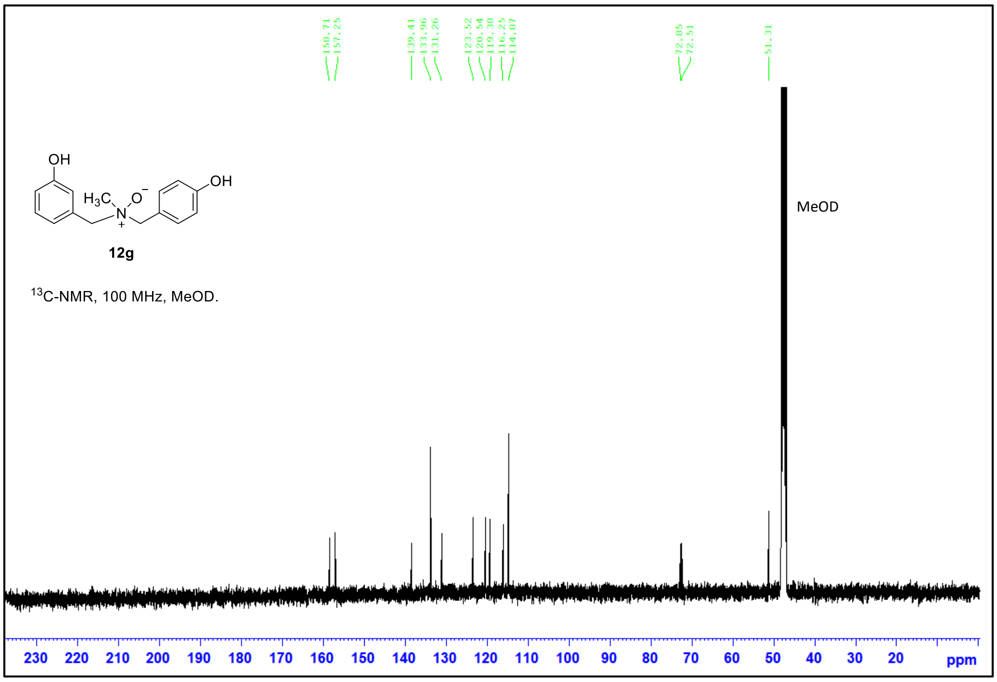

150.71
157.25
139.41
133.96
131.26
123.52
120.54
119.30
116.25
114.87
72.65
72.51
51.31
OH
H_3C
O
OH
MeOD
12g
^{13}C-NMR, 100 MHz, MeOD.
230 220 210 200 190 180 170 160 150 140 130 120 110 100 90 80 70 60 50 40 30 20 ppm

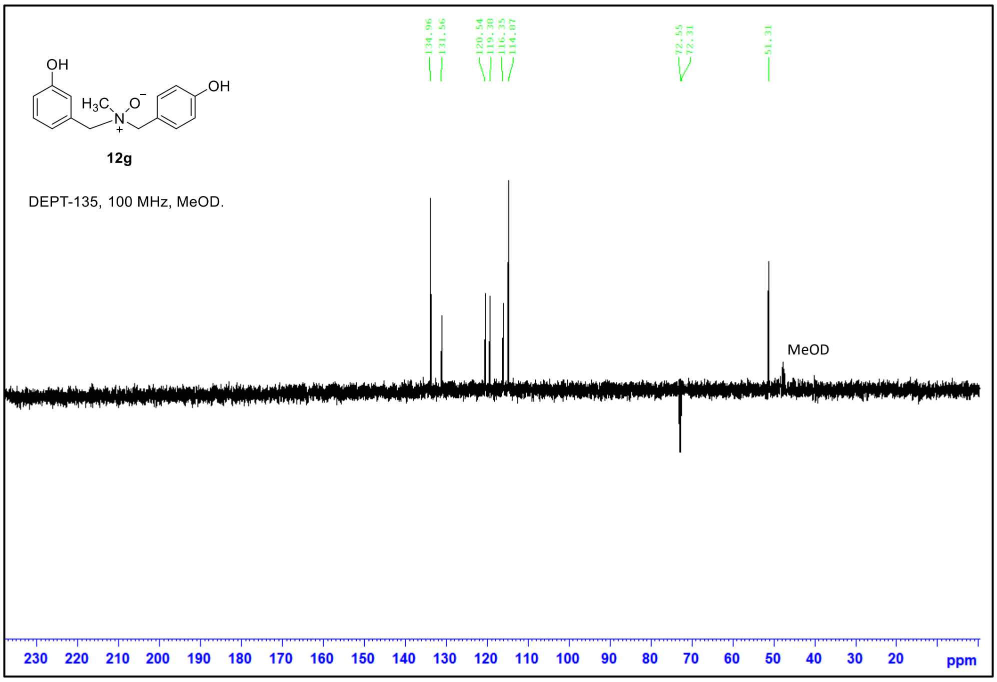

134.96
131.56
120.54
119.30
116.35
114.87
72.55
72.31
51.31
OH
H_3C
O
OH
12g
DEPT-135, 100 MHz, MeOD.
MeOD
230 220 210 200 190 180 170 160 150 140 130 120 110 100 90 80 70 60 50 40 30 20 ppm

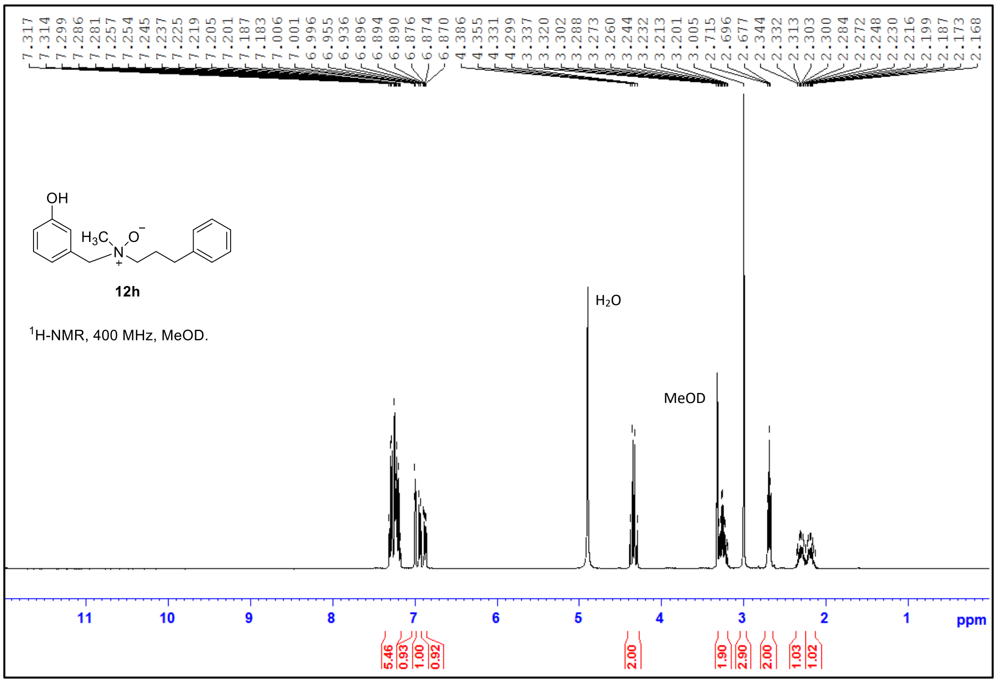
OH
H_3C
O
N
12h
^{1}H-NMR, 400 MHz, MeOD.
H$_2$O
MeOD
ppm

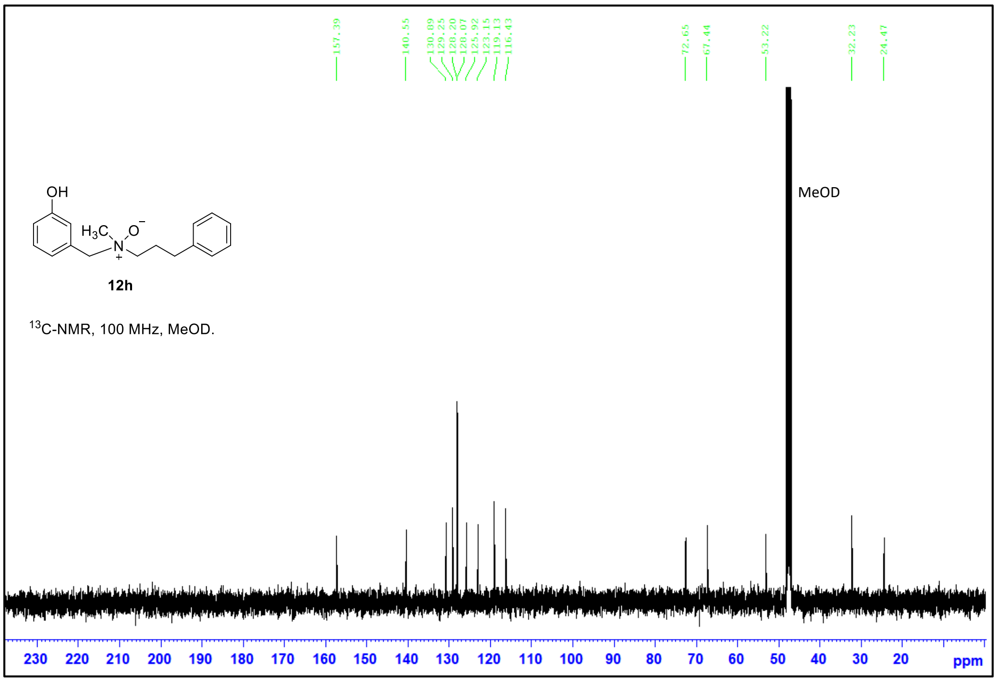
157.39
140.55
130.09
129.25
128.20
128.07
125.92
123.15
119.13
116.43
72.65
67.44
53.22
32.23
24.47
OH
H_3C
O
N
12h
^{13}C-NMR, 100 MHz, MeOD.
MeOD
ppm

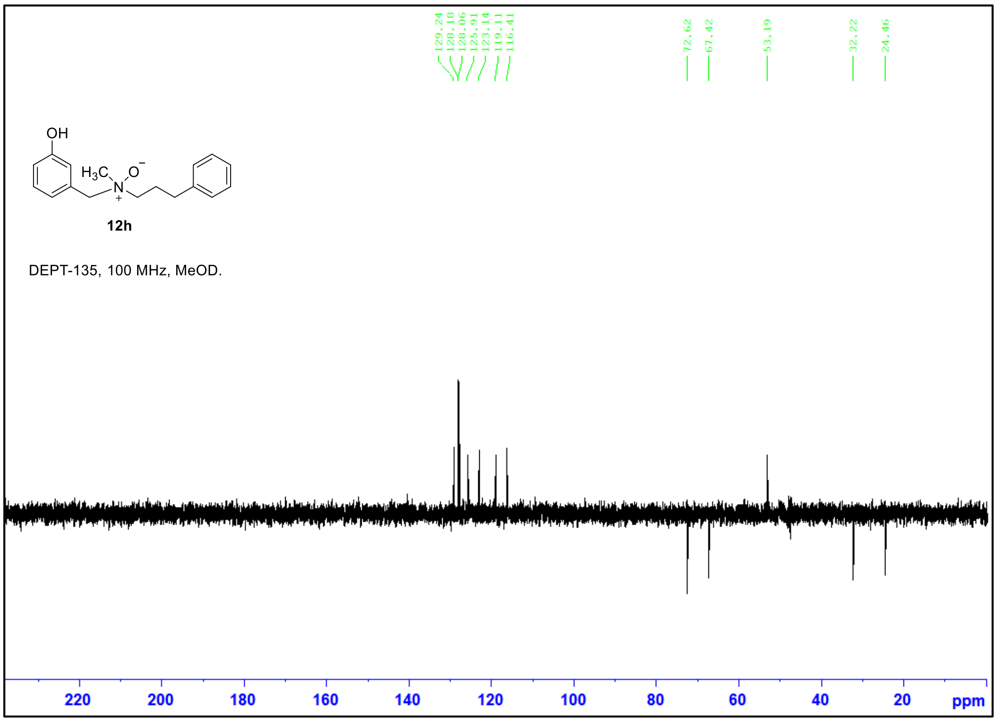

SM #3.1. NMR specra of compound ***10***

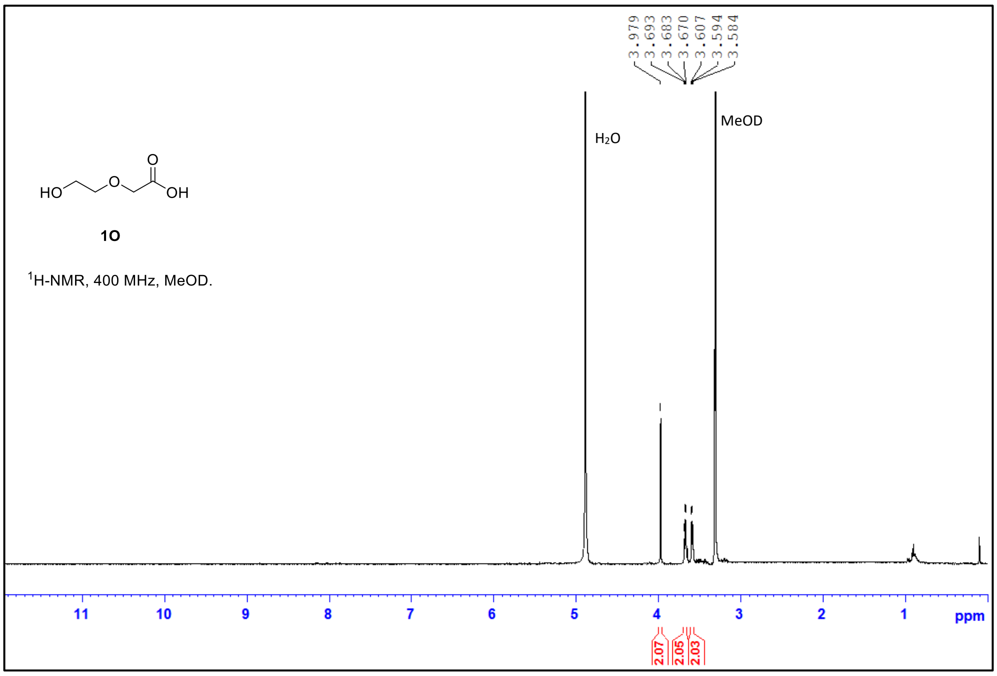

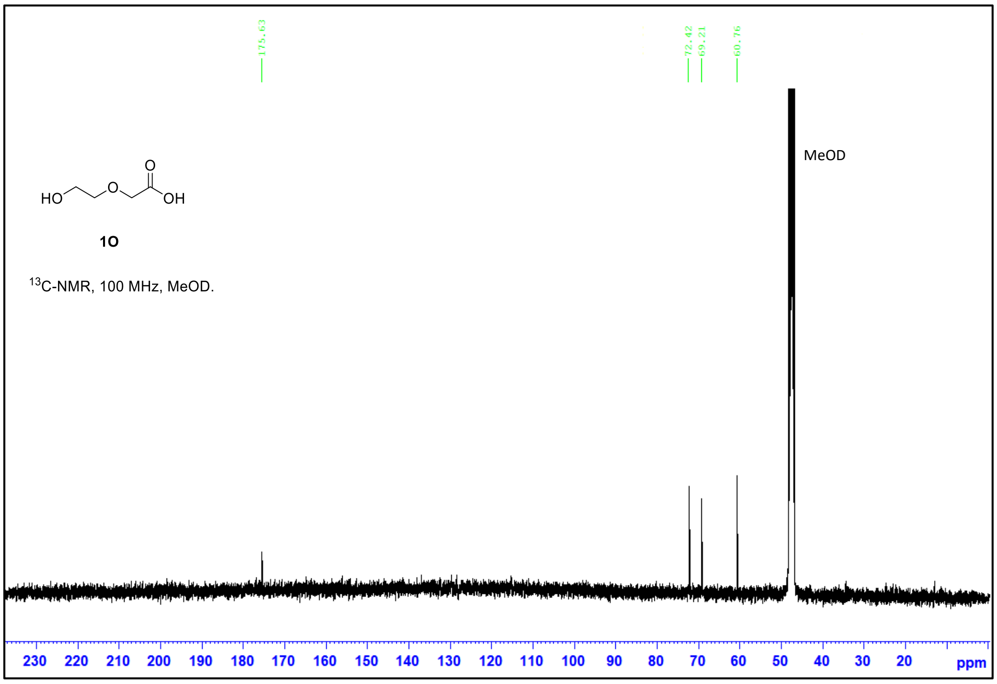
175.63
72.42
69.21
60.76
MeOD
HO
O
O
OH
1O
13C-NMR, 100 MHz, MeOD.
230
220
210
200
190
180
170
160
150
140
130
120
110
100
90
80
70
60
50
40
30
20
ppm

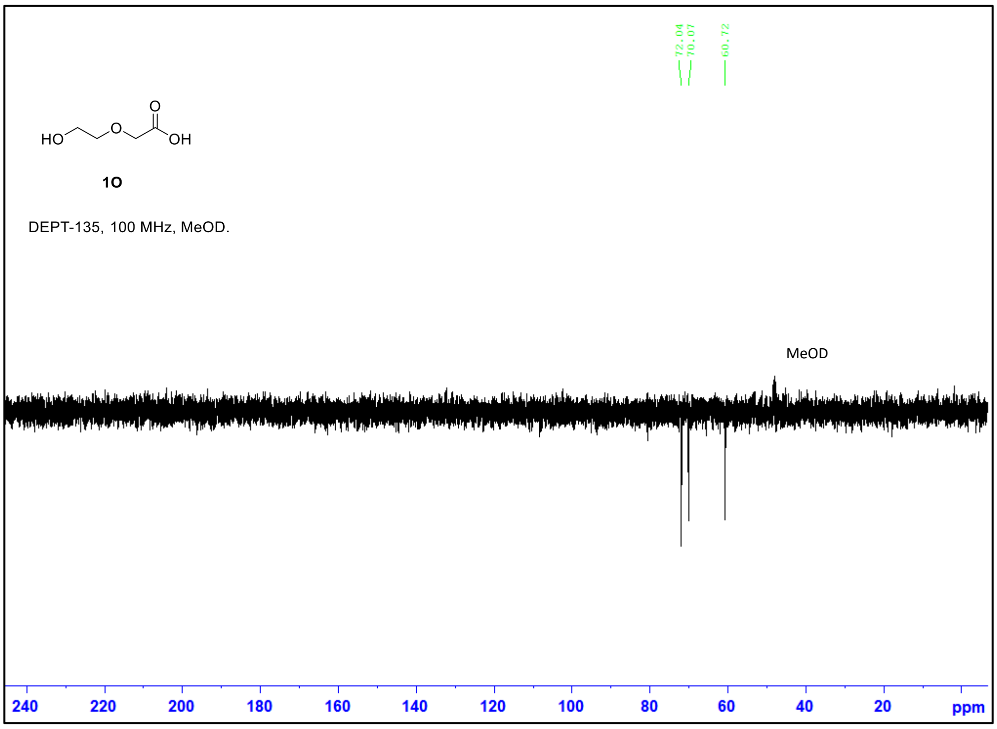
72.04
70.07
60.72
HO
O
O
OH
1O
DEPT-135, 100 MHz, MeOD.
MeOD
240
220
200
180
160
140
120
100
80
60
40
20
ppm
